# HMGA2 alleviates ferroptosis by promoting GPX4 expression in pancreatic cancer cells

**DOI:** 10.1038/s41419-024-06592-y

**Published:** 2024-03-16

**Authors:** Ziyang Luo, Qingfang Zheng, Shazhou Ye, Yanguo Li, Jiayi Chen, Chengjiang Fan, Jianing Chen, Yuxin Lei, Qi Liao, Yang Xi

**Affiliations:** 1 Department of Biochemistry and Molecular Biology and Zhejiang Key Laboratory of Pathophysiology, School of Basic Medical Sciences, Health Science Center, Ningbo University, Ningbo, 315211 China; 2 Institute of Drug Discovery Technology, Ningbo University, Ningbo, 315211 China

**Keywords:** Oncogenes, Chemotherapy

## Abstract

Pancreatic cancer is one of the most malignant tumor types and is characterized by high metastasis ability and a low survival rate. As a chromatin-binding protein, HMGA2 is widely overexpressed and considered an oncogene with various undefined regulatory mechanisms. Herein, we demonstrated that HMGA2 is highly expressed in pancreatic cancer tissues, mainly distributed in epithelial cells, and represents a subtype of high epithelial–mesenchymal transition. Deletion of HMGA2 inhibits tumor malignancy through cell proliferation, metastasis, and xenograft tumor growth in vivo. Moreover, HMGA2 enhanced the cellular redox status by inhibiting reactive oxygen species and promoting glutathione production. Importantly, ferroptotic cell death was significantly ameliorated in cells overexpressing HMGA2. Conversely, HMGA2 deletion exacerbated ferroptosis. Mechanistically, HMGA2 activated GPX4 expression through transcriptional and translational regulation. HMGA2 binds and promotes cis-element modification in the promoter region of the GPX4 gene by enhancing enhancer activity through increased H3K4 methylation and H3K27 acetylation. Furthermore, HMGA2 stimulated GPX4 protein synthesis via the mTORC1-4EBP1 and -S6K signaling axes. The overexpression of HMGA2 alleviated the decrease in GPX4 protein levels resulting from the pharmacologic inhibition of mTORC1. Conversely, compared with the control, HMGA2 deletion more strongly reduced the phosphorylation of 4EBP1 and S6K. A strong positive correlation between HMGA2 and GPX4 expression was confirmed using immunohistochemical staining. We also demonstrated that HMGA2 mitigated the sensitivity of cancer cells to combination treatment with a ferroptosis inducer and mTORC1 inhibition or gemcitabine. In summary, our results revealed a regulatory mechanism by which HMGA2 coordinates GPX4 expression and underscores the potential value of targeting HMGA2 in cancer treatment.

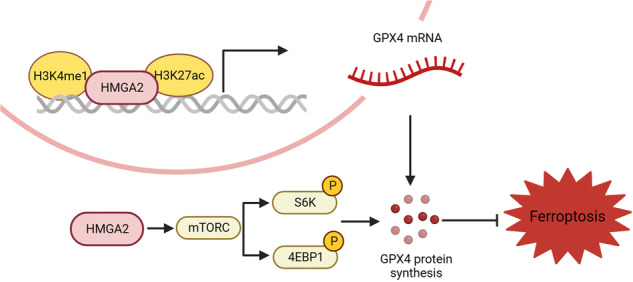

## Introduction

Pancreatic cancer is one of the most malignant types of tumors and the fourth leading cause of cancer-related death in both sexes [1]. Pancreatic cancer often goes undetected with rapid progression until it reaches an advanced stage and spreads, making it difficult to diagnose in time [2]. The clinical treatments for pancreatic cancer can be divided into three parts: surgery, chemotherapy, and radiotherapy [3]. Because of the high radio-resistance of pancreatic cancer and the inability of adjacent organs to tolerate high doses of radiation, the only method for curing pancreatic cancer is the combination of complete resection and systemic chemotherapy. However, recent studies have suggested that pancreatic cancer has a heterogeneous cell composition and a cold tumor microenvironment [4], leading to resistance to neoadjuvant chemotherapy and/or radiotherapy [5].

Ferroptosis, a recently discovered form of regulated cell death, is iron-dependent and results from excessive lipid peroxidation. Glutathione peroxidase 4 (GPX4) is a type of selenoenzyme that contains cysteine and functions as a redox-active enzyme [6]. By reducing the abundance of oxidative substrates and detoxifying lipid peroxidation products, GPX4 protects cells from oxidative damage and inhibits ferroptosis. The antioxidative function of GPX4 depends on cysteine, a redox-active residue located in the catalytic site. The primary pathway for acquiring cysteine in cancer cells involves amino acid transporter solute carrier family 7 membrane 11 (SLC7A11), which transfers extracellular cystine to the intracellular compartment, where it is reduced to cysteine [7]. GPX4 knockout mice experience stillbirth during embryonic development [6, 8], and sperm lacking GPX4 are highly susceptible to oxidative damage, resulting in infertility [9]. Protein synthesis is an energy-intensive process that requires close coordination with nutrient and energy supplies, particularly selenoproteins [10]. The integration of environmental and nutritional factors to regulate protein synthesis plays a pivotal role in this process. Cancer cells can regulate gene expression to adapt to different stress conditions, whether they originate internally or from external stimuli, and selectively synthesize proteins that are most urgently needed [11, 12]. The mechanistic target of rapamycin complex (mTORC) is a critical eukaryotic signaling network that coordinates cell physiology with environmental conditions [13]. In protein synthesis, mTORC1 phosphorylates two key effectors, p70S6 kinase 1 (S6K) and eIF4E-binding protein (4EBP), to enhance mRNA translation. Phosphorylated S6K can activate several substrates that promote mRNA translation initiation [14, 15]. In addition, mTORC1 directly phosphorylates 4EBP, thereby promoting 5’ cap-dependent transcription [16].

High mobility group AT-hook 2 (HMGA2) belongs to the high mobility group A (HMGA) family [17]. HMGA proteins can regulate the transcriptional activity of several genes by interacting with transcriptional regulators and altering chromatin structure, thus affecting various biological processes [18]. HMGA2 protein is highly expressed during embryonic development but is poorly expressed in adult tissues. However, it is significantly increased in tumor cells [19]. The carcinogenic activity of HMGA2 involves several mechanisms, including its contribution to cell cycle progression. HMGA2 competitively inhibits retinoblastoma protein (RB), a tumor suppressor protein, through interaction with RB via the second AT-hook domain, promoting cell cycle progression [20, 21]. Moreover, HMGA2 can directly bind to its promoter region to promote cyclin A expression, further contributing to cell cycle progression [16]. In cancer treatment, HMGA2 can assist cancer cells in repairing DNA damage and overcoming cell cycle repression induced by drugs. Epithelial–mesenchymal transition (EMT) is a key factor in cancer progression and drug resistance [22, 23]. In a previous study, HMGA2 was identified as the upstream regulatory molecule of the TGFβ/Smad pathway, enhancing the phosphorylation of Smad3 to induce EMT [24]. In pancreatic cancer, HMGA2 is related to E-cadherin, vimentin, and N-cadherin, which are the characteristic changes associated with EMT [25]. Decreased drug sensitivity is the leading cause of treatment failure in cancer, particularly in pancreatic cancer. The functions of HMGA2, such as DNA repair and the promotion of EMT, can be exploited by cancer cells. Furthermore, a recent study reported that HMGA2 enhances gastric cancer cell survival via the mTOR/P-gp pathway [26], indicating the role of HMGA2 in regulating protein synthesis.

In this study, we demonstrated that HMGA2 promotes pancreatic cancer cell growth and exhibits antiferroptotic effects by activating the mTORC1 pathway and enhancing GPX4 translation. This reveals crosstalk between HMGA2 GPX4 levels and ferroptosis.

## Materials and methods

### Data collection and processing

Bulk gene transcriptome data and clinical information for pancreatic cancer patients were obtained from the Gene Expression Omnibus (GEO) and the International Cancer Genome Consortium (ICGC). The datasets GSE28735, GSE57495, GSE62452, GSE71729, GSE79668, GSE84219, GSE85916, GSE124230, and GSE172356 were obtained from GEO. The projects from the ICGC were PACA-AU, PACA-CA, PAAD-US, and PAEN-AU. Batch correction of the expression data (log2(FPKM + 1)) was performed using the ComBat function from the sva package (R package version 3.50.0) [27] in R language. Bulk proteomics data and clinical information for pancreatic ductal adenocarcinoma (PDAC) patients were obtained from the Clinical Proteomic Tumor Analysis Consortium (CPTAC). Single-cell RNA sequencing data of human pancreatic cancer [28] were downloaded from GEO with accession ID GSE155698, which contains 16 PDA tissue samples, 3 adjacent normal pancreas samples, 16 PBMC samples isolated from human PDA patients, and 4 PBMC samples isolated from healthy volunteers. The gene expression matrices were combined and analyzed using the Seurat R package [29]. We performed quality filtering to remove cells with fewer than 500 expressed genes, fewer than 500 unique molecular identifiers (UMIs), more than 10% UMIs derived from mitochondrial genes, and log10(expressed genes/UMIs) greater than 0.75. Then, we normalized and scaled the gene expression matrices using SCTransform. Subsequently, the Harmony algorithm was employed to integrate scRNA-seq data across different patients [30]. For cell type identification, we first applied a graph-based clustering approach by using the FindNeighbors and FindClusters functions. Then, the clusters were further annotated into seven major cell types according to the original article. The epithelial subpopulation was also identified by a pipeline similar to that described above. The differentially expressed genes in different cell lineages were calculated using the Seurat FindMarkers function with the “MAST” algorithm. The Metascape web server was used to perform functional enrichment based on GO biological processes, hallmark gene sets, and KEGG pathways.

### Cell culture and reagents

Typical human pancreatic cancer cell lines, PANC-1, MIAPaCa-2, and BXPC-3, were obtained from Procell Life Science and Technology (Wuhan, China) via certified short tandem repeat (STR) profiling. BXPC-3 cells were cultured in RPMI-1640 medium, while PANC-1 and MIAPaCa-2 cells were cultured in DMEM supplemented with 10% fetal bovine serum (FBS) (VivaCell, Shanghai, China) and 1% streptomycin/penicillin (Beyotime, China) at 37 °C and 5% CO_2_. RSL3 (GC12431) and erastin (GC16630) were obtained from GlpBio (California, USA), while gemcitabine (HY-17026), ferrostatin-1 (HY-100579), torin1 (HY-13003), rapamycin (HY-10219), and AZD8055 (HY-10422) were obtained from MedChemExpress (MCE) (State of New Jersey, USA).

### Cell infection

To establish constitutive expression, a lentivirus carrying the HMGA2 protein coding region (Gene ID: 8091) or sgRNA targeting HMGA2 was acquired from GeneChem Biotechnology (Shanghai, China). To achieve constitutive effects, puromycin selection was conducted to obtain stable cell lines. The fused FLAG-HMGA2 overexpression lentiviral vector was constructed by Miaolin Biology (Wuhan, China) and transfected into MIAPaCa-2 cells for ChIP experiments.

### Cell viability, cell death, and migration assays

To assess cell death, cells were seeded on microscope cover glasses and treated as indicated. Subsequently, the cells were washed twice with phosphate-buffered saline (PBS) and stained with propidium iodide (PI) (Roche). The sections were studied using an inverted fluorescence microscope (TCS SP8, Leica). Cell growth was determined using a Cell Counting Kit-8 (CCK-8) and colony formation assays. In the CCK-8 assay, each type of cell (PANC-1 and MIAPaca-2 at 1500 cells/well, BXPC-3 at 2000 cells/well) was seeded into a 96-well plate with at least three replicates for different culture times. CCK-8 solution was added, and the absorbance at OD450 was measured using an iMark microplate absorbance reader (Bio-Rad, California, USA). For colony formation assays, each cell type (PANC-1 and MIAPaCa-2 at 1000 cells/well, BXPC-3 at 2000 cells/well) was seeded into six-well plates and cultured for 15–20 days. Subsequently, the cells were stained with 0.1% crystal violet to quantify positive colonies. Transwell assays (NEST Biotechnology, Wuxi, China) were used to assess cell migration. A total of 2 × 10^4^ cells were seeded in the upper chamber with FBS-free medium, while medium containing 10% FBS was added to the lower chamber. After 48 h of culture, the cells in the lower chamber were fixed with 4% PFA and stained with crystal violet.

### Cellular ROS, lipid peroxidation, and GSH detection

The cellular ROS levels were assessed by staining with 2,7-dichlorofluorescin diacetate (DCFH) (Merch, Darmstadt, Germany) for 30 min at 37 °C and then analyzed using a flow cytometer. For the analysis of lipid peroxidation, cells were stained with BODIPY-C11 (Invitrogen) or evaluated by measuring malondialdehyde (MDA) levels using a lipid peroxidation MDA assay kit (Beyotime, China) following the manufacturer’s instructions. Glutathione (GSH) levels were detected according to the standard protocol of the GSH and GSSG Assay Kit (#S0053, Beyotime, China).

### Real-time quantitative polymerase chain reaction (RT‒qPCR)

Total RNA extraction was performed using TRIzol reagent (Thermo Fisher), and cDNAs were synthesized using a reverse transcription kit (CW2569M, CWBIO, China). RT‒qPCR was performed using SYBR Green mix (Roche, USA). Gene expression was normalized to that of β-actin and calculated using the 2^–△△Ct^ method. The data are presented as the means ± SDs of three biological replicates. The primers used are listed in the Supplemental Table.

### Western blotting and antibodies

Cells were lysed using standard RIPA strong buffer supplemented with 1% PMSF and a proteinase cocktail (Solarbio, China). The protein concentration was determined using a BCA protein assay kit (GK10009, GLPBio). Equal amounts of protein were loaded onto SDS‒PAGE gels, transferred to PVDF membranes, and blocked with 5% skim milk. Specific primary antibodies were applied at 4 °C overnight. A chemiluminescence system (Tanon 5200, Tanon, China) was used for exposure. The following antibodies were used: β-actin (AC038, 1:10,000), P70S6 kinase 1 (A2190, 1:1000), phospho-p70 S6 kinase-T389 (AP1389, 1:1000), HRP goat anti-rabbit IgG (AS014, 1:5000), and HRP goat anti-mouse IgG (AS003, 1:5000) from ABclonal Technology (Wuhan, China); GPX4 (ab231174, 1:1000) and FACL4 (ab155282, 1:1000) from Abcam (Shanghai, China); HMGA2 (#61041, 1:1000) from Active Motif (California, USA); and FLAG (#14793, 1:2000) from Cell Signaling Technology (Shanghai, China).

### Pancreatic cancer tissue chip and immunohistochemical staining

Pancreatic cancer tissue chips were purchased from Shanghai Outdo Biotech Co., Ltd (cat.: OD-CT-DgPan 01-007). Informed consent was obtained from all subjects, and a total of 81 pairs of patients with pancreatic cancer on the chips were studied. Tissues were dewaxed and subjected to peroxidase blocking and antigen retrieval. Then, primary antibodies against GPX4 (ab231174, 1:400) from Abcam (Shanghai, China), HMGA2 (#61041, 1:200) from Active Motif (California, USA), SMA (M0851, 1:2000) from DAKO (Beijing, China), Vimentin (#5741, 1:500) and diaminobenzidine (DAB) were used for staining. Immunohistochemical staining was evaluated by experienced pathologists in a double-blinded manner. Image-Pro Plus (IPP) was used to analyze the staining intensity. The staining intensity was quantified using the integral optical density (IOD).

### Chromatin immunoprecipitation (ChIP) and quantitative PCR

ChIP experiments were conducted as previously described [22]. Briefly, 2 × 10^6^ cells were crosslinked with 1% formaldehyde for 10 min at room temperature. Following cell lysis, the cells were sonicated by a Bioruptor (Diagenode) for 30 min to obtain fragmented DNA. ChIP was incubated with the appropriate antibodies against mono-methyl-histone H3 (Lys4) (H3K4me1, #5326), acetyl-histone H3 (Lys27) (H3K27ac, #8173), and FLAG (#14793) from Cell Signaling Technology (Shanghai, China), followed by a pull-down assay using protein A/G-conjugated beads. The collected DNA was then used for quantitative PCR (qPCR) analysis. The primer sets used are listed in the Supplementary Table.

### RNA sequencing

Total RNA was extracted using TRIzol reagent (Invitrogen, Germany) and sequenced at CapitalBio Technology (Beijing, China) as described previously [23]. Independent triplicate samples were used for sequencing. Data analysis was conducted using ClusterProfiler R and KOBAS 3.0 software. Pathway enrichment analysis of DEGs was performed through the Kyoto Encyclopedia of Genes and Genomes (KEGG) database. The RNA sequencing data were submitted to the National Genomics Data Center of China (NGDC) under accession number PRJCA021355.

### In vivo xenograft assay

Four- to six-week-old NOD-SCID mice (strain no. T001492) (weighing 20–25 g) were obtained from Jiangsu GemPharmatech Co., Ltd (Nanjing, China) for in vivo xenograft experiments. Cultured PANC-1 sgNC or sgHMGA2 cells (3 × 10^6^) were subcutaneously transplanted into the right flank of all mice via randomization to experimental groups. The tumor volume was measured every 2 days, starting 5 days post-transplantation, and calculated using the following formula: tumor volume = (long × wide^2^) × 1/2. At the end of the experiment, the tumors were imaged and weighed after the mice were euthanized. A total of seven mice were used in each group, and no blinding methods were used. All animal procedures were performed according to protocols approved by the Animal Care Committee of Ningbo University.

### Luciferase reporter assay

The 128 bp long core promoter region or the 484 bp long proximal enhancer region was cloned and inserted into the luciferase reporter vector pGL4.10. The clone primer sets used are listed in the Supplementary Table.

Luciferase reporter analysis was performed according to the manufacturer’s protocol (Promega). Briefly, the constructed reporter vector was transfected into MCOK- or HMGA2-overexpressing PANC-1 cells via GP-transfect-Mate from Shanghai GenePhama Biotechnology (Shanghai, China). At 48 h post transfection, the cells were lysed for luciferase measurement, and firefly luciferase activities were normalized to Renilla luciferase activities.

### Statistical analysis

For the analysis of publicly available transcriptomic data, the Wilcoxon test was performed. The Pearson method was used to evaluate correlations. The graphical results are presented as the mean ± standard deviation (SD) of three independent replicates, and Student’s *t*-test was performed for the statistical analysis using GraphPad Prism 8.3 software. A *p*-value < 0.05 was considered to indicate statistical significance. * indicates *p* < 0.05, ** indicates *p* < 0.01.

## Results

### HMGA2 is highly expressed in pancreatic cancer and promotes cancer cell malignancy

**Fig. 1:**
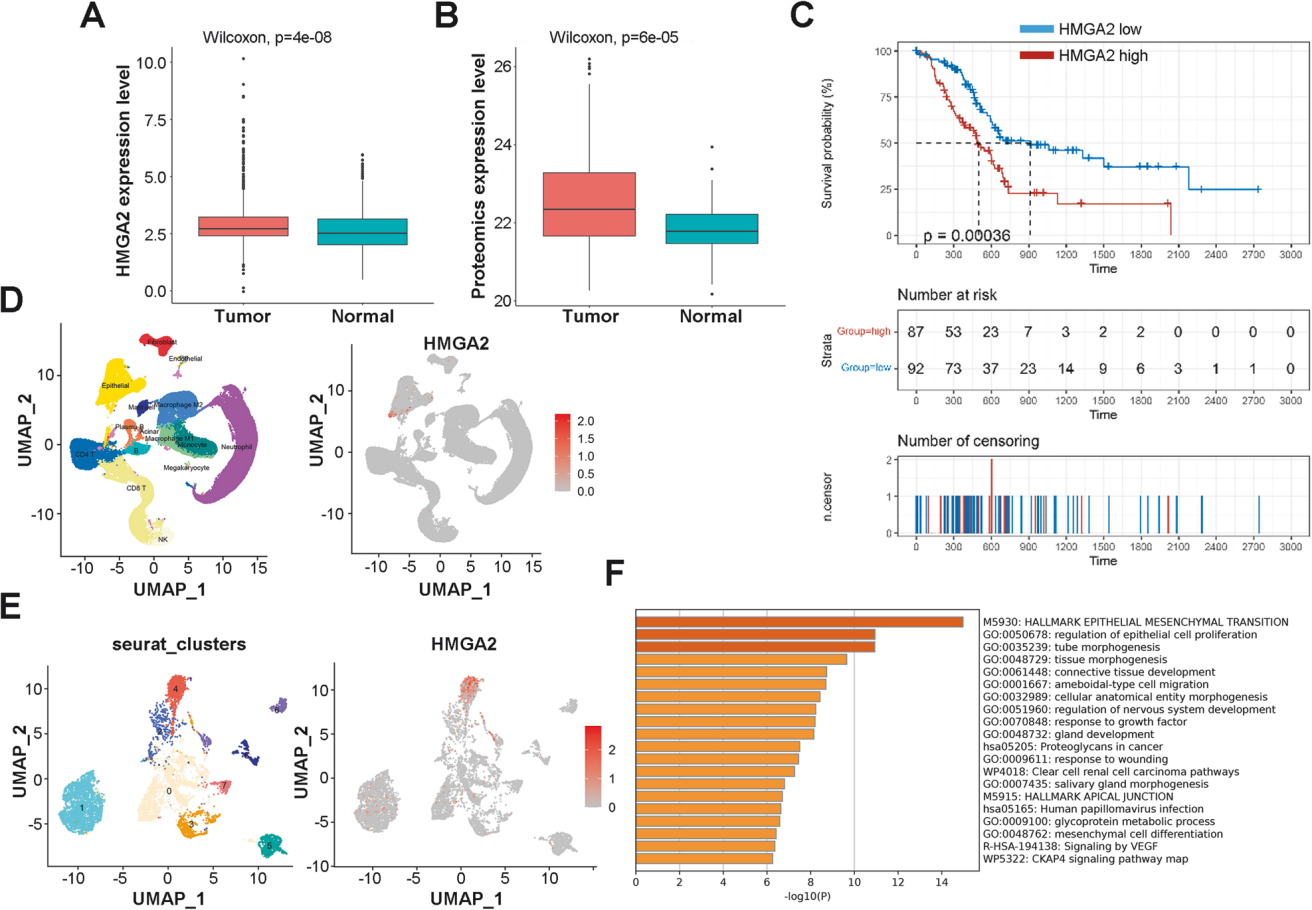
Features of HMGA2 expression in pancreatic cancer. **A** The transcriptome expression of HMGA2 in RNA-seq datasets from 869 pancreatic cancer tissues and 417 adjacent normal tissues. **B** Proteomic expression of HMGA2 in PDAC samples from CPTAC patients. **C** The survival probability of patients with pancreatic cancer with high or low HMGA2 expression is shown as the median expression value of HMGA2. The data were collected from the TCGA database. **D** The UMAP plot shows the categorization of single cells from human pancreatic cancer cells into different cell types (left) and the single-cell expression levels of HMGA2 (right). **E** Reclustering of epithelial cells from **D** revealed that HMGA2 was highly expressed in the epithelial cluster 4 subtype. **F** Functional enrichment analysis of the marker genes of epithelial cluster 4 (log2FC > 3 and *p* value adjusted < 0.01) via the Metascape web server.

To understand the significance of HMGA2, we compared the expression of HMGA2 between pancreatic cancer and normal tissues using publicly available RNA sequence data and proteomic data. Compared with those in normal tissues, significantly greater HMGA2 transcription (*p* = 4e-8) and protein expression (*p* = 6e-5) were detected in tumor tissues (Fig. 1A, B). Moreover, the HMGA2 expression level was negatively correlated with survival (*p* = 0.00036) (Fig. 1C). Our results were also supported by a published reference [31] showing that HMGA2 was significantly upregulated in pancreatic carcinoma (log_2_FC = 2.99, adjusted *p* value = 9.81e-57), and the constructed prognostic model showed that HMGA2 was strongly correlated with survival (hazard ratio = 1.53, Bonferroni correction *p* = 5.47e-07). In addition, high HMGA2 expression was positively associated with the expression of the proliferation markers Ki67 and PCNA (Fig. S1A, B) and the invasion and metastasis markers MMP9, SNAIL, and TWIST1 (Fig. S1C–E). Furthermore, we revealed a unique feature of HMGA2 expression in pancreatic cancer based on single-cell RNA sequencing data obtained from the publicly available GSE155698 dataset [28]. As indicated in Fig. 1D, HMGA2 was almost entirely expressed in epithelial cells among the 15 subtypes. Epithelial cells were divided into 9 clusters and HMGA2 was expressed mainly in cluster 4 (Fig. 1E). DEG analysis revealed 921 highly expressed genes in cluster 4 (Fig. S1F), and functional enrichment of the top DEGs (log_2_FC > 3, *n* = 81) indicated that EMT- and cell proliferation-related pathways were activated significantly (Fig. 1F). These results indicated the significance of HMGA2 in pancreatic cancer.

**Fig. 2:**
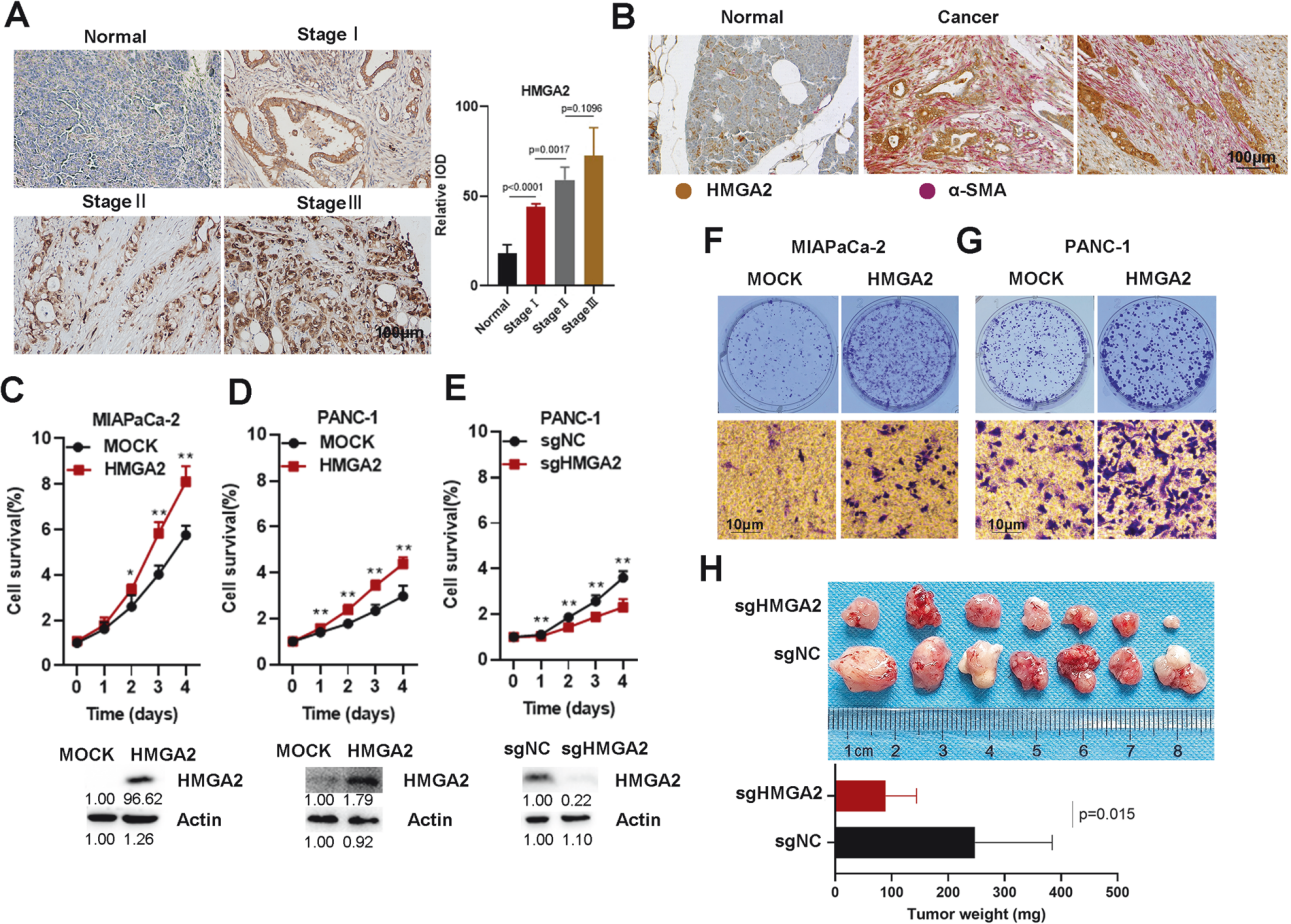
HMGA2 is highly expressed in pancreatic cancer and promotes cancer cell malignancy. **A** Representative images of IHC staining showing HMGA2 expression in pancreatic cancer tissue. The graph bar represents the results of the quantitative analysis. The intensity of HMGA2 staining was quantified using the integral optical density (IOD). The scale bar indicates 100 µm. **B** Representative images of HMGA2 and αSMA IHC double-stained pancreatic cancer tissue. Cell growth detection after HMGA2 overexpression in MIAPaCa-2 (**C**) and PANC-1 cells (**D**) or after HMGA2 deletion in PANC-1 cells (**E**). Cell viability was assessed using a Cell Counting Kit-8 (CCK-8) (*n* = 4). The efficiency of overexpression or deletion was confirmed using Western blotting, as indicated. The band intensity was quantified by ImageJ software. Evaluation of the effect of HMGA2 overexpression on the clonal growth and migration of MIAPaCa-2 (**F**) and PANC-1 cells (**G**). **H** Xenograft tumor model investigating the role of PANC-1 sgNC and PANC-1 sgHMGA2 in SCID mice. The graph bar represents the average tumor weight. The data are presented as the means ± SDs for each group of mice (*n* = 7). * indicates *p* < 0.05, ** indicates *p* < 0.01.

We confirmed significantly high expression of HMGA2 in patients with pancreatic cancer. A more advanced tumor stage was associated with higher HMGA2 levels according to immunohistochemical (IHC) staining of a total of 63 PAAD samples from the pancreatic cancer tissue chip (Fig. 2A). An obvious positive correlation between HMGA2 and Vimentin expression was detected (Fig. S2A). We also performed double IHC staining for HMGA2 and αSMA, a marker of cancer-associated fibroblasts. Both proteins were highly expressed in cancer tissue, with αSMA signals in the connective tissue and HMGA2 located within cancer cells (Fig. 2B). To further elucidate the function of HMGA2, we modulated its expression in widely used pancreatic cancer cell lines. The overexpression of HMGA2 in MIAPaCa-2 and PANC-1 cells promoted cell growth (Fig. 2C, D), while HMGA2 deletion inhibited cell growth in PANC-1 (Fig. 1E) and BXPC-3 cells (Fig. S2B). Furthermore, we observed that HMGA2 overexpression increased clonal growth and cell migration in MIAPaCa-2 and PANC-1 cells (Fig. 2F, G), while HMGA2 deletion inhibited clonal growth in PANC-1 cells (Fig. S2C). Importantly, slower growth (Fig. S2D) and smaller tumor sizes (Fig. 2H) were observed in HMGA2-deleted cells than in control vector-treated cells after subcutaneous implantation into SCID mice. These results suggested that HMGA2 promoted cancer cell malignancy.

### HMGA2 is correlated with ferroptosis signaling and influences the cellular redox status

**Fig. 3:**
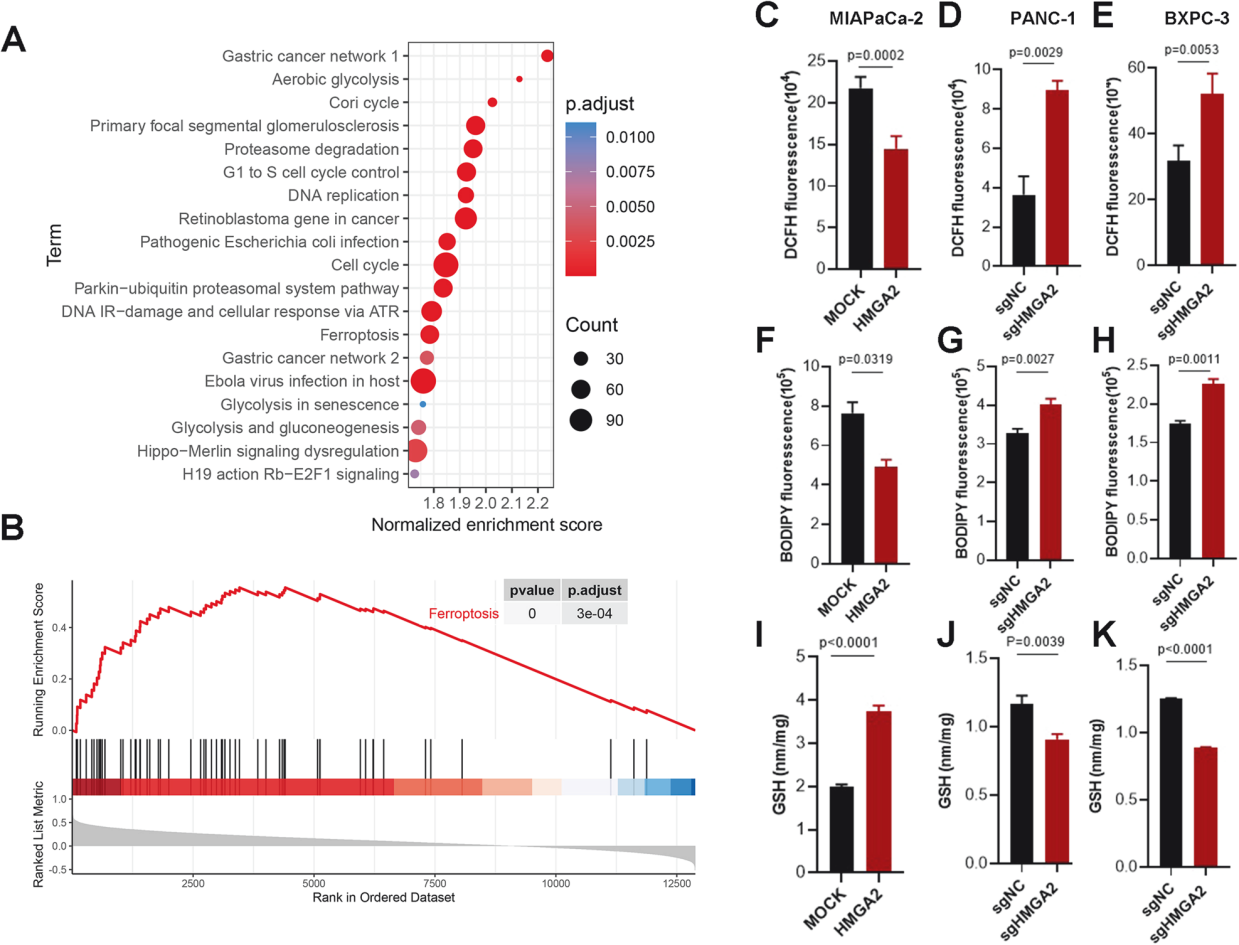
HMGA2 is correlated with ferroptosis signaling and influences the cellular redox status. Cell signaling pathway analysis (**A**) and gene set enrichment analysis (GSEA) (**B**) based on HMGA2 expression levels. Datasets from TCGA and GTEx were collected and divided into two groups, HMGA2-low and HMGA2-high, based on the median expression value of HMGA2 to collect DEGs for functional pathway analysis. Evaluation of cellular ROS (**C**), lipid peroxidation (**F**), and GSH levels (**I**) in MIAPaCa-2 cells overexpressing HMGA2. Evaluation of cellular ROS (**D**, **E**), lipid peroxidation (**G**, **H**), and GSH levels (**J**, **K**) in HMGA2-depleted PANC-1 and BxPC3 cells (*n* = 3). Cellular ROS were assessed using DCFH staining, and lipid peroxidation was assessed using BODIPY™ 581/591 C11 staining followed by flocytometry analysis. GSH was assessed using GSH and GSSG Assay Kits. The p value was determined by Student’s t test and is indicated in the figure.

To understand how HMGA2 affects cellular status, we analyzed datasets from TCGA and GTEx (https://www.gtexportal.org/home/index.html) and divided them into two groups, HMGA2-low and HMGA2-high, using the median expression value of HMGA2 to collect DEGs for functional pathway analysis. In addition to known regulatory pathways such as the cell cycle, DNA replication, and retinoblastoma genes in cancer, HMGA2 was significantly correlated with ferroptosis (Fig. 3A). Furthermore, gene set enrichment analysis (GSEA) of the TCGA database indicated that high expression of HMGA2 was strongly associated with the ferroptosis signaling pathway (*p* = 0) (Fig. 3B). Ferroptosis results from lipid ROS accumulation due to an imbalance in the cellular redox status. As indicated, a significant decrease in cellular ROS (Fig. 3C) and lipid peroxidation (Fig. 3F) occurred, along with an increase in GSH (Fig. 3I) in MIAPaCa-2 cells overexpressing HMGA2. Conversely, increased cellular ROS (Fig. 3D, E) and lipid peroxidation (Fig. 3G, H) and decreased GSH (Fig. 3J, K) were detected in both PANC-1 and BXPC-3 cells with HMGA2 deletion. Thus, our results suggested that HMGA2 is important for regulating cellular ferroptosis.

### HMGA2 confers resistance to ferroptotic cell death

**Fig. 4:**
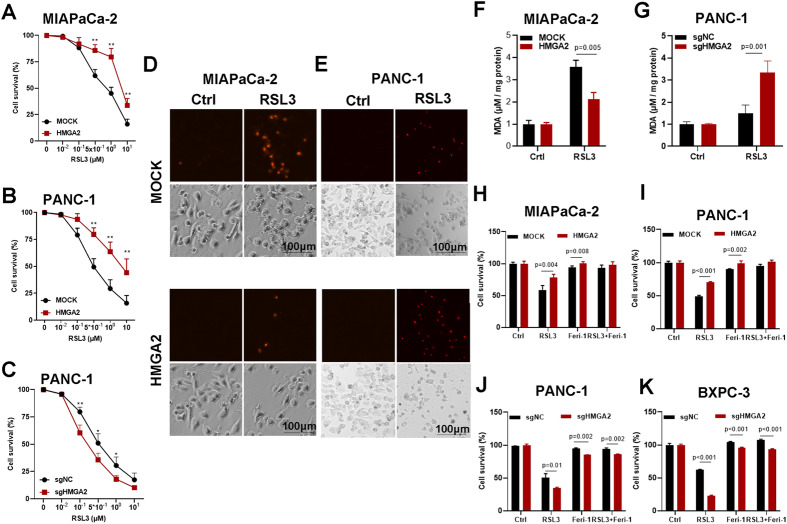
HMGA2 confers resistance to ferroptosis cell death. Cell viability analysis of the effects of HMGA2 alteration in MIAPaCa-2 (**A**) and PANC-1 (**B**) cells with HMGA2 overexpression and PANC-1 cells with HMGA2 deletion (**C**) after RSL3 treatment (*n* = 4). Representative images of cell death indicated by propidium iodide (PI) staining after HMGA2 overexpression in MIAPaCa-2 cells (**D**) and HMGA2 deletion in PANC-1 cells (**E**) treated with RSL3. The scale bar indicates 100 µm. Lipid peroxidation analysis of HMGA2-overexpressing MIAPaCa-2 cells (**F**) and HMGA2-deleted PANC-1 cells (**G**) after RSL3 treatment was performed using a Lipid Peroxidation MDA Assay Kit (*n* = 3). The evaluation of cell survival of cells with HMGA2 overexpression in MIAPaCa-2 (**H**) and PANC-1 cells (**I**), and HMGA2 deletion PANC-1 (**J**) and BXPC3 cells (**K**) treated with RSL3 and ferrostatin-1 (Ferr), a ferroptosis inhibitor (*n* = 4). The *p* value was determined using Student’s t test. * indicates *p* < 0.05; ** indicates *p* < 0.01.

The induction of ferroptotic cell death is an effective therapeutic approach for cancer treatment. Thus, ferroptotic cell death was evaluated in HMGA2-altered cancer cells using RSL3 and erastin, two widely accepted chemical inducers of ferroptosis. As indicated, cell survival was negatively correlated with the concentration of RSL3 (Fig. 4A, B) or erastin (Fig. S3A, B), and increased cell viability was observed in the HMGA2-overexpressing cells. Conversely, HMGA2 deletion promoted cell death in response to RSL3 treatment (Fig. 4C). Furthermore, fewer PI-positive signals were detected in the HMGA2-overexpressing cells (Fig. 4D), and more PI-positive signals were detected in the HMGA2-deleted cells (Fig. 4E), as shown by PI staining after RSL3 treatment. Moreover, cellular lipid peroxidation, indicated by the MDA level, was significantly decreased in HMGA2-overexpressing cells (Fig. 4F) and increased in HMGA2-deleted cells (Fig. 4G). Importantly, the difference in cell death between cells with HMGA2 overexpression (Fig. 4H, I) or deletion (Fig. 4J, K) and control cells was reversed after treatment with the ferroptosis-specific inhibitor ferrostatin-1. Our results strongly indicated that HMGA2 confers resistance to ferroptosis.

### HMGA2 enhances GPX4 transcription

**Fig. 5:**
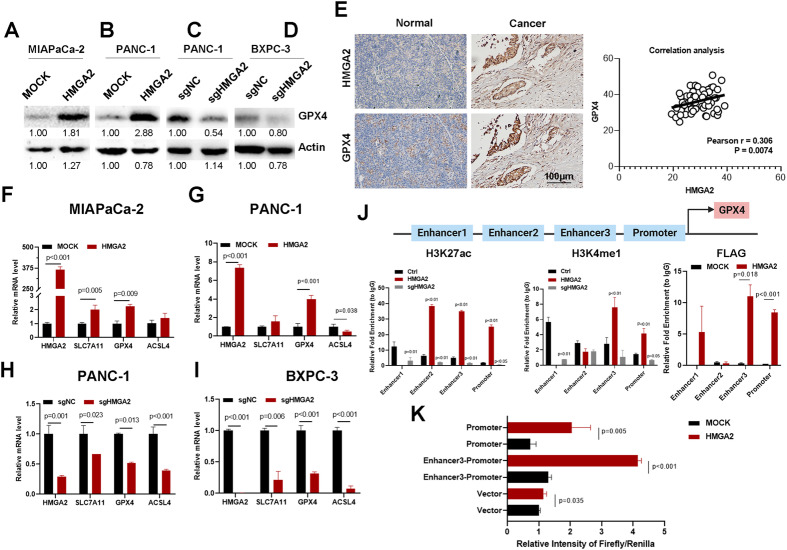
HMGA2 enhances GPX4 transcription. Detection of GPX4 protein expression in HMGA2-overexpressing MIAPaCa-2 (**A**) and PANC-1 cells (**B**) and HMGA2-depleted PANC-1 (**C**) and BXPC3 cells (**D**) using western blotting. The band intensity was quantified by ImageJ software. **E** Immunohistochemical staining analysis of HMGA2 and GPX4 expression in pancreatic cancer tissue. The scale bar indicates 100 µm. The correlation between HMGA2 and GPX4 expression was determined by statistical analysis. Detection of ferroptosis-related gene expression using RT‒qPCR in HMGA2-overexpressing MIAPaCa-2 (**F**) and PANC-1 cells (**G**) and HMGA2-deleted PANC-1 (**H**) and BXPC3 cells (**I**) (*n* = 3). **J** Enrichment analysis of the enhancer markers H3K4me1 and H3K27ac and of FLAG-HMGA2 fused to the cis-elements of the GPX4 promoter region using ChIP‒qPCR. **K** Luciferase activity assay demonstrating the transcriptional activation of GPX4 enhancer 3 and its promoter by HMGA2. The *p* value was determined by Student’s *t*-test.

GPX4 plays a vital role in adjusting cellular status and negatively regulating ferroptosis. We investigated whether GPX4 expression changed after altering HMGA2 levels. We confirmed that GPX4 protein expression increased in HMGA2-overexpressing cells (Fig. 5A, B) and decreased in HMGA2-deleted cells (Fig. 5C, D). Immunohistochemical staining of HMGA2 and GPX4 in 63 pancreatic cancer tissue chip samples revealed intense GPX4 staining in high HMGA2-expressing samples (Fig. 5E). Importantly, a significant positive correlation between HMGA2 and GPX4 was detected (*p* = 0.0074) after statistical analysis. We also observed that GPX4 mRNA was increased in HMGA2-overexpressing cells (Fig. 5F, G) and decreased in HMGA2-deleted cells (Fig. 5H, I). A significant correlation between HMGA2 and GPX4 was suggested based on TCGA data analysis (Fig. S4A). Other key genes related to ferroptosis, such as SLC7A11 and ACSL4, showed various degree of differences in HMGA2 overexpression or deletion status (Fig. S4B-D).

Interestingly, we detected three putative enhancer regions indicated by high levels of H3K27ac, a well-known enhancer marker, based on the UCSC genome browser for humans (GRCh38/hg38) (Fig. S4E). Therefore, we conducted a ChIP experiment to determine whether alterations in HMGA2 affect the histone modification status of enhancers, leading to changes in GPX4 transcription. As shown in Fig. 5J, significantly greater modifications of the enhancer markers H3K4me1 and H3K27ac were detected in the enhancer 3 and promoter regions of GPX4 in HMGA2-overexpressing PANC-1 cells than in control cells. Conversely, decreased levels of both enhancer markers were detected in HMGA2-deleted cells. However, although the H3K27ac level was altered by HMGA2 at the enhancer 2 position, there was no change in the H3K4me1 level. Moreover, no detectable signal was detected for either H3K4me1 or H3K27ac in HMGA2-overexpressing cells. The FLAG-HMGA2 fusion protein was introduced into and overexpressed in MIAPaCa-2 cells (Fig. S4F). A ChIP experiment was conducted with FLAG antibodies to determine the binding of HMGA2 to the DNA sequence. As indicated, HMGA2 significantly bound to the enhancer 3 and promoter regions (Fig. 5J). Through a luciferase reporter assay, we confirmed the transcriptional activation of HMGA2 on the enhancer 3 and promoter regions of the GPX4 gene (Fig. 5K). These results indicated that alterations in HMGA2 directly regulated histone modifications at the proximal promoter region to regulate GPX4 transcription.

### HMGA2 activates mTORC1 signaling

**Fig. 6:**
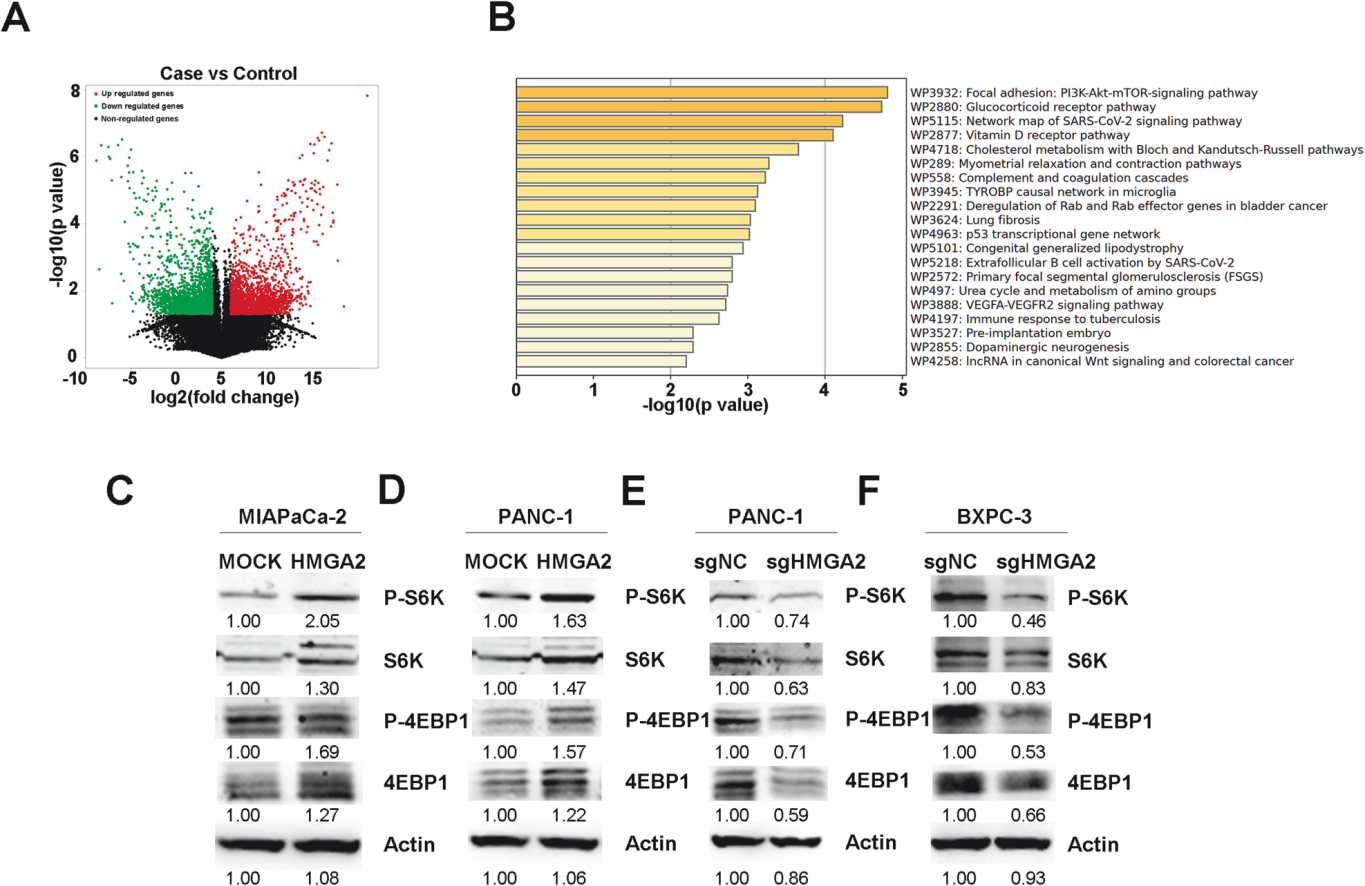
HMGA2 activates mTORC1 signaling. **A** Demonstration of alterations in gene expression based on RNA sequence in HMGA2-overexpressing MIAPaCa-2 cells compared to control cells. **B** The 20 most notably enriched KEGG pathways significantly changed the expression of genes identified from the RNA sequences after HMGA2 overexpression. Western blot detection of the mTORC1 downstream genes p70S6 kinase (S6K) and eukaryotic translation initiation factor 4E-binding protein 1 (4EBP1) and their phosphorylation status in HMGA2-overexpressing MIAPaCa-2 (**C**) and PANC-1 cells (**D**) and in HMGA2-deleted PANC-1 (**E**) and BXPC3 cells (**F**). The band intensity was quantified by ImageJ software.

To further understand why GPX4 upregulation occurred, we applied cycloheximide (CHX) to inhibit protein synthesis. A similar decrease in the rate of GPX4 degradation was observed between MOCK and HMGA2 cells, indicating that HMGA2 did not affect GPX4 protein degradation (Fig. S5A). We performed RNA sequencing to study the cellular effects comprehensively and identified the genes whose expression changed after HMGA2 overexpression. As shown in Fig. 6A, 1921 upregulated and 2015 downregulated genes were identified. KEGG analysis indicated that the PI3K-AKT-mTOR signaling pathway was the most significantly regulated pathway (Fig. 6B). Moreover, GPX4 mRNA translation is regulated by mTORC1 signaling [32], which is a key downstream mechanism in the KRAS pathway [33]. First, we confirmed that HMGA2 is a downstream target of KRAS using trametinib, an inhibitor of KRAS signaling (Fig. S5B). Subsequently, we evaluated the mTORC1 downstream genes p70S6 kinase (S6K) and eukaryotic translation initiation factor 4E-binding protein 1 (4EBP1). As indicated in Fig. 6C, D, increased phosphorylation of S6K and 4EBP1 was confirmed in HMGA2-overexpressing cells. Conversely, HMGA2-deleted cells showed decreased S6K and 4EBP1 phosphorylation, corresponding to downregulated GPX4 expression (Fig. 6E, F).

### HMGA2 promotes GPX4 protein synthesis through mTORC1 signaling

**Fig. 7:**
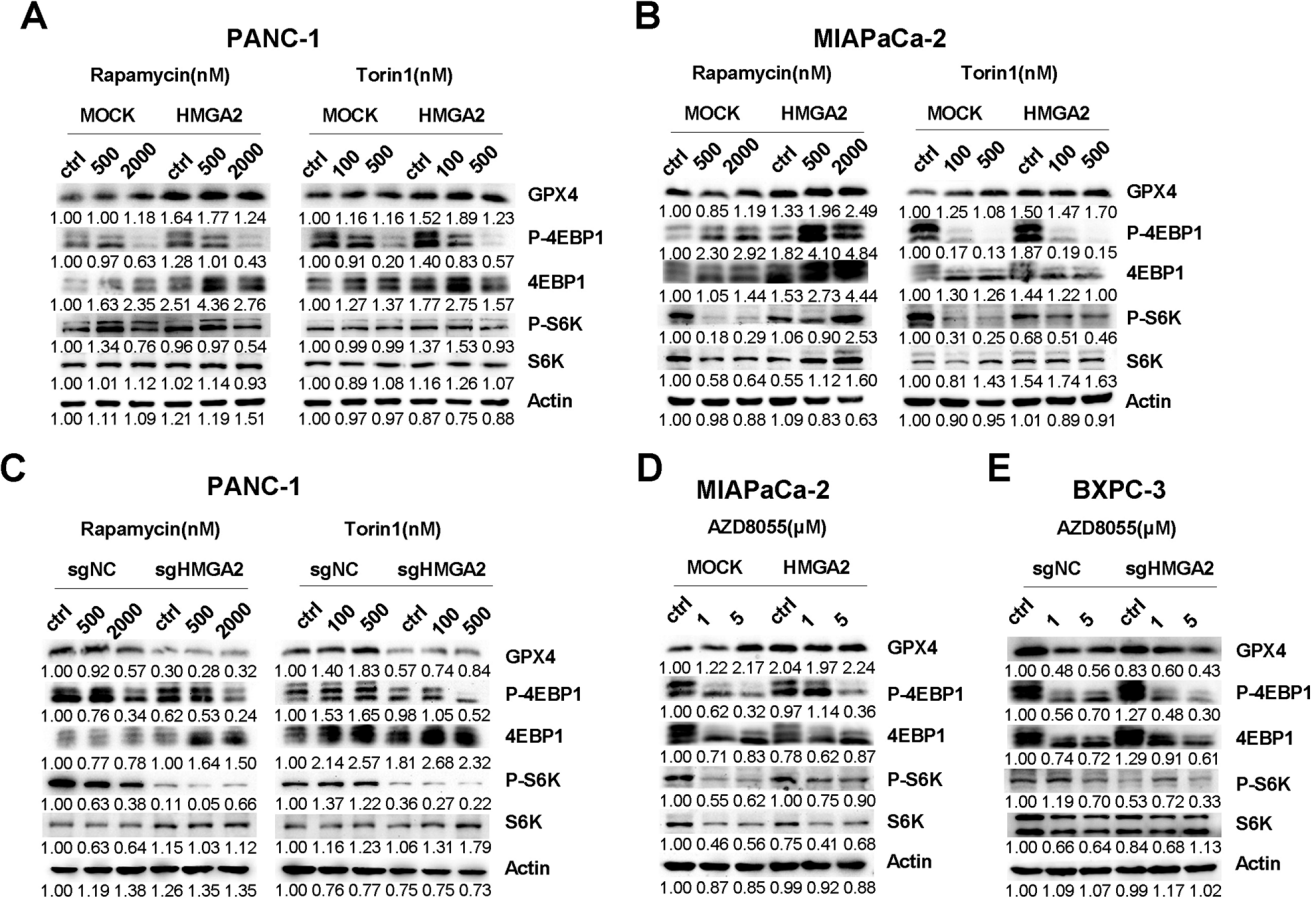
HMGA2 promotes GPX4 protein synthesis through mTORC1 signaling. Western blot detection of the mTORC1 downstream genes p70S6 kinase (S6K) and eukaryotic translation initiation factor 4E-binding protein 1 (4EBP1) and their phosphorylation status in PANC-1 (**A**) and MIAPaCa-2 (**B**) cells overexpressing HMGA2 and in PANC-1 cells with HMGA2 deletion (**C**) treated with the mTORC1 inhibitor rapamycin (Rap) and Torin1 for 2 h. Concentrations of rapamycin and Torin1 are indicated. Detection of S6K and 4EBP1 and their phosphorylation status in HMGA2-overexpressing MIAPaCa-2 (**D**) and HMGA2-deleted BXPC-3 (**E**) cells treated with AZD8055, an mTORC1 and mTPRC2 inhibitor. The band intensity was quantified by ImageJ software.

To further confirm whether HMGA2-altered mTORC1 signaling, we tested two widely used mTORC1 inhibitors, Torin1 and rapamycin. As shown in Fig. 7A, B, Torin1 and rapamycin inhibited the phosphorylation of both S6K and 4EBP1. Conversely, compared with MOCK cells, HMGA2-overexpressing cells exhibited significantly greater phosphorylation levels of both S6K and 4EBP1 (Fig. 7A, B). In HMGA2-deleted cells, the phosphorylation levels of both S6K and 4EBP1 were significantly lower than those in control cells (Fig. 7C). Furthermore, AZD8055, an inhibitor of both mTORC1 and mTORC2 complexes, was also applied. Similarly, compared with control cells, HMGA2-overexpressing cells exhibited significantly greater phosphorylation levels of both S6K and 4EBP1 (Fig. 7D), while HMGA2-deleted cells exhibited lower phosphorylation levels (Fig. 7E).

### HMGA2 alleviates sensitivity to combination treatment with a ferroptosis inducer and mTORC1 inhibition

**Fig. 8:**
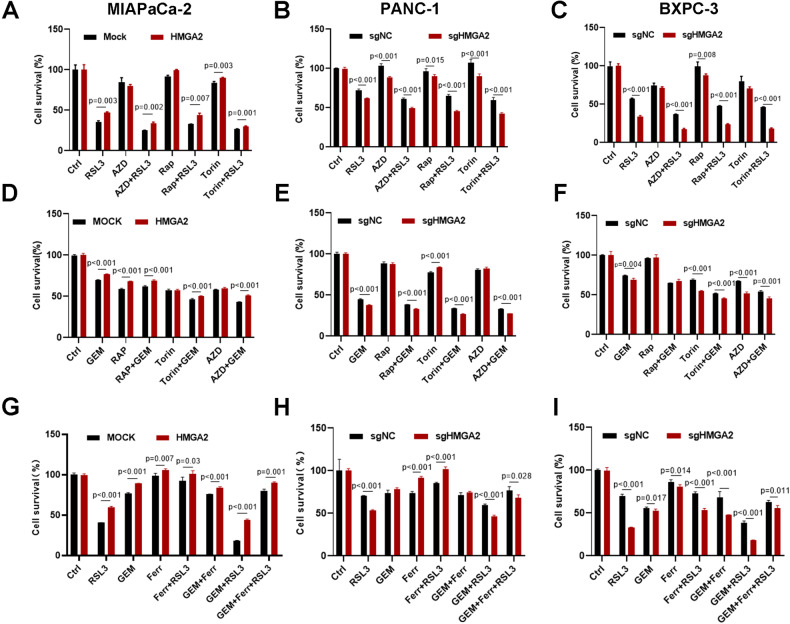
HMGA2 alleviates sensitivity to a combination treatment with a ferroptosis inducer and mTORC1 inhibition. Cell viability analysis of the effects of combination treatment with RSL3 and the mTORC1/2 inhibitors rapamycin (Rap), Torin1, and AZD8055 (AZD) in HMGA2-overexpressing MIAPaCa-2 (**A**), HMGA2-deleted PANC-1 (**B**) and BXPC-3 cells (**C**) (*n* = 4). Cell viability analysis of the effects of combination treatment with gemcitabine (GEM) and the mTORC1/2 inhibitors Rap, Torin1, and AZD on HMGA2-overexpressing MIAPaCa-2 (**D**) and HMGA2-deleted PANC-1 (**E**) and BXPC-3 cells (**F**) (*n* = 4). Cell viability analysis of the effects of the combination treatment of RSL3, GEM, and ferrostatin-1 (Ferr) and the mTORC1/2 inhibitors Rap, Torin1, and AZD on HMGA2 overexpression in MIAPaCa-2 (**G**) and HMGA2-deleted PANC-1 (**H**) and BXPC-3 cells (**I**) (*n* = 4). The p value was determined using a two-tailed unpaired Student’s *t*-test and is indicated.

Ferroptosis induction is an effective therapeutic approach for cancer treatment [34]. We determined whether HMGA2 affects sensitivity to mTORC1 inhibition combined with RSL3 treatment. As shown in Fig. 8A–C, a significant reduction in cell survival was observed after combination treatment with RSL3 and mTORC1 inhibitors such as rapamycin, AZD, or Torin1 compared to treatment with a single reagent alone. In contrast, HMGA2-overexpressing cells had significantly greater survival rates (Fig. 8A), while HMGA2-deleted cells had lower survival rates (Fig. 8B, C). Similar effects were observed with a combination of gemcitabine, a widely used first-line clinical drug for pancreatic cancer, and mTORC1 inhibitors (Fig. 8D–F).

We also assessed whether HMGA2 regulates the effects of combination treatment with gemcitabine and RSL3. As indicated, combination treatment with gemcitabine and RSL3 had a significant synergistic effect on cell survival. However, HMGA2 overexpression considerably reduced cell survival (Fig. 8G), while HMGA2 deletion increased cell sensitivity to treatment (Fig. 8H, I). Notably, cell survival was considerably restored by treatment with the ferroptosis inhibitor ferrostatin-1, emphasizing the importance of ferroptosis in pancreatic cancer treatment.

## Discussion

Pancreatic cancer is one of the most malignant cancers and is the leading cause of cancer-related death. The development of effective therapies for pancreatic cancer remains an enormous challenge. Although the use of neoadjuvant chemotherapy and/or radiotherapy has increased, it remains largely refractory to treatment [3]. This is particularly relevant given the physical barrier created by the tumor microenvironment and heterogeneity of cancer cell subtypes in pancreatic cancer, which can hinder the delivery of chemotherapy and lead to treatment failure [35]. Ferroptosis has emerged as an effective approach to cancer therapy, and it involves four main pathways: iron metabolism, GPX4 regulation, lipid metabolism, and a novel sex hormone-related pathway [36, 37]. Given the unique tumor microenvironment in pancreatic cancer and the distinct characteristics of ferroptosis, effective regulation of the ferroptosis pathway in pancreatic cancer may significantly improve the effectiveness of chemotherapy.

HMGA2 is frequently overexpressed in various cancer types and plays critical roles in tumor development, including promoting cell cycle progression, DNA repair, EMT, and chemoresistance, making it a pivotal regulator of cancer progression and a potential therapeutic target [18, 38]. In this study, we found that HMGA2 is highly expressed in pancreatic cancer and enhances resistance to cell death induced by chemotherapy. Given the unique tumor microenvironment in pancreatic cancer and the distinct characteristics of ferroptosis, effective regulation of the ferroptosis pathway in pancreatic cancer may significantly improve the effectiveness of chemotherapy.

Our results indicate that ferroptosis inducers, such as gemcitabine, are more effective in treating pancreatic cancer than are conventional chemotherapies. GPX4, a key regulator of lipid peroxidation and a central player in ferroptosis, is a potential target for modulating ferroptosis in cancer cells. In our study, we observed that HMGA2 overexpression significantly increased GPX4 expression, leading to increased cell survival under treatment with RSL3 or gemcitabine. This finding emphasizes the crucial link between ferroptosis and resistance to cell death. Recent research has revealed that rapamycin treatment decreases GPX4 protein levels, and high-concentration rapamycin treatment can potently inhibit 4EBP1 phosphorylation [32, 39], suggesting a potential interaction between ferroptosis and the mTOR pathway. In our study, we first demonstrated a positive correlation between HMGA2 and GPX4, where HMGA2 overexpression increased GPX4 levels in pancreatic cancer cells, reducing lipid peroxidation under the ferroptosis inducer RSL3.

HMGA2, an oncogene widely reexpressed in various cancers, regulates tumor cell functions through diverse mechanisms. It contains three AT-rich regions known as AT-hooks and functions as a transcription factor. HMGA2 also recruits histone acetyltransferases (HATs) to specific gene promoters to promote H3K27 and H3K9 acetylation [40]. Histone modifications are vital for regulating gene transcription efficiency, and H3K4 methylation and H3K27 acetylation are biomarkers of enhancer activity [41]. Increased levels of H3K4Me and H3K27Ac indicated increased GPX4 transcription in HMGA2-overexpressing cells. Furthermore, the direct binding of HMGA2 to the proximal promoter was confirmed. Thus, our findings indicate that HMGA2-altered cis-element modifications in the promoter region of the GPX4 gene, impacting gene transcription efficiency. Based on the prediction of transcription factors via the UCSC genome browser for humans (GRCh38/hg38), p65 and SP1 were identified as candidates within the GPX4 promoter region [42]. Whether p65 and SP1 form complexes with HMGA2 and whether HMGA2 alters the working status of the transcription factor complex and chromatin spatial conformation require further research.

The mTOR activation pathway is a crucial biosynthetic signal that regulates protein synthesis by phosphorylating downstream molecules such as the eukaryotic initiation factors 4EBP1 and S6K [43]. Herein, we demonstrated that HMGA2 can activate the mTOR signaling pathway by increasing the phosphorylation of downstream molecules. Importantly, the inhibitory effects of mTOR inhibitors are counteracted by HMGA2 overexpression. However, the precise mechanisms underlying the activation of the mTOR pathway by HMGA2 and specific downstream targets still need to be clarified. The mTORC1 pathway is complex and is responsible for various kinds of cellular stress and has various effects on cells. HMGA2 is closely related to RAS signaling, which acts as an upstream regulator of the PI3K-AKT-mTORC1, RAF-MEK, and NF-kB pathways [33]. RAS-ERK signaling can activate mTORC1, and the MEK inhibitor trametinib (GSK1120212), according to our present results, and U0126, according to a published paper [44], demonstrated that HMGA2 was a downstream target of RAS signaling. It has been reported that HMGA2 overexpression can induce the phosphorylation of AKT and mTOR [45]. A recent paper reported that HMGA2 can activate the transcription of IGF2BP, which in turn activates AKT-mTOR through the signal transduction of the IGF2-IGF1R interaction in sarcoma [46]. It was also reported that the HMGA protein can form a complex with NF-kB, which was reported to activate GPX4 transcription, to enhance target gene transcription through an enhanceosome model [38]. However, the precise target or partner of HMGA2 that directly activates mTORC1 signaling still needs to be clarified. Furthermore, a previous study indicated that HMGA1 can bind RNA and play a critical role in the pre-mRNA splicing of the presenilin-2 gene [47]. Therefore, it is also possible that HMGA2 has an ability similar to that of HMGA1 to bind to mRNA for the translational regulation of GPX4. One key point to consider is that when HMGA2 is deleted or mTOR inhibitors are applied, GPX4 protein expression is not entirely abolished. This finding suggested that other pathways may regulate the resistance of pancreatic cancer cells to ferroptosis, which warrants further exploration.

In summary, our study elucidates the mechanism by which HMGA2 enhances resistance to cell death in pancreatic cancer by inhibiting ferroptosis. This occurs through the activation of the mTOR signaling pathway and the consequent increase in GPX4 levels. These findings provide a potential target for future therapeutic approaches to overcome chemoresistance in pancreatic cancer.

### Supplementary information

Supplemental data

Original Data File

CDD reproducibility checklist

## Data Availability

Data are available according to the request to corresponding authors.
