# Supplementary material for: HMGA2 alleviates ferroptosis by promoting GPX4 expression in pancreatic cancer cells
Source: Cell Death Dis. 2024 Mar 16;15(3):220. doi: 10.1038/s41419-024-06592-y (PMC10944463; doi:10.1038/s41419-024-06592-y)
Supplement: Supplementary file 1 — Supplemental data [file 41419_2024_6592_MOESM1_ESM.pdf]

Supplementary data

Figure S1. Features of HMGA2 expression in pancreatic cancer

The transcriptome expression of the MKI67 (**A**), PCNA (**B**), SNAIL2 (**C**), MMP9 (**D**), TWIST1 (**E**) genes in RNA-seq datasets for 869 pancreatic cancer patients was compared between the HMGA2-high and HMGA2-low groups, based on the median expression of HMGA2. **F**. The volcano plot displays the differentially expressed genes (DEG_clster4_other.csv) in epithelial cell cluster 4 compared to other epithelial cell subpopulations.

Figure S2. HMGA2 is highly expressed in pancreatic cancer and promotes cancer cell malignancy.

A. Representative images of HMGA2 and Vimentin expression based on immunohistochemical staining of pancreatic cancer tissue. Scale bare indicates 100 μm. **B.** Cell growth detection after HMGA2 deletion in BXPC-3 cells. Cell viability analysis was accessed using CCK-8 (n = 4). The efficiency of deletion was confirmed using western blot as listed. **C.** Evaluation of HMGA2 deletion on the clonal growth of PANC-1 cell. **D.** Xenograft tumor model investigating the growth of PANC-1 sgNC and PANC-1 sgHMGA2 cells in SCID mice. The graph bar represents the average tumor volume. Data are represented as mean ± SD for each group of mice. (n = 7). $*p < 0.05$, $**p < 0.01$.

Figure S3. HMGA2 confers resistance to ferroptosis cell death

Cell viability analysis of the effects of HMGA2 alternation in MIAPaCa-2 (**A**) and PANC-1 (**B**) cells with HMGA2 overexpression after erastin treatment. Cell viability analysis was accessed using CKK-8 at A450 (n = 4). The p-value was determined using Student's t-test. $*p < 0.05$; $**p < 0.01$

Figure S4. HMGA2 enhances GPX4 transcription

Correlation between HMGA2 and GPX4 (**A**), SLC7A11 (**B**), and ACSL4 (**C**)mRNA expression using data from the TCGA database. **D**. Detection of SLC7A11 and ACSL4 protein levels based on Werstern blot in PANC-1 cells with HMGA2 overexpression. **E.** H3K27Ac status of the cis-

element in GPX4 promoter region from UCSC genome browser on humans (GRCh38/hg38). **F**. Confirm of fused FLAG-HMGA2 overexpression in MIAPaCa-2 cells by Werstern blot. The band intensity was quantified by ImageJ software.

Figure S5. HMGA2 promotes GPX4 protein synthesis through mTORC1 signaling

A. Detection of the GPX4 protein level in PANC-1 MOCK and HMGA2 cells after cycloheximide (CHX) treatment at different times. The correlation between GPX4 level and CHX time was analyzed using Image J. **B**. Western blotting analysis of the HMGA2 protein level in three pancreatic cell lines PANC-1, ASPC-1, and BXPC-3 cells treated with KRAS signaling inhibitor trametinib. The band intensity was quantified by ImageJ software.

Supplementary Table. Primers used in present study

	Name	Sequence (5'-3')
RT-qPCR	HMGA2 S	GCGCCTCAGAAGAGAGGAC
	HMGA2 AS	GGTCTCTTAGGAGAGGGCTCA
	GPX4 S	GATACGCTGAGTGTGGTTTG
	GPX4 AS	ATCTTCATCCACTTCCACAGC
	GSS S	GACCAGCGTGCCATAGAGAATGA
	GSS AS	CATGTGACCTCTCCAGCAGTAGAC
	ACSL4 S	TCTGCTTCTGCTGCCCAATT
	ACSL4 AS	CGCCTTCTTGCCAGTCTTTT
	SLC7A11 S	GTTGCGTCTCGAGAGGGTCA
	SLC7A11 AS	GTCGAGGTCTCCAGAGAAGAGC
ChIP qPCR	enhancer1 S	CCGTGTTTTCCCCACTTCTG
	enhancer1 AS	TTGCCGGTGTTGTTTTCCTT
	enhancer2 S	CAAACCATCCATGACGCCTC
	enhancer2 AS	ACCTTTTCTGACCCTGCACT
	enhancer3 S	TTTTCTGAGCACATACCGCG
	enhancer3 AS	GTCAGTGACTCCCCATCCTG
	promoter S	AGGAGTTCGAGATCAGCCTG
	promoter AS	CGATTCTCCTGACTCAGCCT
Clone primers	GPX4-pro-F	TACCTGAGCTCGCTAGCCAGGAGTTCGAGATCAGCCTG
	GPX4-enh3-F	TACCTGAGCTCGCTAGCCTTTTCTGAGCACATACCGCG
	GPX4-R	AGGCCAGATCTTGATATCCCGATTCTCCTGACTCAGCCT

Figure S1

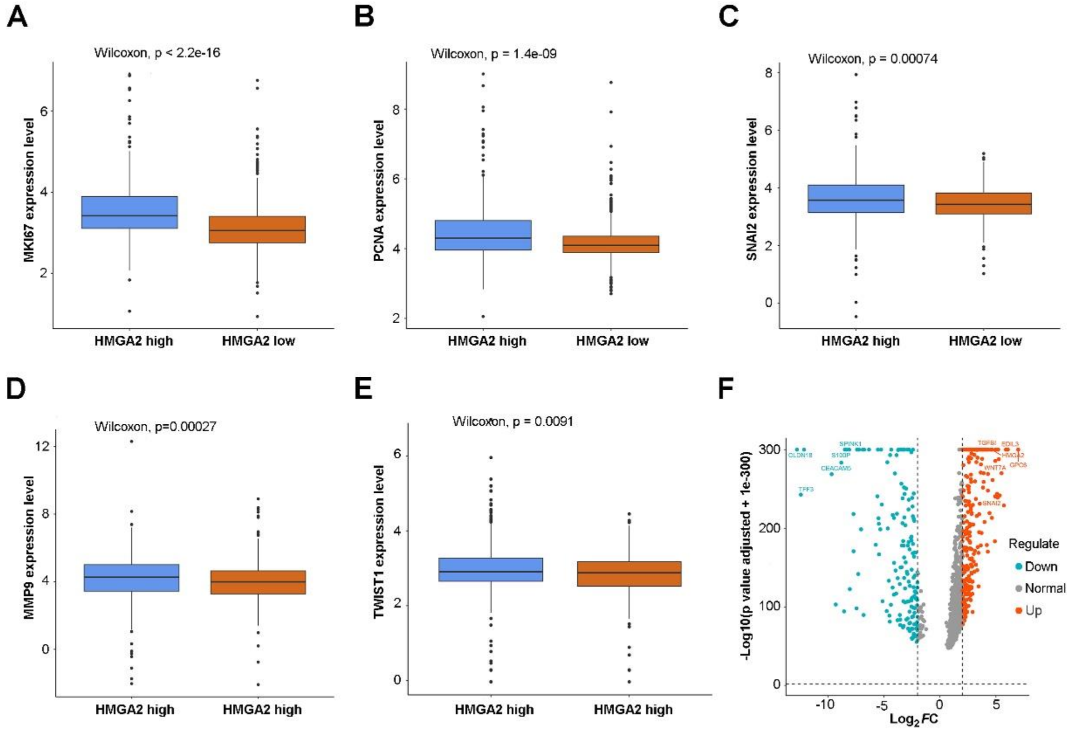

A
Wilcoxon, p < 2.2e-16
MK67 expression level
HMGA2 high
HMGA2 low
B
Wilcoxon, p = 1.4e-09
PCNA expression level
HMGA2 high
HMGA2 low
C
Wilcoxon, p = 0.00074
SNAI2 expression level
HMGA2 high
HMGA2 low
D
Wilcoxon, p=0.00027
MMP9 expression level
HMGA2 high
HMGA2 high
E
Wilcoxon, p = 0.0091
TWIST1 expression level
HMGA2 high
HMGA2 high
F
-Log10(p value adjusted + 1e-300)
Log_2FC
Regulate
Down
Normal
Up

Figure S2

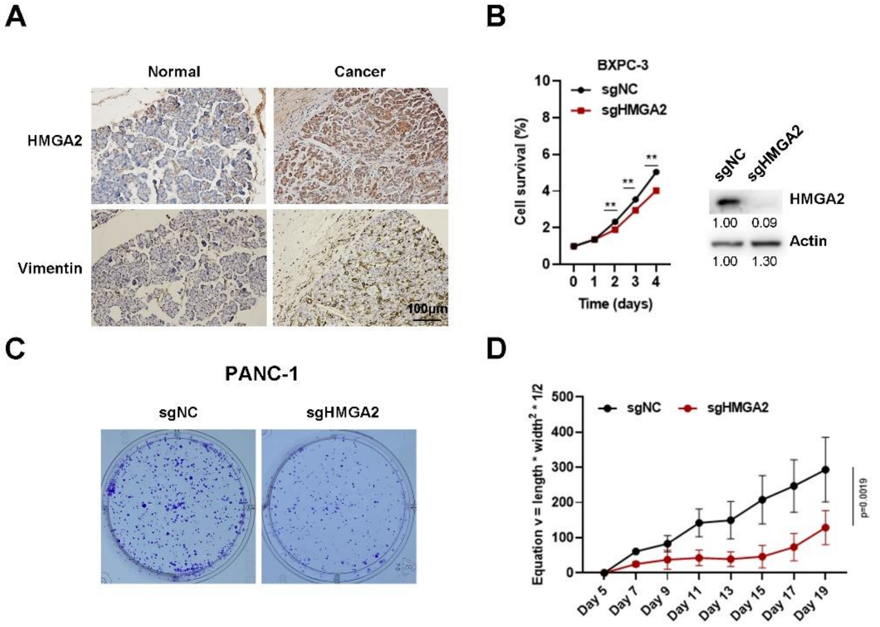
A
Normal
Cancer
HMGA2
Vimentin
100μm
B
BXPC-3
sgNC
sgHMGA2
Cell survival (%)
Time (days)
**
HMGA2
1.00 0.09
Actin
1.00 1.30
C
PANC-1
sgNC
sgHMGA2
D
sgNC
sgHMGA2
Equation v = length * width² * 1/2
Day 5
Day 7
Day 9
Day 11
Day 13
Day 15
Day 17
Day 19
p=0.0019

Figure S3

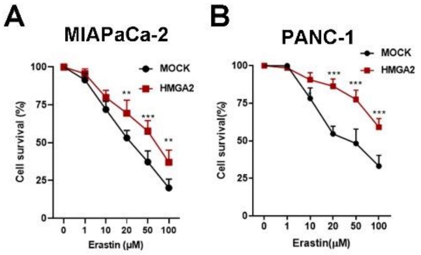
A
MIAPaCa-2
MOCK
HMGA2
Cell survival (%)
Erastin (μM)
B
PANC-1
MOCK
HMGA2
Cell survival(%)
Erastin(μM)

Figure S4

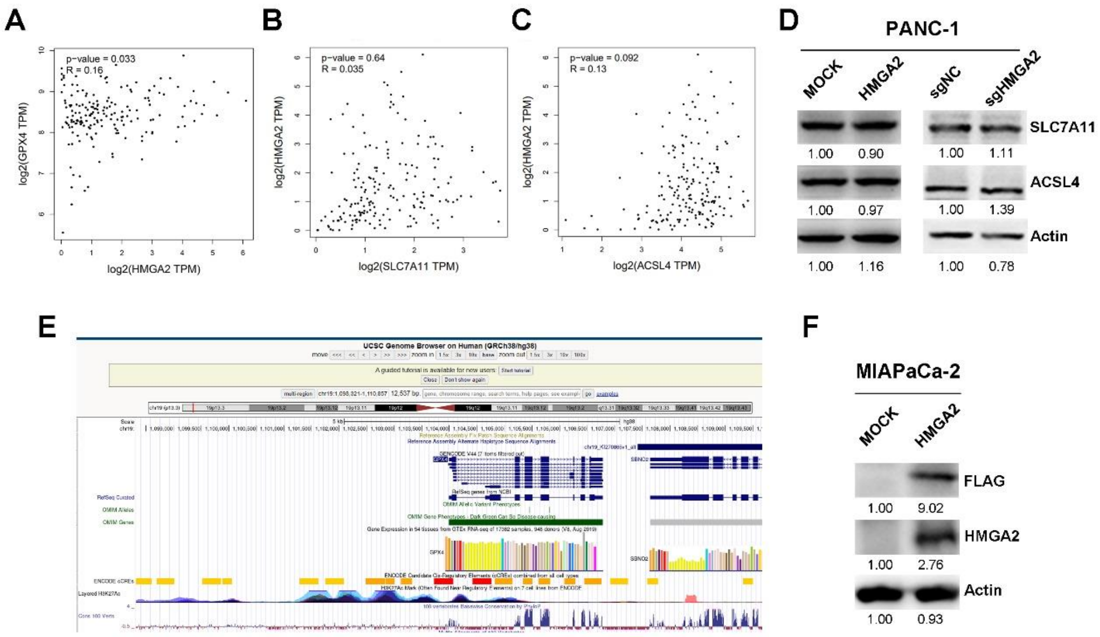
A
p−value = 0.033
R = 0.16
log2(GPX4 TPM)
log2(HMGA2 TPM)
B
p−value = 0.64
R = 0.035
log2(HMGA2 TPM)
log2(SLC7A11 TPM)
C
p−value = 0.092
R = 0.13
log2(HMGA2 TPM)
log2(ACSL4 TPM)
D
PANC-1
MOCK
HMGA2
sgNC
sgHMGA2
SLC7A11
1.00 0.90 1.00 1.11
ACSL4
1.00 0.97 1.00 1.39
Actin
1.00 1.16 1.00 0.78
E
UCSC Genome Browser on Human (GRCh38/hg38)
F
MIAPaCa-2
MOCK
HMGA2
FLAG
1.00 9.02
HMGA2
1.00 2.76
Actin
1.00 0.93

Figure S5

A

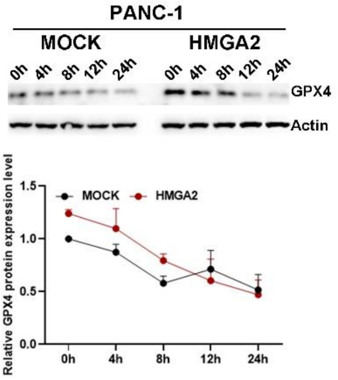
PANC-1
MOCK
HMGA2
0h
4h
8h
12h
24h
GPX4
Actin
Relative GPX4 protein expression level
MOCK
HMGA2
1.5
1.0
0.5
0.0
0h
4h
8h
12h
24h

B

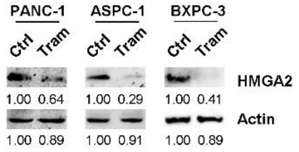
PANC-1
ASPC-1
BXPC-3
Ctrl
Tram
HMGA2
1.00 0.64
1.00 0.29
1.00 0.41
Actin
1.00 0.89
1.00 0.91
1.00 0.89
