# Supplementary material for: HMGA2 alleviates ferroptosis by promoting GPX4 expression in pancreatic cancer cells
Source: Cell Death Dis. 2024 Mar 16;15(3):220. doi: 10.1038/s41419-024-06592-y (PMC10944463; doi:10.1038/s41419-024-06592-y)
Supplement: Supplementary file 2 — Original Data File [file 41419_2024_6592_MOESM2_ESM.pdf]

Figure 2C

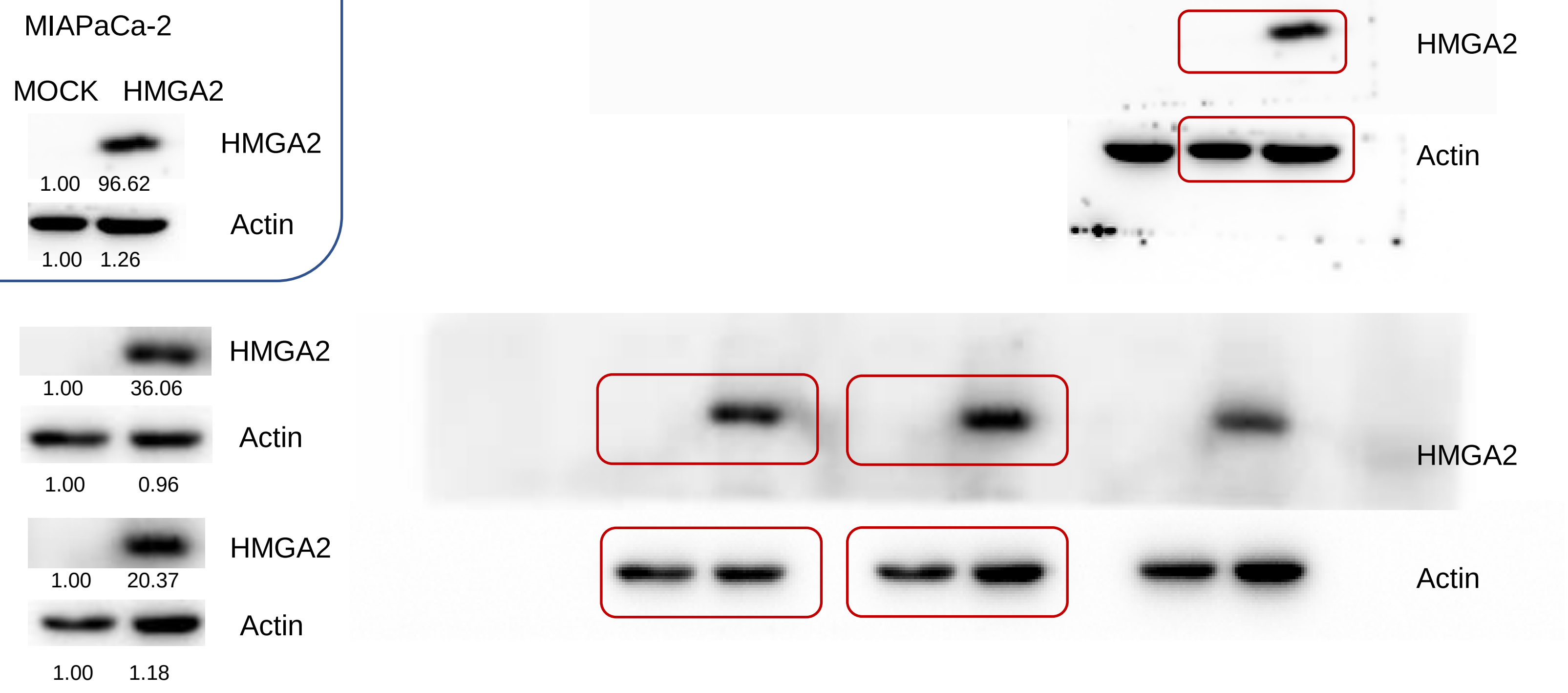
MIAPaCa-2
MOCK
HMGA2
HMGA2
1.00
96.62
Actin
1.00
1.26
HMGA2
1.00
36.06
Actin
1.00
0.96
HMGA2
1.00
20.37
Actin
1.00
1.18
HMGA2
Actin
HMGA2
Actin

Figure 2D

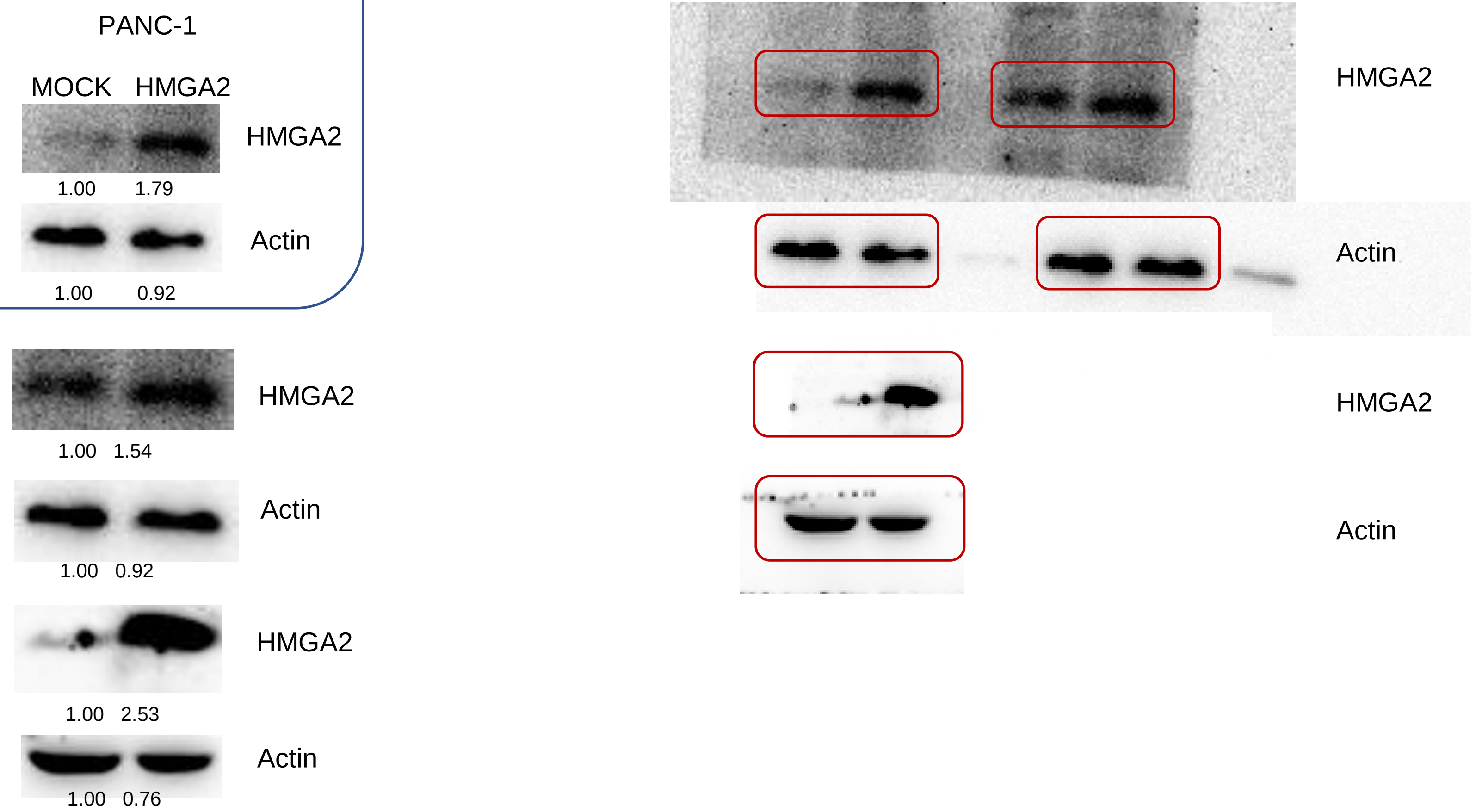

PANC-1
MOCK
HMGA2
HMGA2
1.00
1.79
Actin
1.00
0.92
HMGA2
1.00 1.54
Actin
1.00 0.92
HMGA2
1.00 2.53
Actin
1.00 0.76
HMGA2
Actin
HMGA2
Actin

Figure 2E

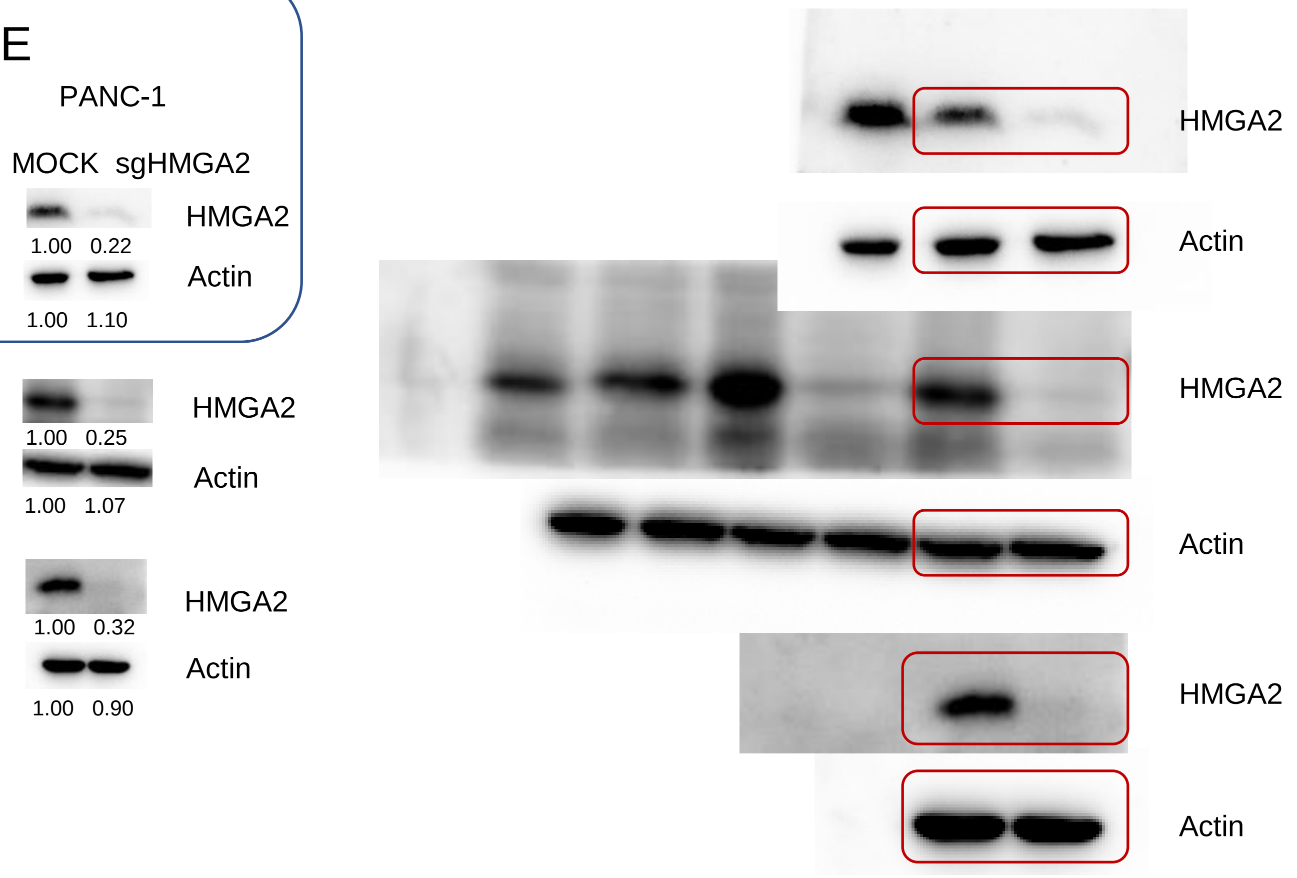
PANC-1
MOCK sgHMGA2
HMGA2
1.00 0.22
Actin
1.00 1.10
HMGA2
1.00 0.25
Actin
1.00 1.07
HMGA2
1.00 0.32
Actin
1.00 0.90
HMGA2
Actin
HMGA2
Actin
HMGA2
Actin

Figure 5A

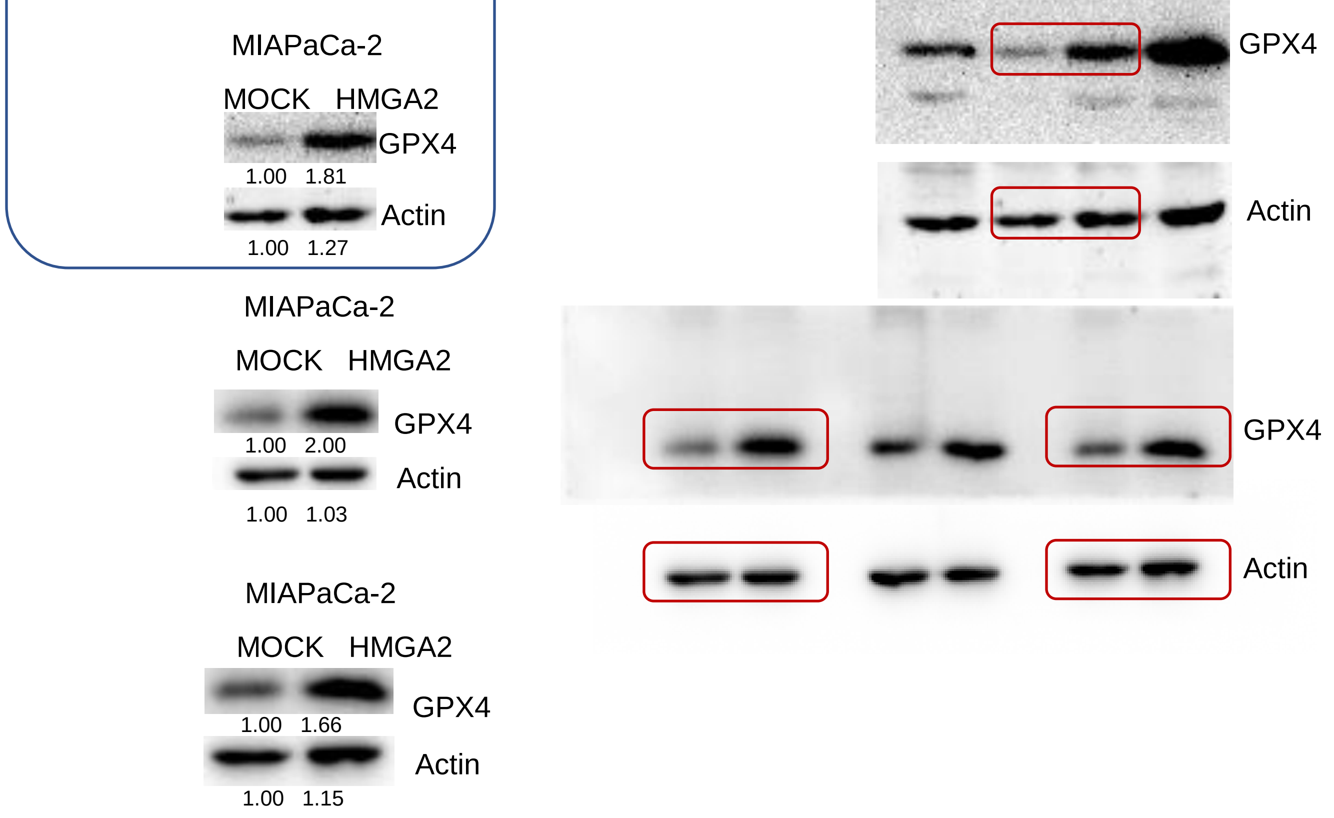
MIAPaCa-2
MOCK HMGA2
GPX4
1.00 1.81
Actin
1.00 1.27
MIAPaCa-2
MOCK HMGA2
GPX4
1.00 2.00
Actin
1.00 1.03
MIAPaCa-2
MOCK HMGA2
GPX4
1.00 1.66
Actin
1.00 1.15
GPX4
Actin
GPX4
Actin

Figure 5B

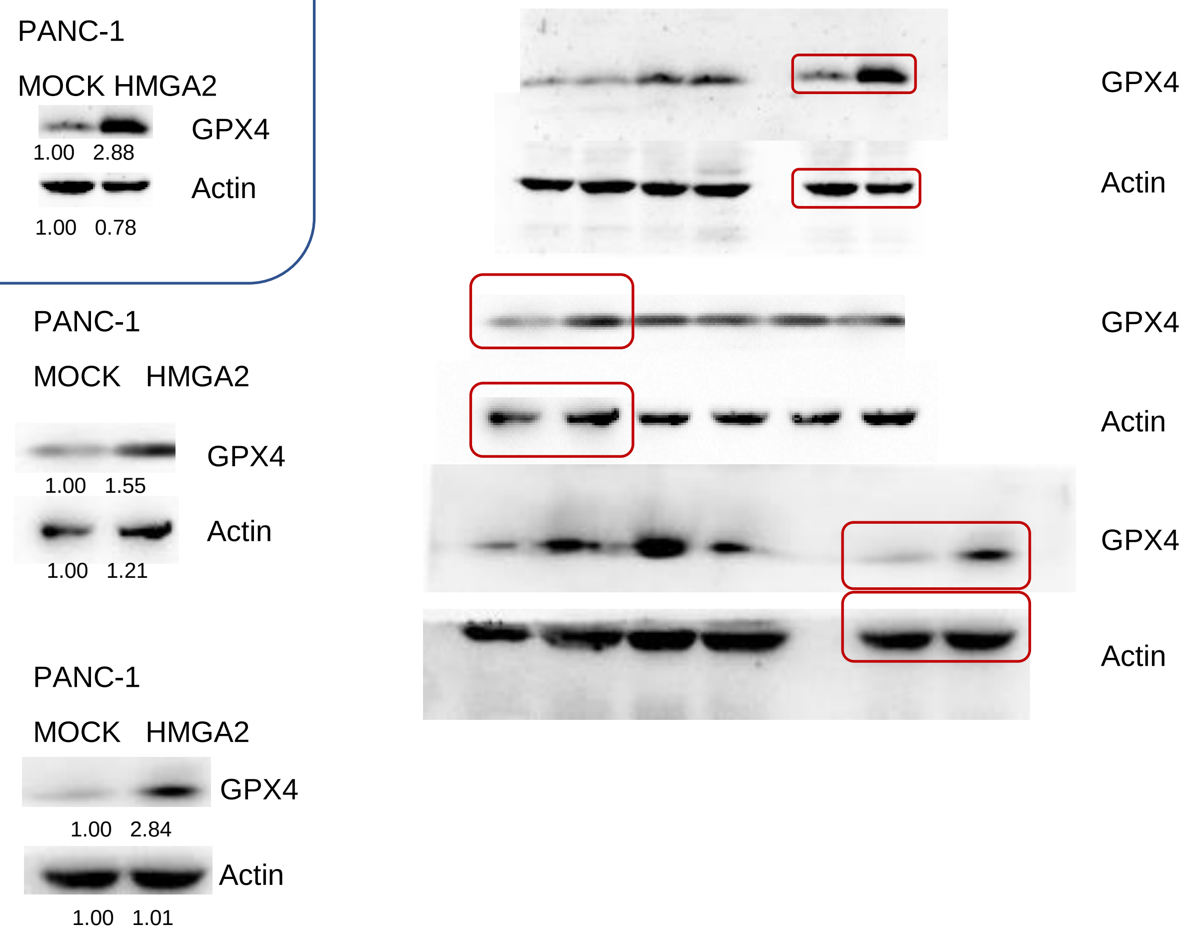
PANC-1
MOCK HMGA2
GPX4
1.00 2.88
Actin
1.00 0.78
PANC-1
MOCK HMGA2
GPX4
1.00 1.55
Actin
1.00 1.21
PANC-1
MOCK HMGA2
GPX4
1.00 2.84
Actin
1.00 1.01
GPX4
Actin
GPX4
Actin
GPX4
Actin

Figure 5C

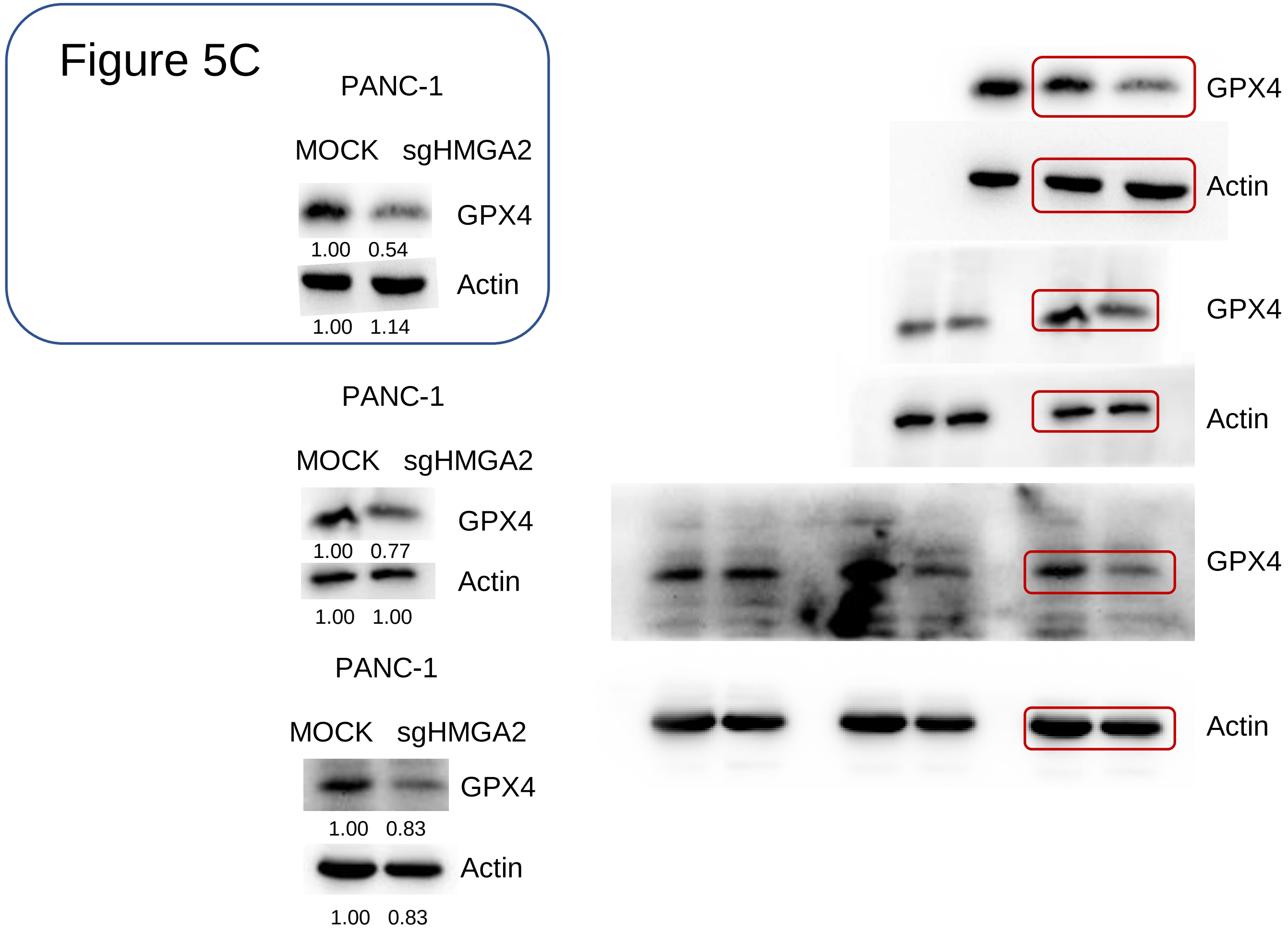
PANC-1
MOCK sgHMGA2
GPX4
1.00 0.54
Actin
1.00 1.14
PANC-1
MOCK sgHMGA2
GPX4
1.00 0.77
Actin
1.00 1.00
PANC-1
MOCK sgHMGA2
GPX4
1.00 0.83
Actin
1.00 0.83
GPX4
Actin
GPX4
Actin
GPX4
Actin

Figure 5D

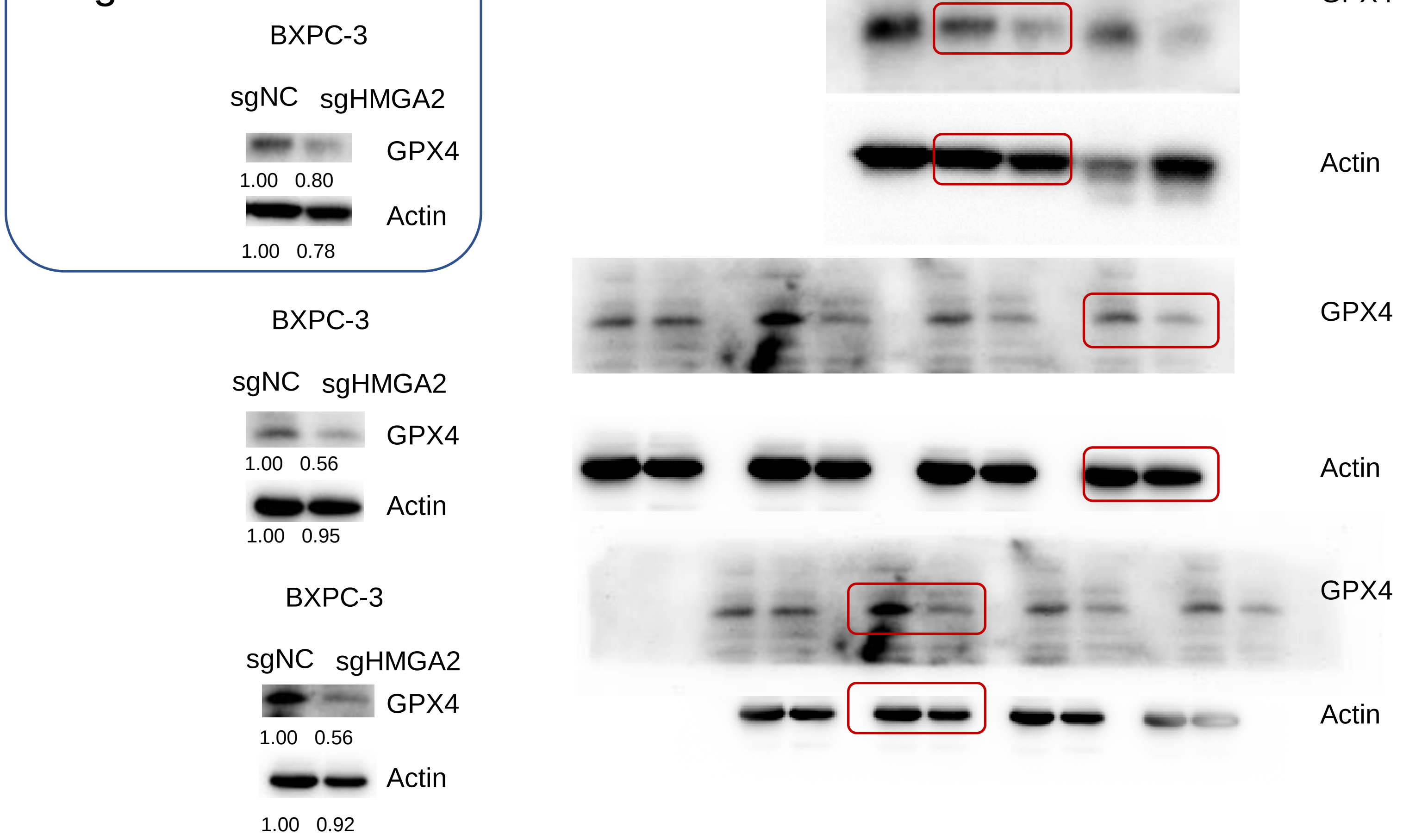
BXPC-3
sgNC
sgHMGA2
GPX4
1.00
0.80
Actin
1.00
0.78
BXPC-3
sgNC
sgHMGA2
GPX4
1.00
0.56
Actin
1.00
0.95
BXPC-3
sgNC
sgHMGA2
GPX4
1.00
0.56
Actin
1.00
0.92
GPX4
Actin
GPX4
Actin
GPX4
Actin

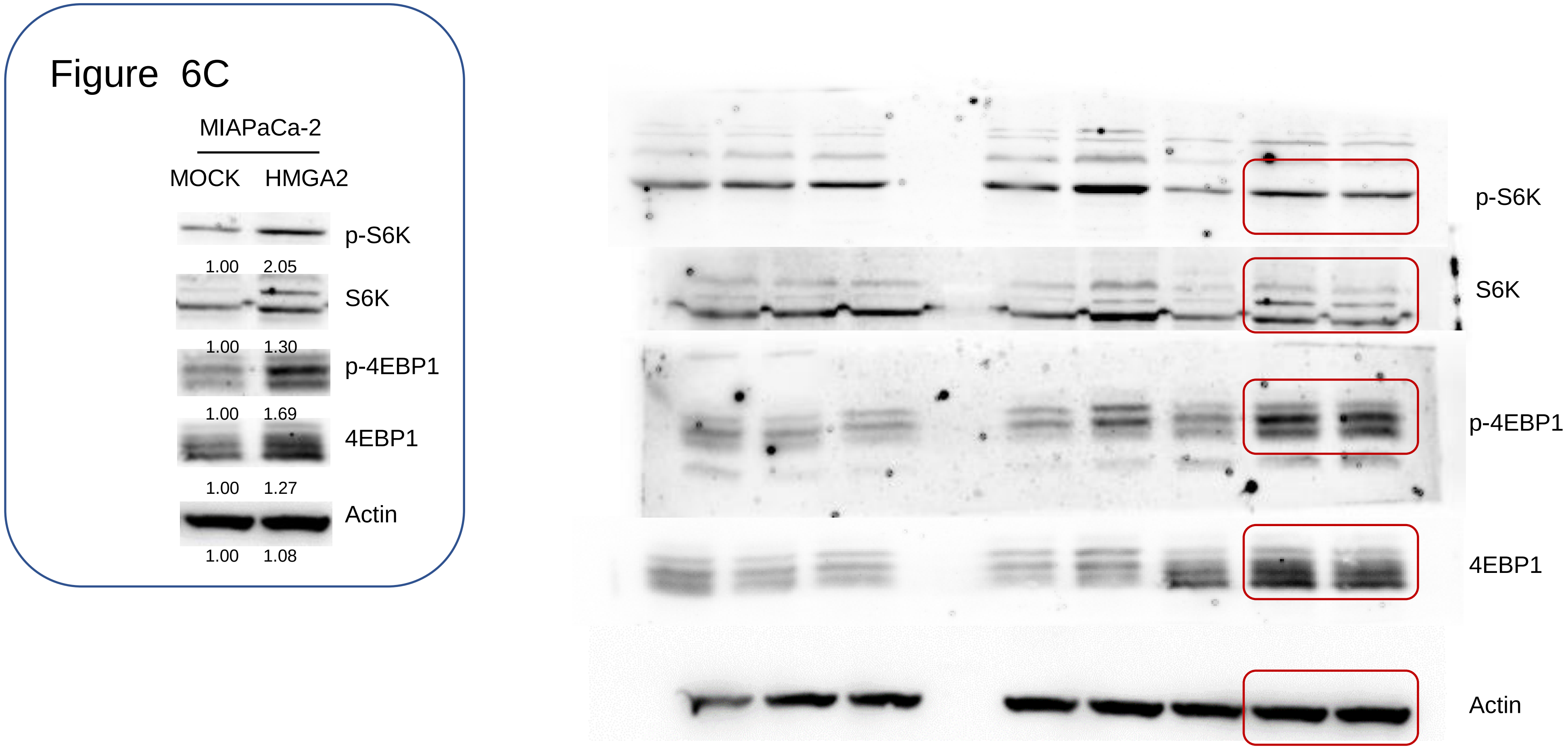
Figure 6C
MIAPaCa-2
MOCK HMGA2
p-S6K
1.00 2.05
S6K
1.00 1.30
p-4EBP1
1.00 1.69
4EBP1
1.00 1.27
Actin
1.00 1.08
p-S6K
S6K
p-4EBP1
4EBP1
Actin

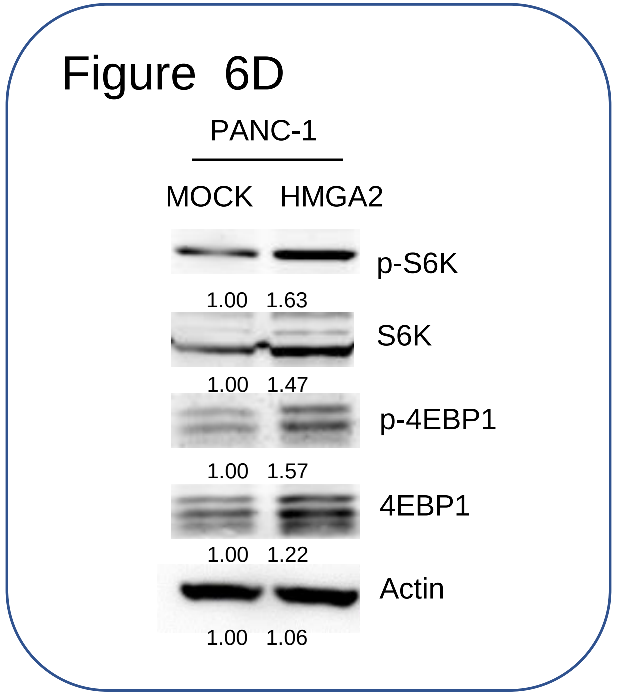
Figure 6D
PANC-1
MOCK
HMGA2
p-S6K
1.00 1.63
S6K
1.00 1.47
p-4EBP1
1.00 1.57
4EBP1
1.00 1.22
Actin
1.00 1.06

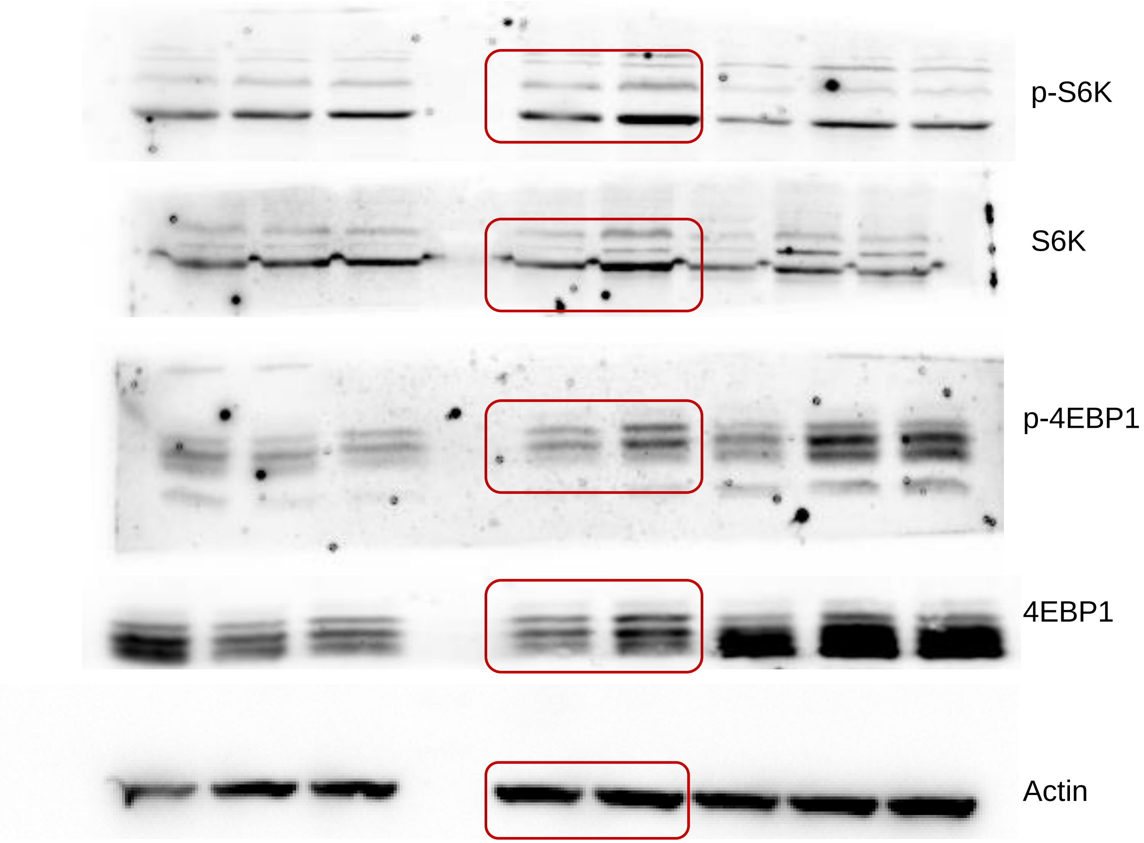
p-S6K
S6K
p-4EBP1
4EBP1
Actin

Figure 6E

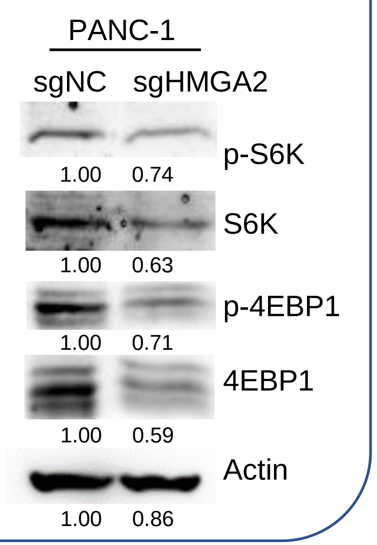
PANC-1
sgNC
sgHMGA2
p-S6K
1.00 0.74
S6K
1.00 0.63
p-4EBP1
1.00 0.71
4EBP1
1.00 0.59
Actin
1.00 0.86

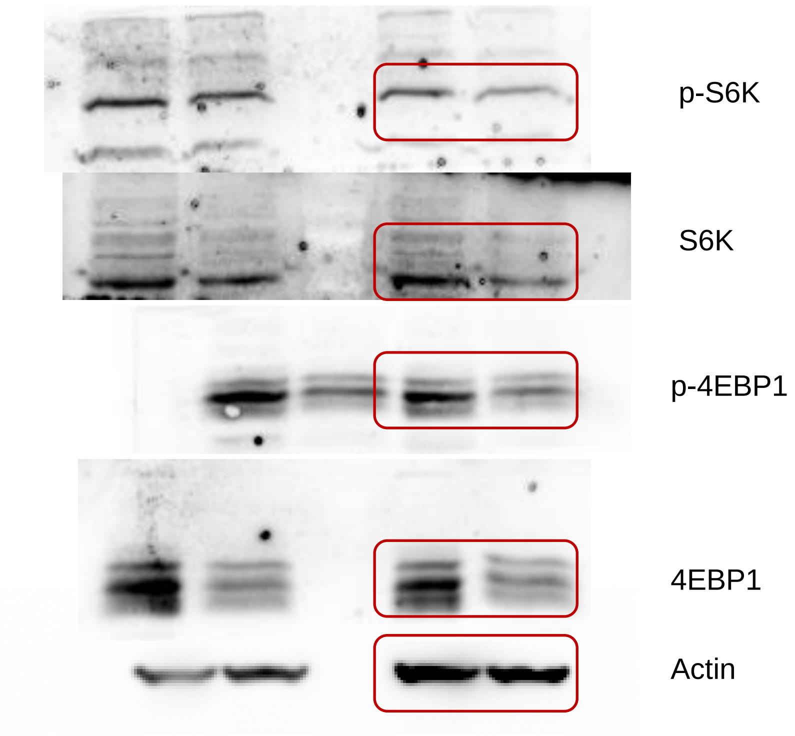
p-S6K
S6K
p-4EBP1
4EBP1
Actin

Figure 6F

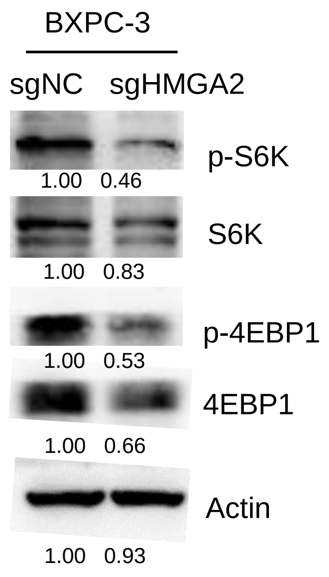
BXPC-3
sgNC
sgHMGA2
p-S6K
1.00 0.46
S6K
1.00 0.83
p-4EBP1
1.00 0.53
4EBP1
1.00 0.66
Actin
1.00 0.93

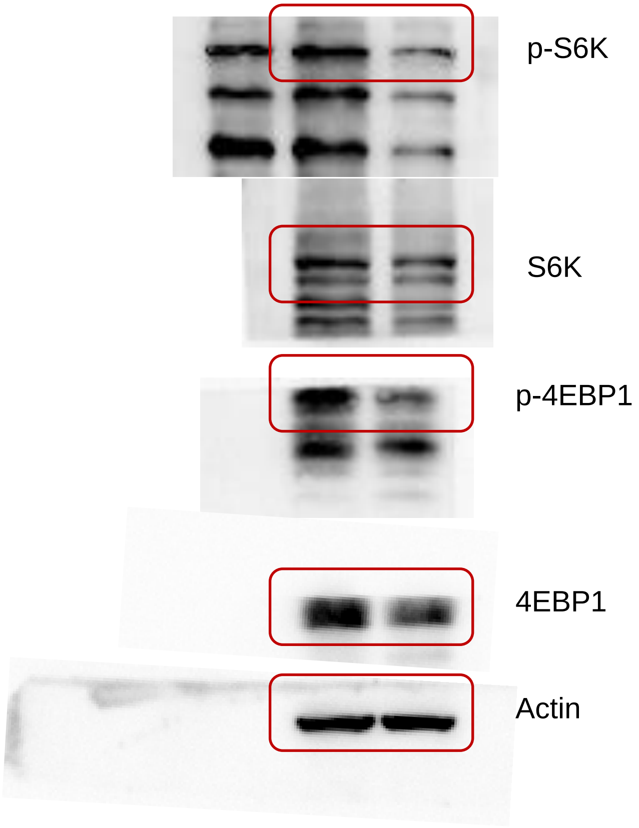
p-S6K
S6K
p-4EBP1
4EBP1
Actin

Figure 7A

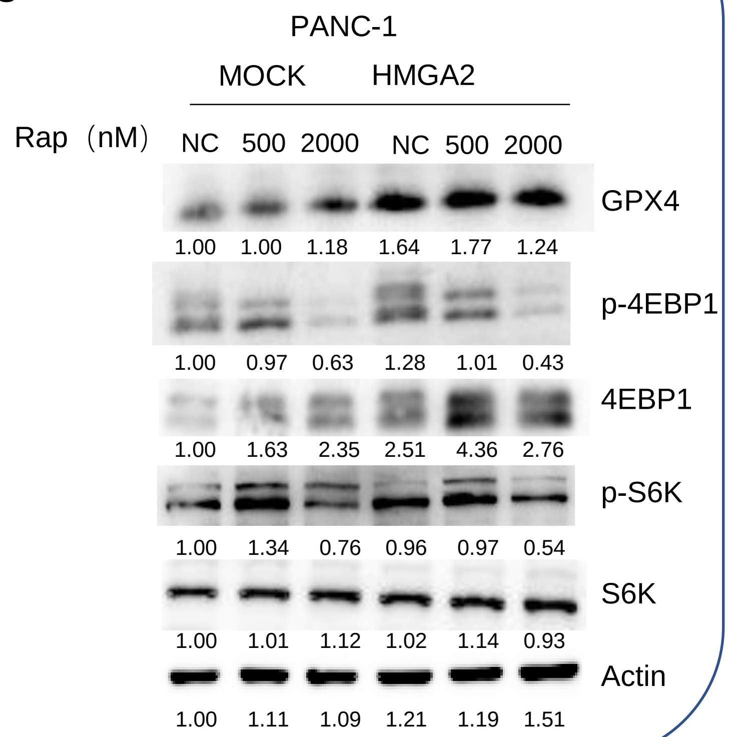
PANC-1
MOCK
HMGA2
Rap（nM）
NC 500 2000 NC 500 2000
GPX4
1.00 1.00 1.18 1.64 1.77 1.24
p-4EBP1
1.00 0.97 0.63 1.28 1.01 0.43
4EBP1
1.00 1.63 2.35 2.51 4.36 2.76
p-S6K
1.00 1.34 0.76 0.96 0.97 0.54
S6K
1.00 1.01 1.12 1.02 1.14 0.93
Actin
1.00 1.11 1.09 1.21 1.19 1.51

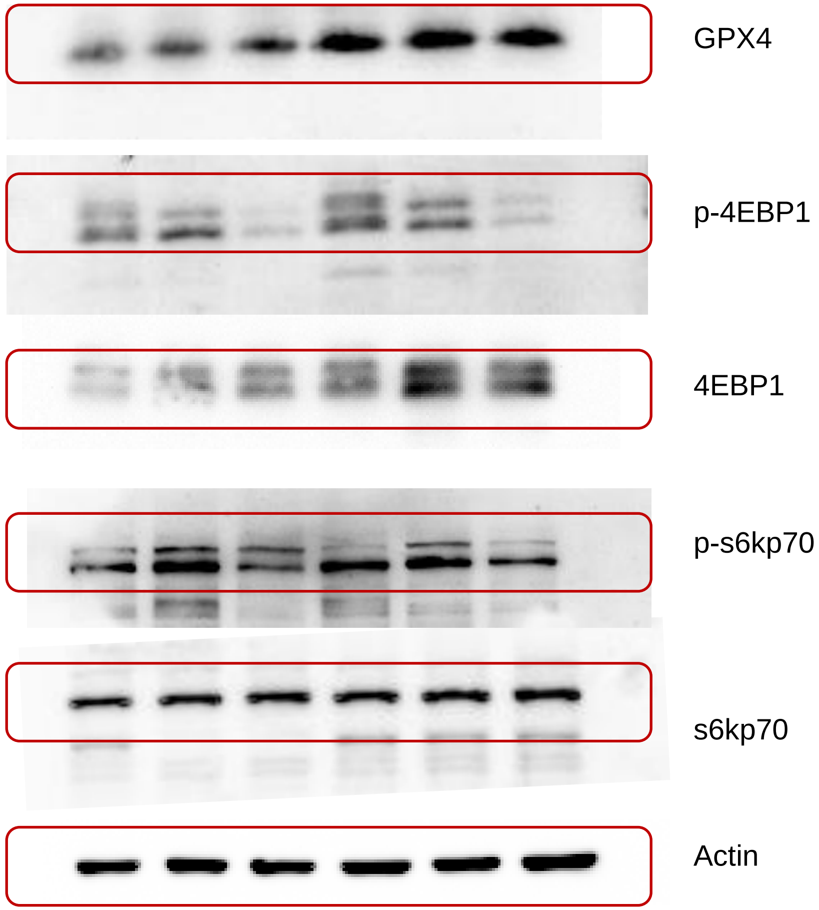
GPX4
p-4EBP1
4EBP1
p-s6kp70
s6kp70
Actin

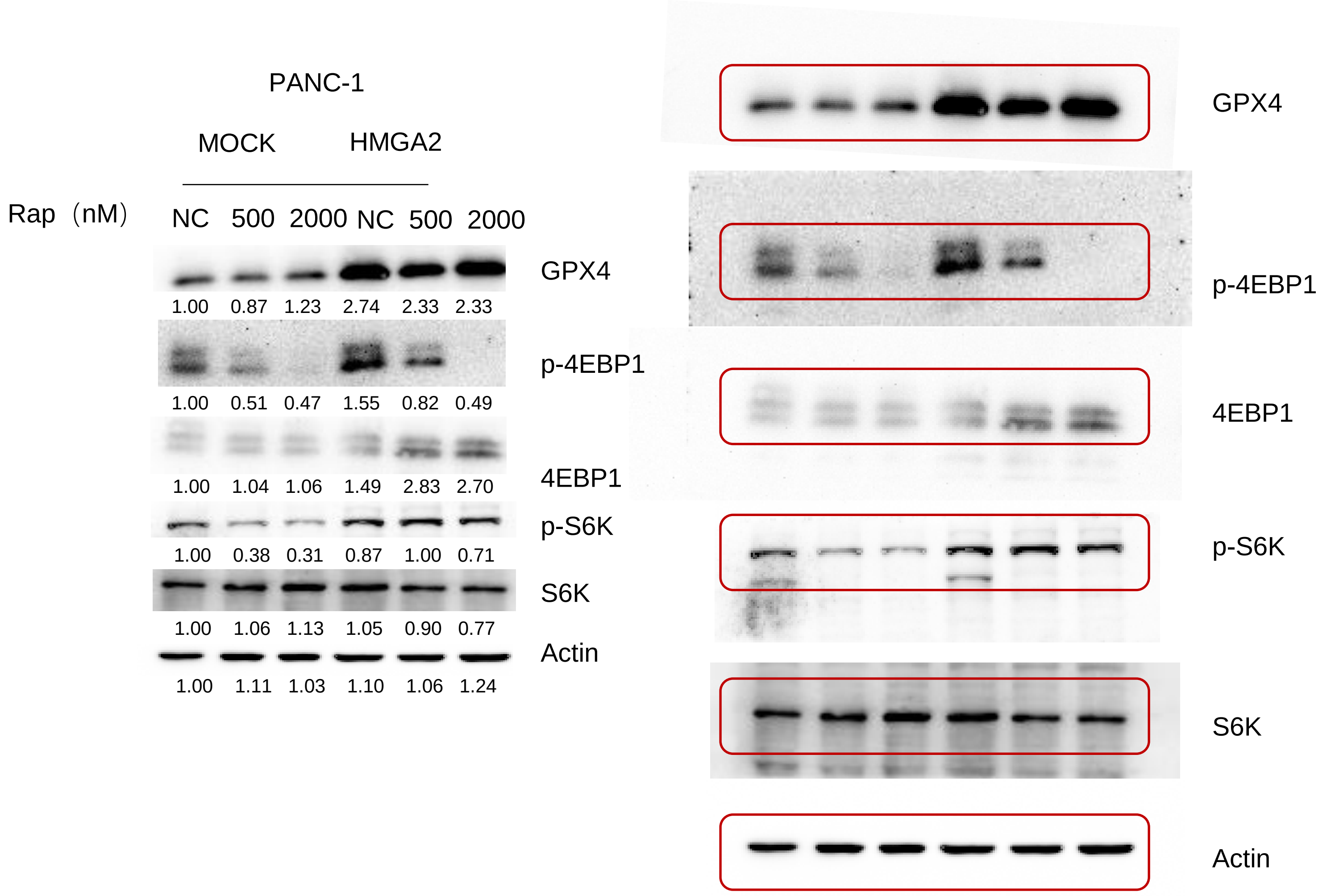
PANC-1
MOCK
HMGA2
Rap（nM）
NC 500 2000 NC 500 2000
GPX4
1.00 0.87 1.23 2.74 2.33 2.33
p-4EBP1
1.00 0.51 0.47 1.55 0.82 0.49
4EBP1
1.00 1.04 1.06 1.49 2.83 2.70
p-S6K
1.00 0.38 0.31 0.87 1.00 0.71
S6K
1.00 1.06 1.13 1.05 0.90 0.77
Actin
1.00 1.11 1.03 1.10 1.06 1.24
GPX4
p-4EBP1
4EBP1
p-S6K
S6K
Actin

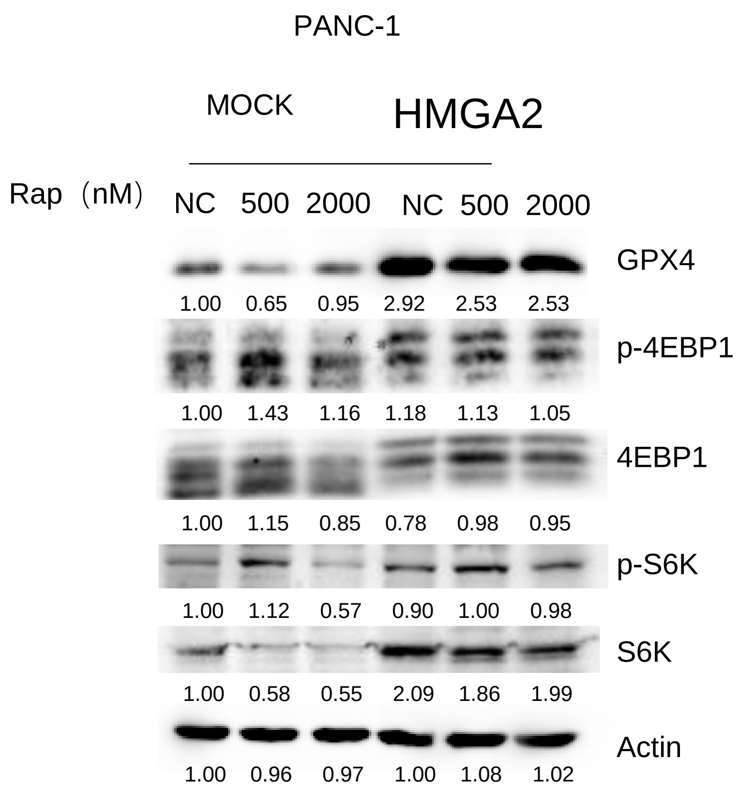
PANC-1
MOCK
HMGA2
Rap（nM）
NC 500 2000 NC 500 2000
GPX4
1.00 0.65 0.95 2.92 2.53 2.53
p-4EBP1
1.00 1.43 1.16 1.18 1.13 1.05
4EBP1
1.00 1.15 0.85 0.78 0.98 0.95
p-S6K
1.00 1.12 0.57 0.90 1.00 0.98
S6K
1.00 0.58 0.55 2.09 1.86 1.99
Actin
1.00 0.96 0.97 1.00 1.08 1.02

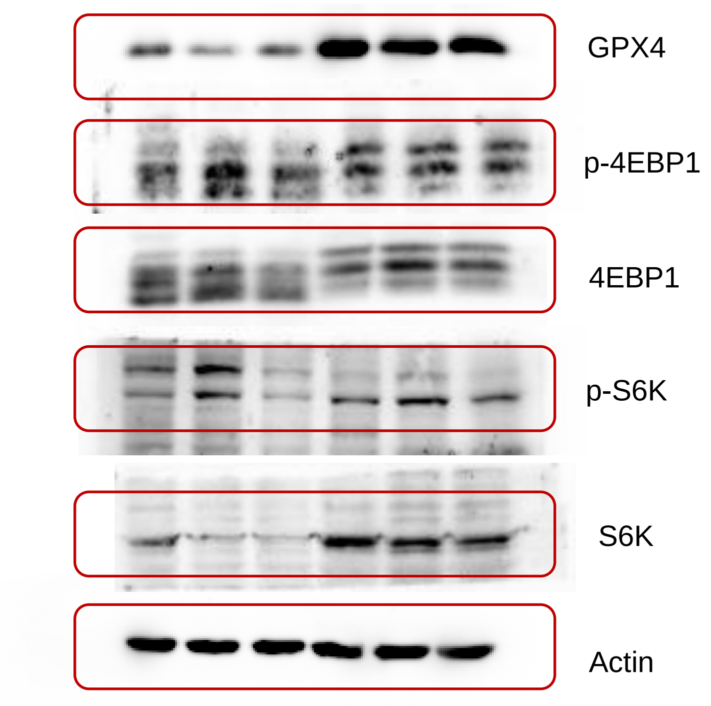
GPX4
p-4EBP1
4EBP1
p-S6K
S6K
Actin

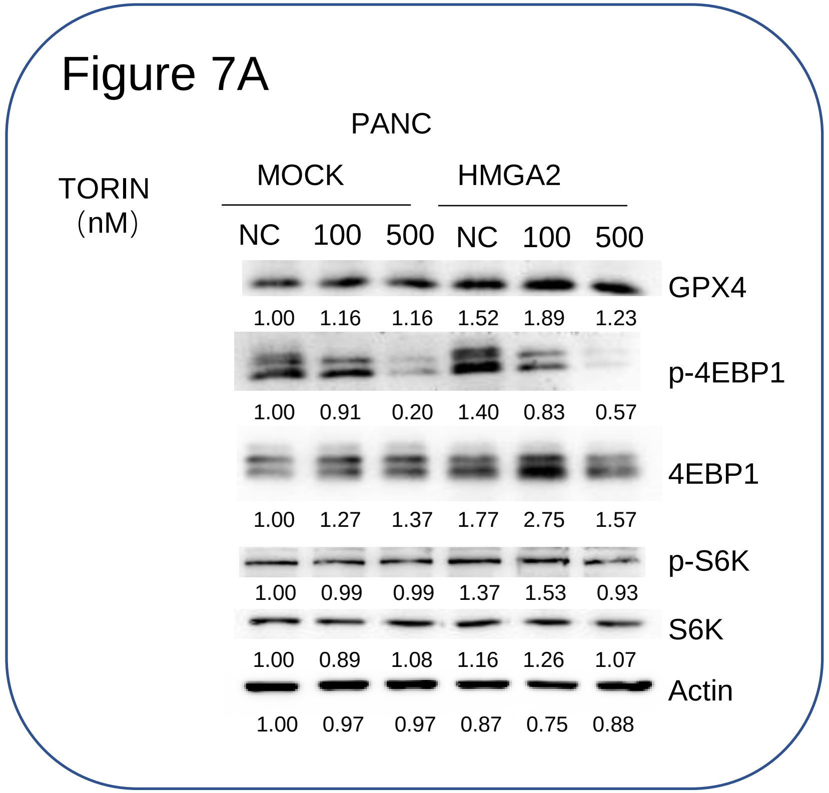
Figure 7A
PANC
TORIN (nM)
MOCK
HMGA2
NC 100 500 NC 100 500
GPX4
1.00 1.16 1.16 1.52 1.89 1.23
p-4EBP1
1.00 0.91 0.20 1.40 0.83 0.57
4EBP1
1.00 1.27 1.37 1.77 2.75 1.57
p-S6K
1.00 0.99 0.99 1.37 1.53 0.93
S6K
1.00 0.89 1.08 1.16 1.26 1.07
Actin
1.00 0.97 0.97 0.87 0.75 0.88

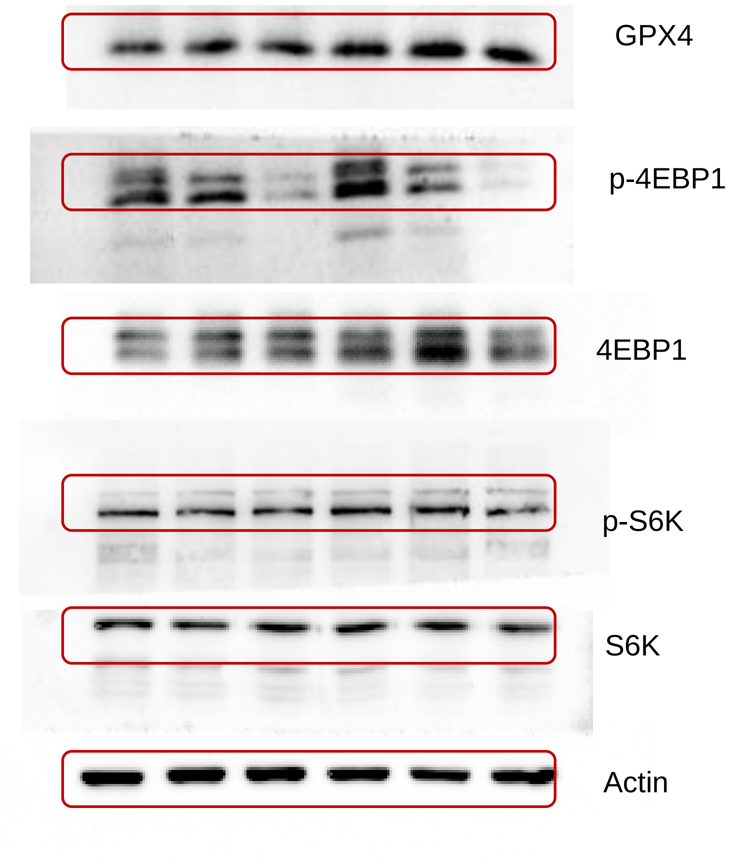
GPX4
p-4EBP1
4EBP1
p-S6K
S6K
Actin

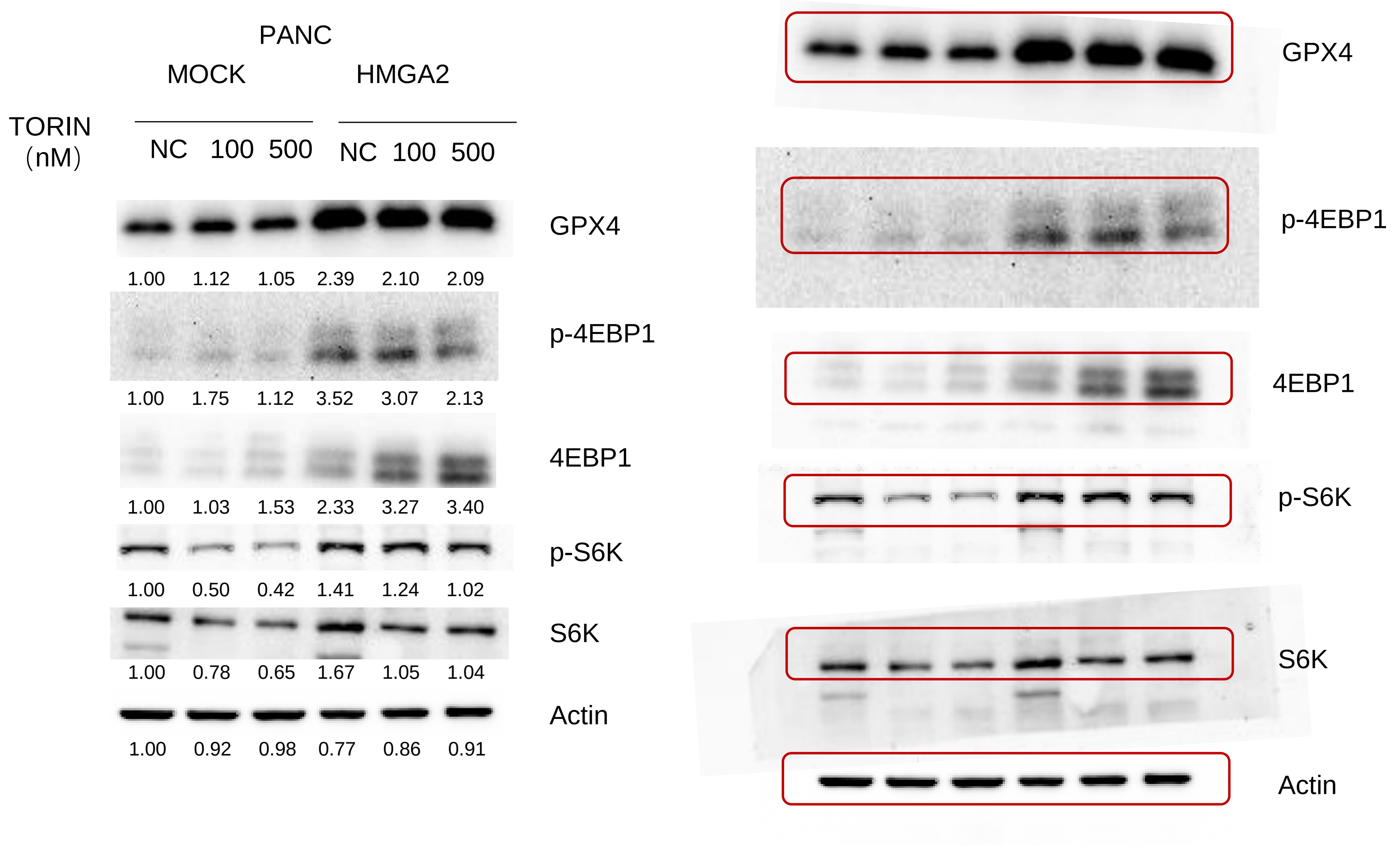
PANC
MOCK
HMGA2
TORIN (nM)
NC 100 500 NC 100 500
GPX4
1.00 1.12 1.05 2.39 2.10 2.09
p-4EBP1
1.00 1.75 1.12 3.52 3.07 2.13
4EBP1
1.00 1.03 1.53 2.33 3.27 3.40
p-S6K
1.00 0.50 0.42 1.41 1.24 1.02
S6K
1.00 0.78 0.65 1.67 1.05 1.04
Actin
1.00 0.92 0.98 0.77 0.86 0.91
GPX4
p-4EBP1
4EBP1
p-S6K
S6K
Actin

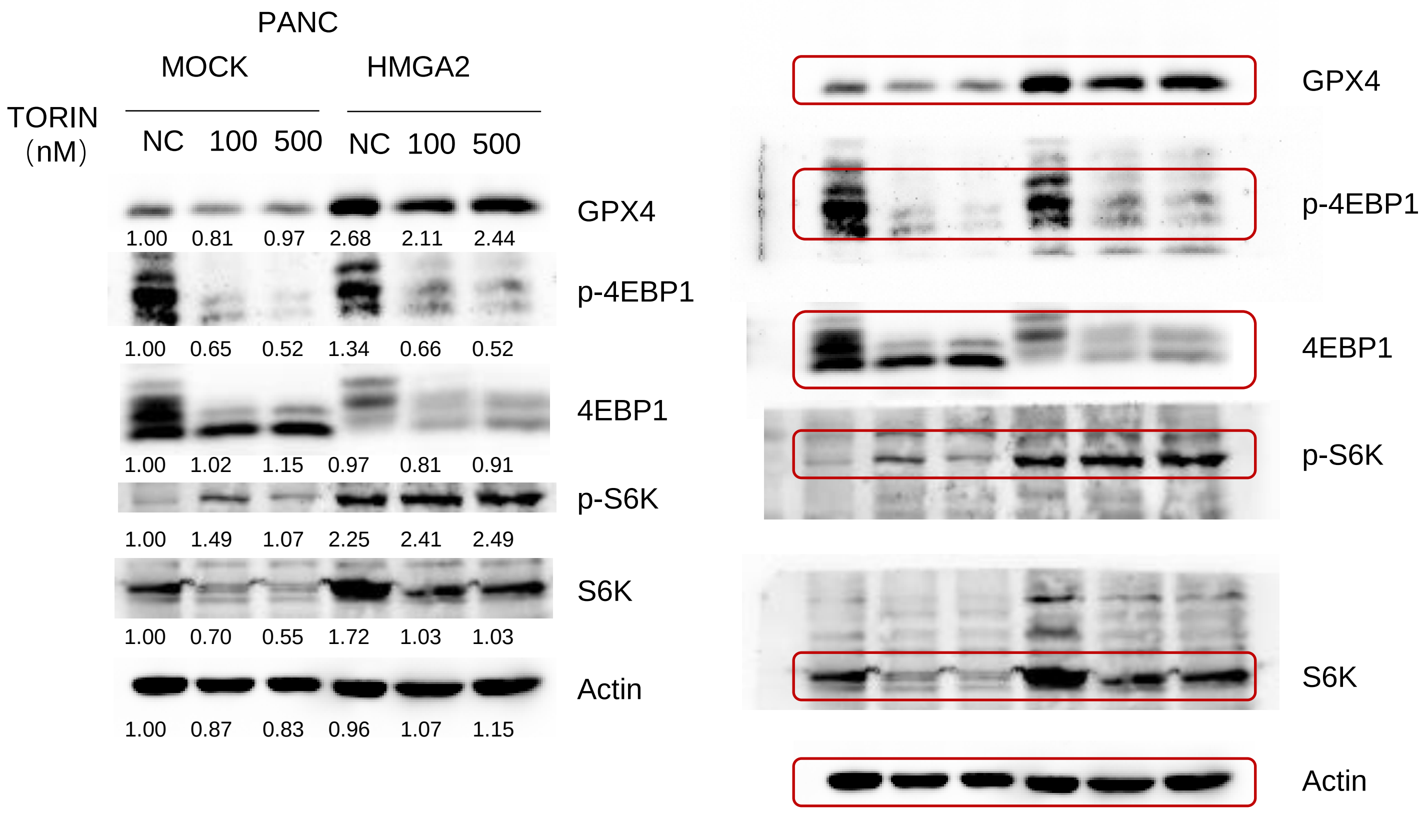

PANC
MOCK
HMGA2
TORIN (nM)
NC 100 500 NC 100 500
GPX4
1.00 0.81 0.97 2.68 2.11 2.44
p-4EBP1
1.00 0.65 0.52 1.34 0.66 0.52
4EBP1
1.00 1.02 1.15 0.97 0.81 0.91
p-S6K
1.00 1.49 1.07 2.25 2.41 2.49
S6K
1.00 0.70 0.55 1.72 1.03 1.03
Actin
1.00 0.87 0.83 0.96 1.07 1.15
GPX4
p-4EBP1
4EBP1
p-S6K
S6K
Actin

Figure 7B

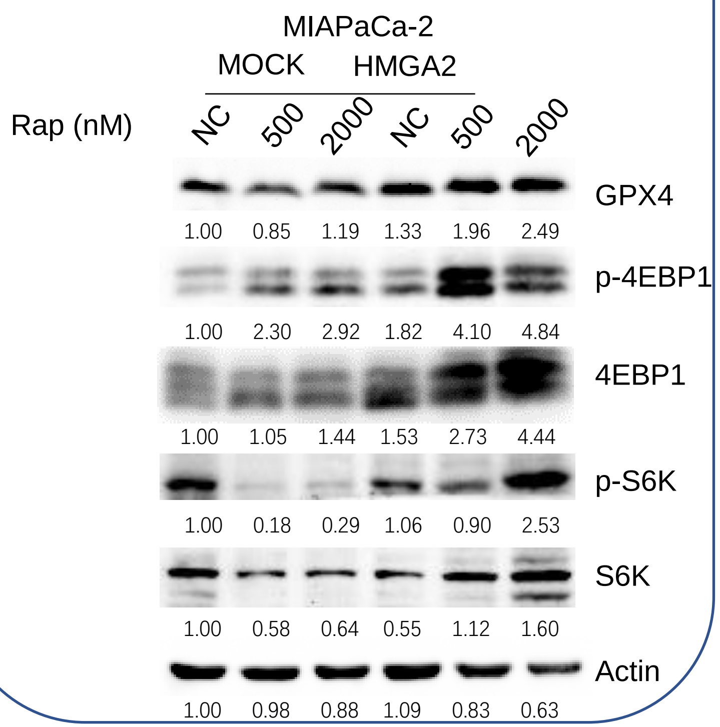

MIAPaCa-2
MOCK
HMGA2
Rap (nM)
NC
500
2000
NC
500
2000
GPX4
1.00 0.85 1.19 1.33 1.96 2.49
p-4EBP1
1.00 2.30 2.92 1.82 4.10 4.84
4EBP1
1.00 1.05 1.44 1.53 2.73 4.44
p-S6K
1.00 0.18 0.29 1.06 0.90 2.53
S6K
1.00 0.58 0.64 0.55 1.12 1.60
Actin
1.00 0.98 0.88 1.09 0.83 0.63

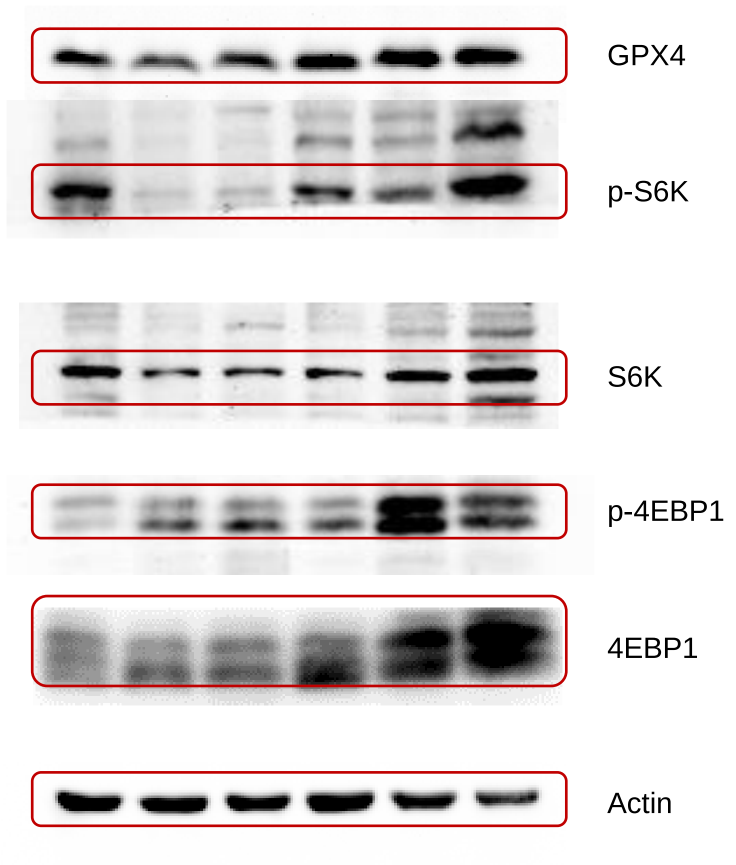

GPX4
p-S6K
S6K
p-4EBP1
4EBP1
Actin

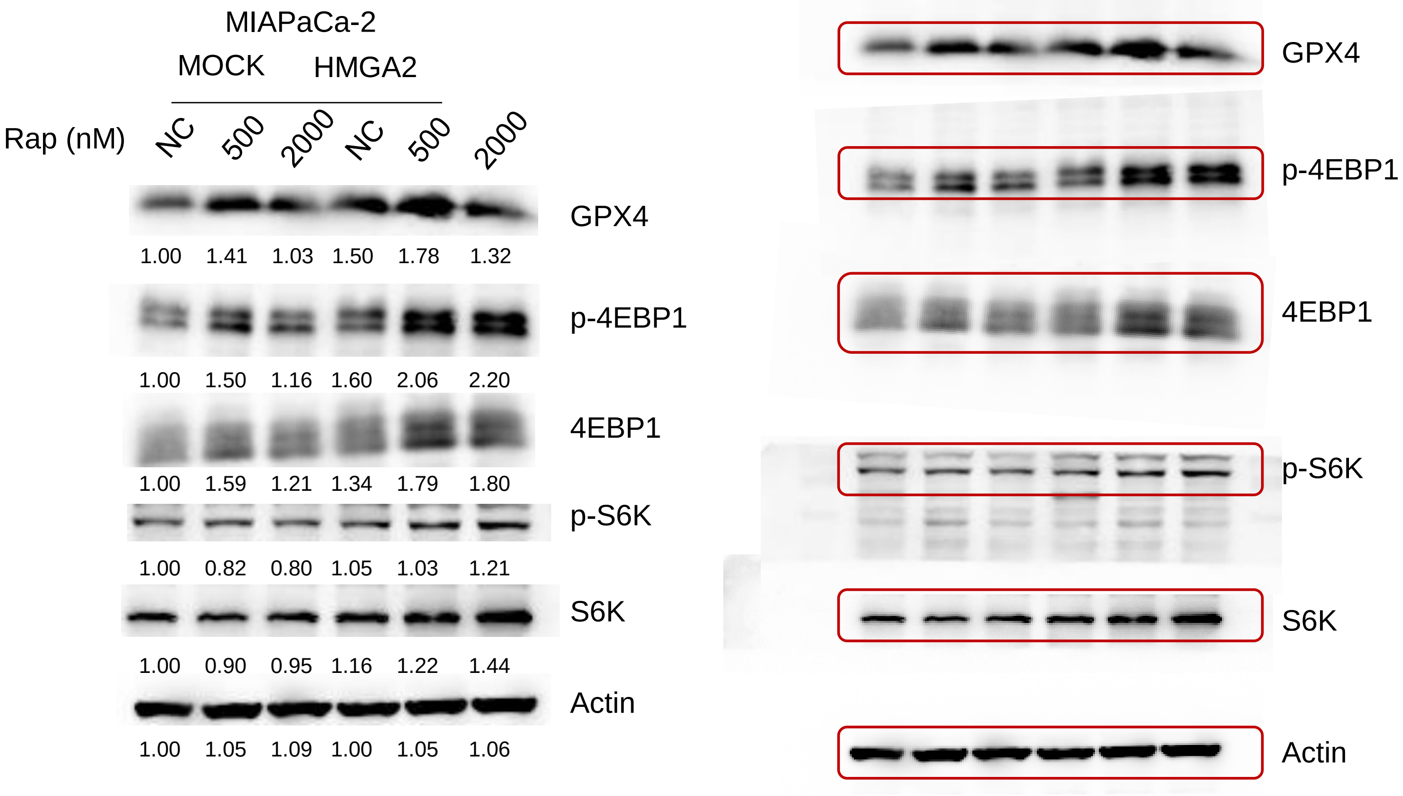
MIAPaCa-2
MOCK HMGA2
Rap (nM) NC 500 2000 NC 500 2000
GPX4
1.00 1.41 1.03 1.50 1.78 1.32
p-4EBP1
1.00 1.50 1.16 1.60 2.06 2.20
4EBP1
1.00 1.59 1.21 1.34 1.79 1.80
p-S6K
1.00 0.82 0.80 1.05 1.03 1.21
S6K
1.00 0.90 0.95 1.16 1.22 1.44
Actin
1.00 1.05 1.09 1.00 1.05 1.06
GPX4
p-4EBP1
4EBP1
p-S6K
S6K
Actin

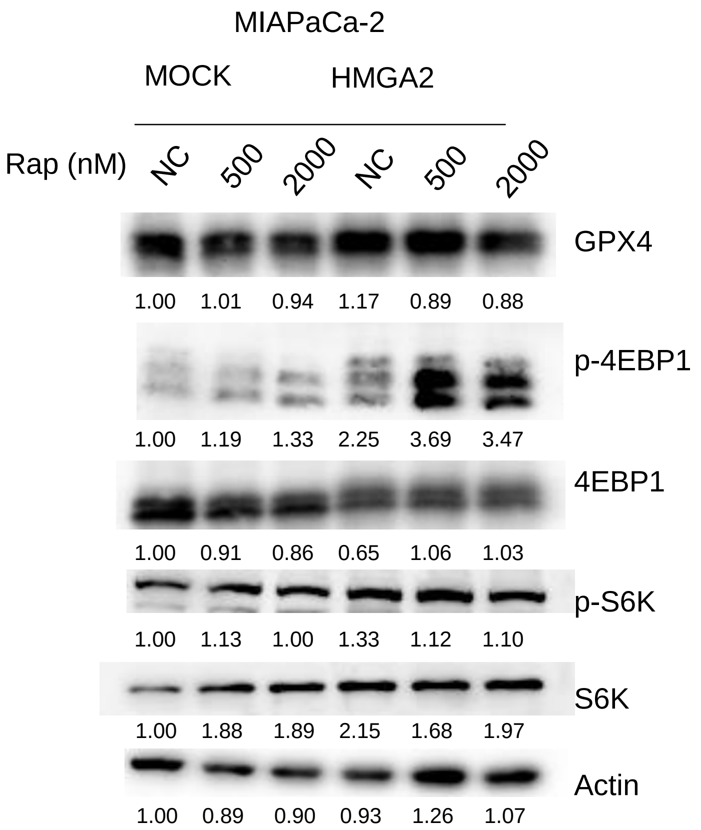
MIAPaCa-2
MOCK
HMGA2
Rap (nM)
NC
500
2000
NC
500
2000
GPX4
1.00 1.01 0.94 1.17 0.89 0.88
p-4EBP1
1.00 1.19 1.33 2.25 3.69 3.47
4EBP1
1.00 0.91 0.86 0.65 1.06 1.03
p-S6K
1.00 1.13 1.00 1.33 1.12 1.10
S6K
1.00 1.88 1.89 2.15 1.68 1.97
Actin
1.00 0.89 0.90 0.93 1.26 1.07

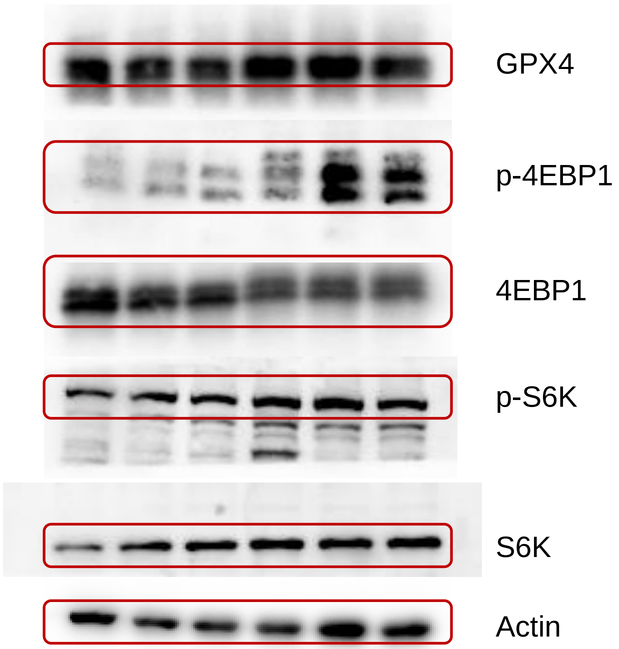
GPX4
p-4EBP1
4EBP1
p-S6K
S6K
Actin

Figure 7B

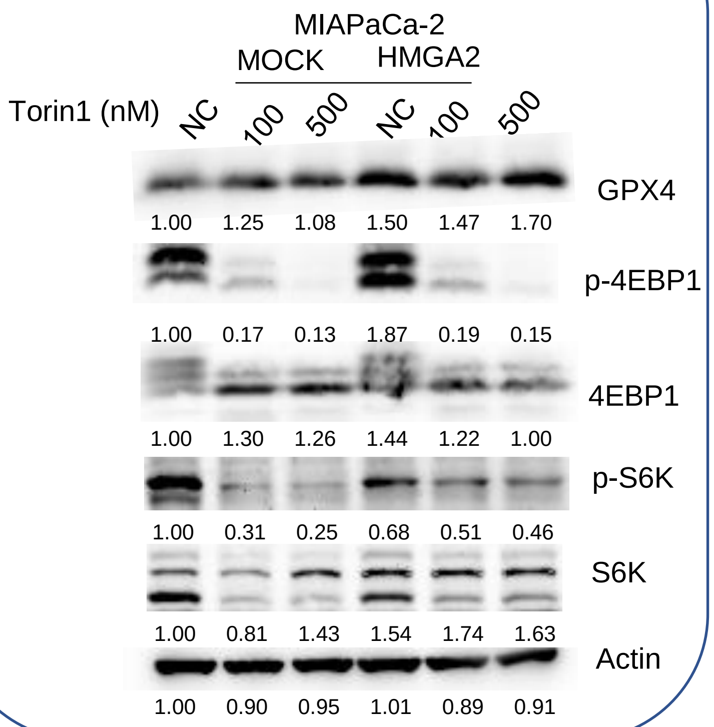
MIAPaCa-2
MOCK
HMGA2
Torin1 (nM)
NC
100
500
NC
100
500
GPX4
1.00 1.25 1.08 1.50 1.47 1.70
p-4EBP1
1.00 0.17 0.13 1.87 0.19 0.15
4EBP1
1.00 1.30 1.26 1.44 1.22 1.00
p-S6K
1.00 0.31 0.25 0.68 0.51 0.46
S6K
1.00 0.81 1.43 1.54 1.74 1.63
Actin
1.00 0.90 0.95 1.01 0.89 0.91

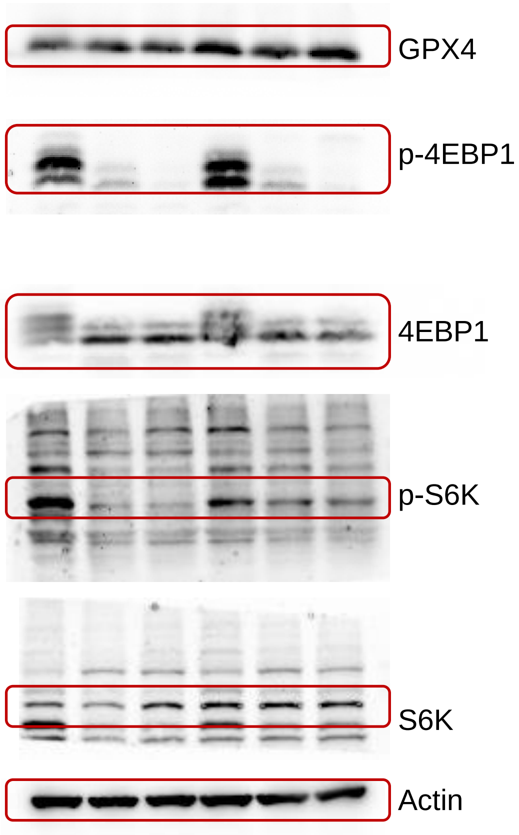
GPX4
p-4EBP1
4EBP1
p-S6K
S6K
Actin

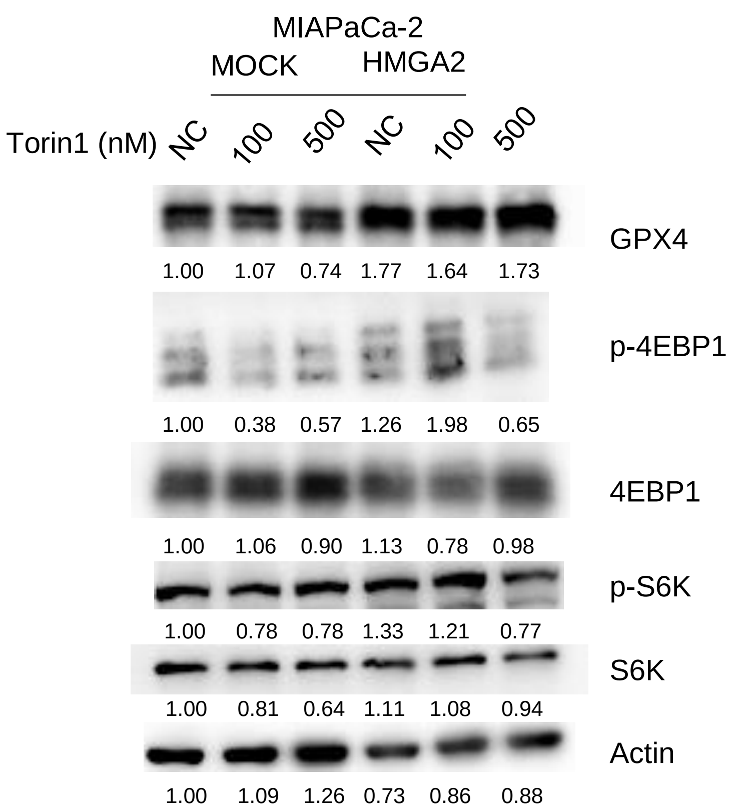
MIAPaCa-2
MOCK
HMGA2
Torin1 (nM)
NC
100
500
NC
100
500
GPX4
1.00 1.07 0.74 1.77 1.64 1.73
p-4EBP1
1.00 0.38 0.57 1.26 1.98 0.65
4EBP1
1.00 1.06 0.90 1.13 0.78 0.98
p-S6K
1.00 0.78 0.78 1.33 1.21 0.77
S6K
1.00 0.81 0.64 1.11 1.08 0.94
Actin
1.00 1.09 1.26 0.73 0.86 0.88

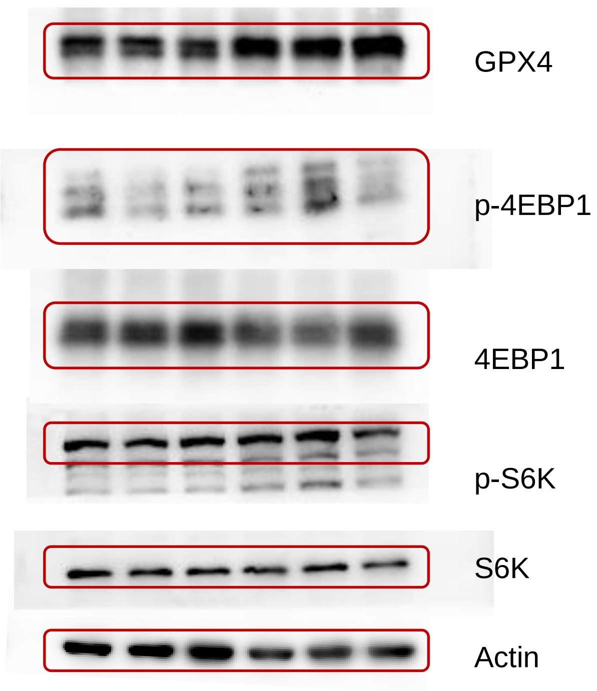
GPX4
p-4EBP1
4EBP1
p-S6K
S6K
Actin

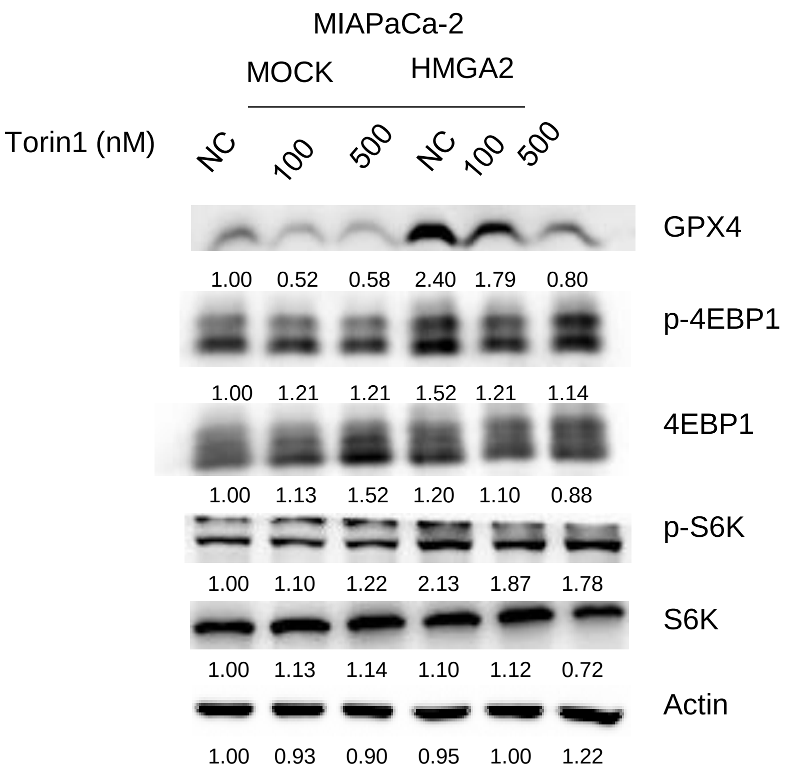
MIAPaCa-2
MOCK
HMGA2
Torin1 (nM)
NC
100
500
NC
100
500
GPX4
1.00 0.52 0.58 2.40 1.79 0.80
p-4EBP1
1.00 1.21 1.21 1.52 1.21 1.14
4EBP1
1.00 1.13 1.52 1.20 1.10 0.88
p-S6K
1.00 1.10 1.22 2.13 1.87 1.78
S6K
1.00 1.13 1.14 1.10 1.12 0.72
Actin
1.00 0.93 0.90 0.95 1.00 1.22

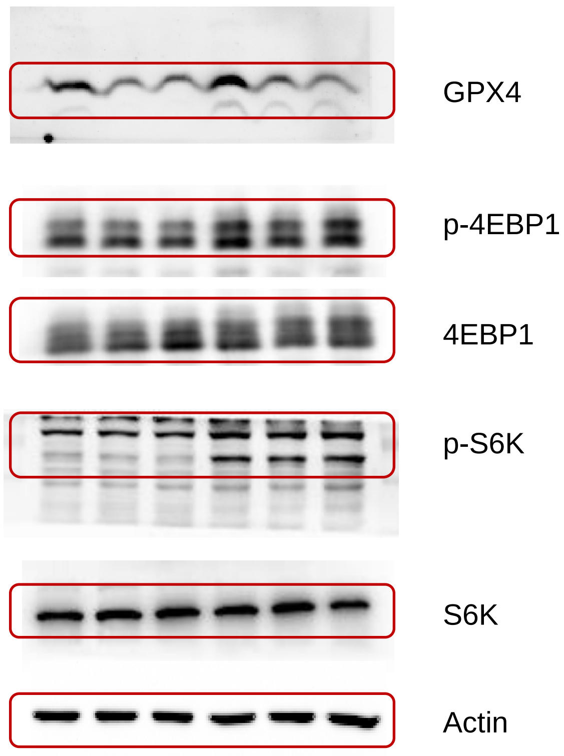
GPX4
p-4EBP1
4EBP1
p-S6K
S6K
Actin

Figure 7C

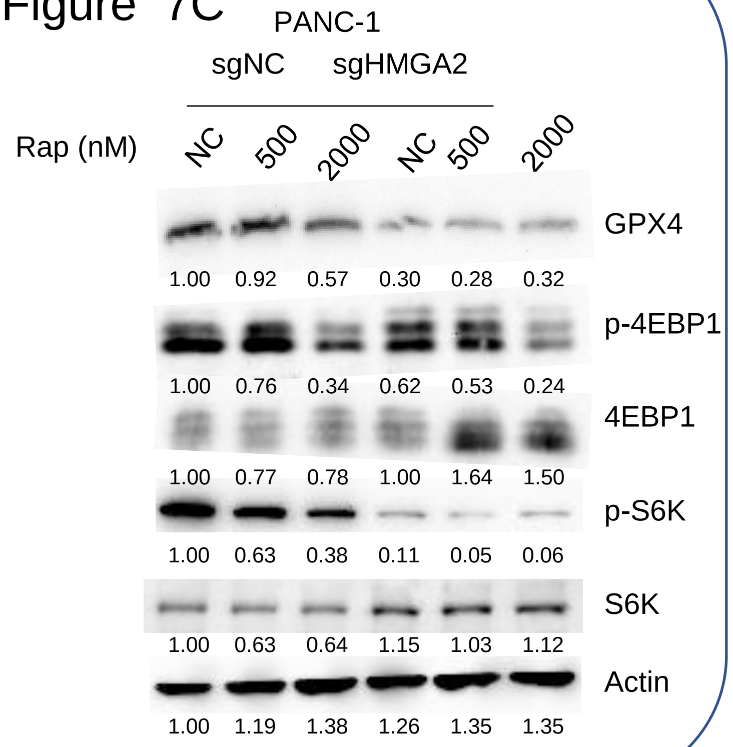

PANC-1
sgNC
sgHMGA2
Rap (nM)
NC
500
2000
NC
500
2000
GPX4
1.00 0.92 0.57 0.30 0.28 0.32
p-4EBP1
1.00 0.76 0.34 0.62 0.53 0.24
4EBP1
1.00 0.77 0.78 1.00 1.64 1.50
p-S6K
1.00 0.63 0.38 0.11 0.05 0.06
S6K
1.00 0.63 0.64 1.15 1.03 1.12
Actin
1.00 1.19 1.38 1.26 1.35 1.35

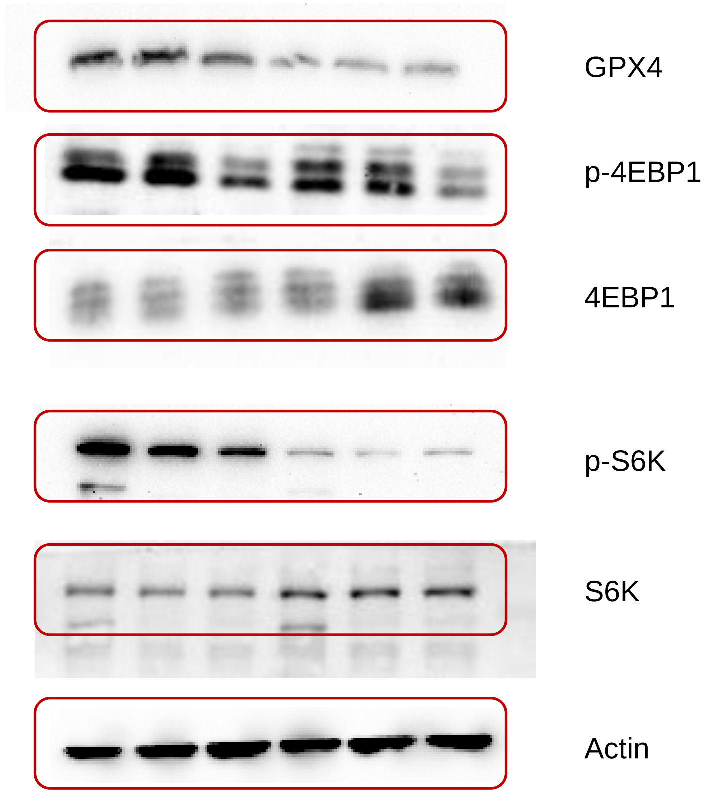

GPX4
p-4EBP1
4EBP1
p-S6K
S6K
Actin

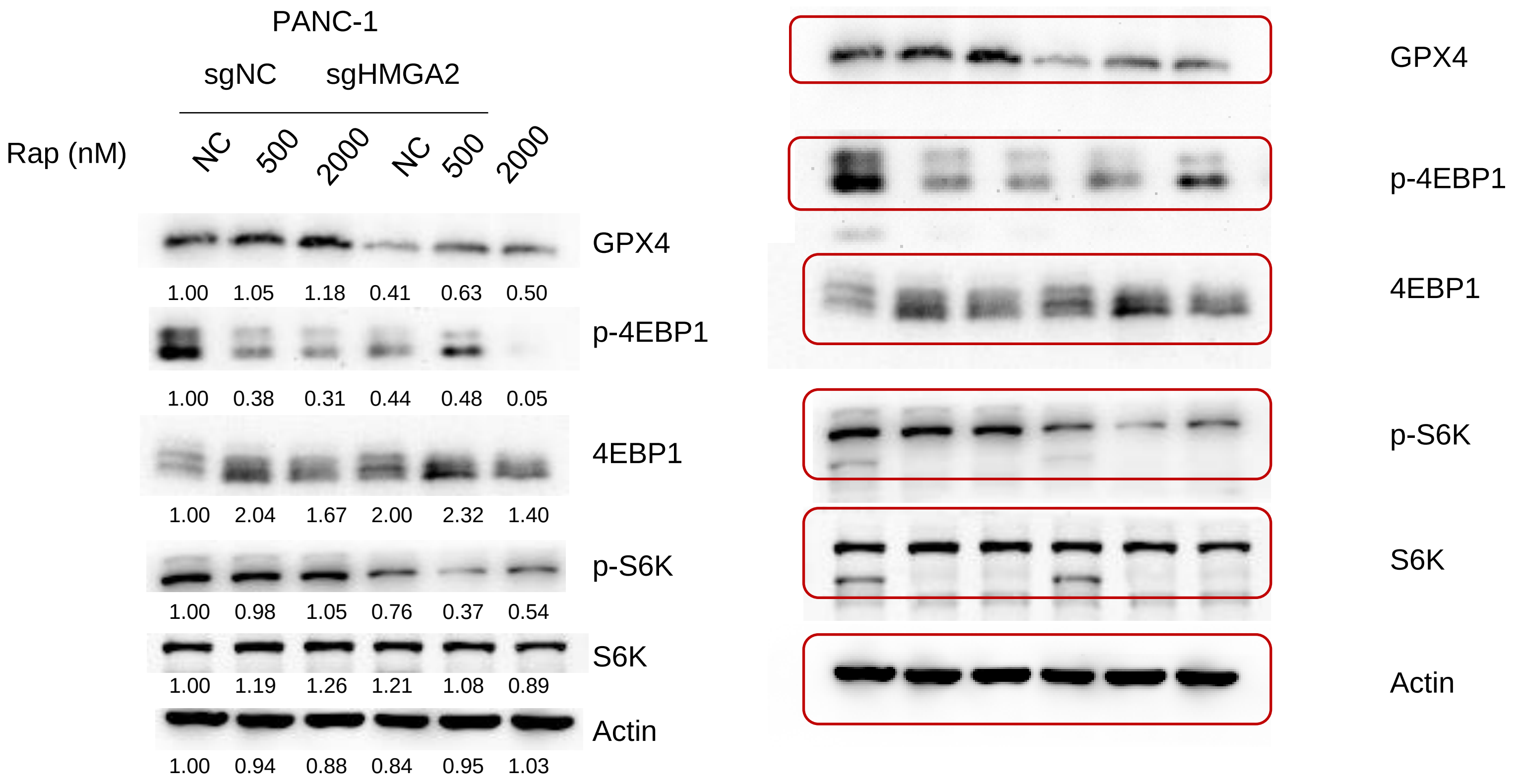
PANC-1
sgNC
sgHMGA2
Rap (nM)
NC
500
2000
NC
500
2000
GPX4
1.00 1.05 1.18 0.41 0.63 0.50
p-4EBP1
1.00 0.38 0.31 0.44 0.48 0.05
4EBP1
1.00 2.04 1.67 2.00 2.32 1.40
p-S6K
1.00 0.98 1.05 0.76 0.37 0.54
S6K
1.00 1.19 1.26 1.21 1.08 0.89
Actin
1.00 0.94 0.88 0.84 0.95 1.03
GPX4
p-4EBP1
4EBP1
p-S6K
S6K
Actin

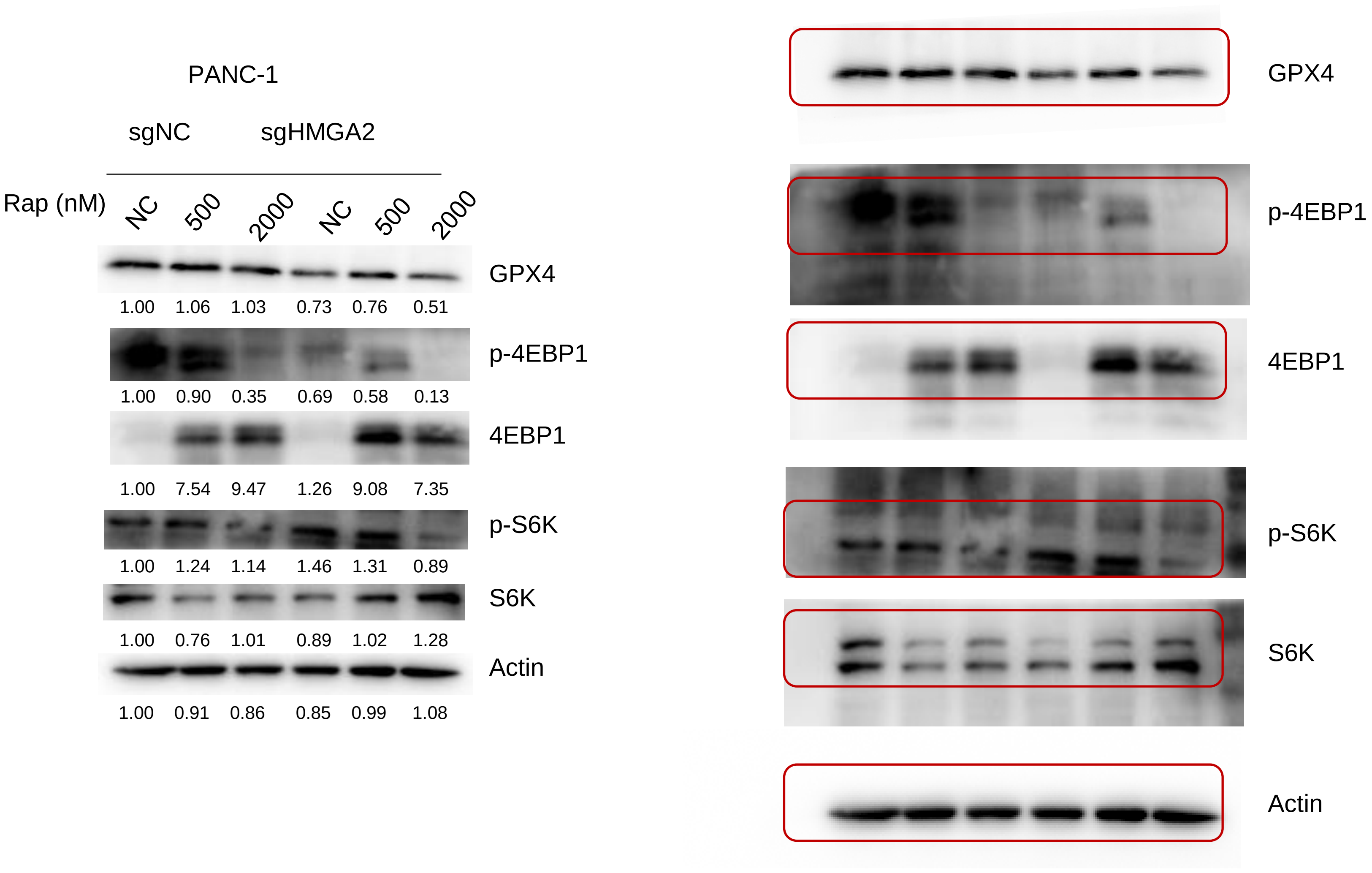
PANC-1
sgNC
sgHMGA2
Rap (nM)
NC
500
2000
NC
500
2000
GPX4
1.00 1.06 1.03 0.73 0.76 0.51
p-4EBP1
1.00 0.90 0.35 0.69 0.58 0.13
4EBP1
1.00 7.54 9.47 1.26 9.08 7.35
p-S6K
1.00 1.24 1.14 1.46 1.31 0.89
S6K
1.00 0.76 1.01 0.89 1.02 1.28
Actin
1.00 0.91 0.86 0.85 0.99 1.08
GPX4
p-4EBP1
4EBP1
p-S6K
S6K
Actin

Figure 7C

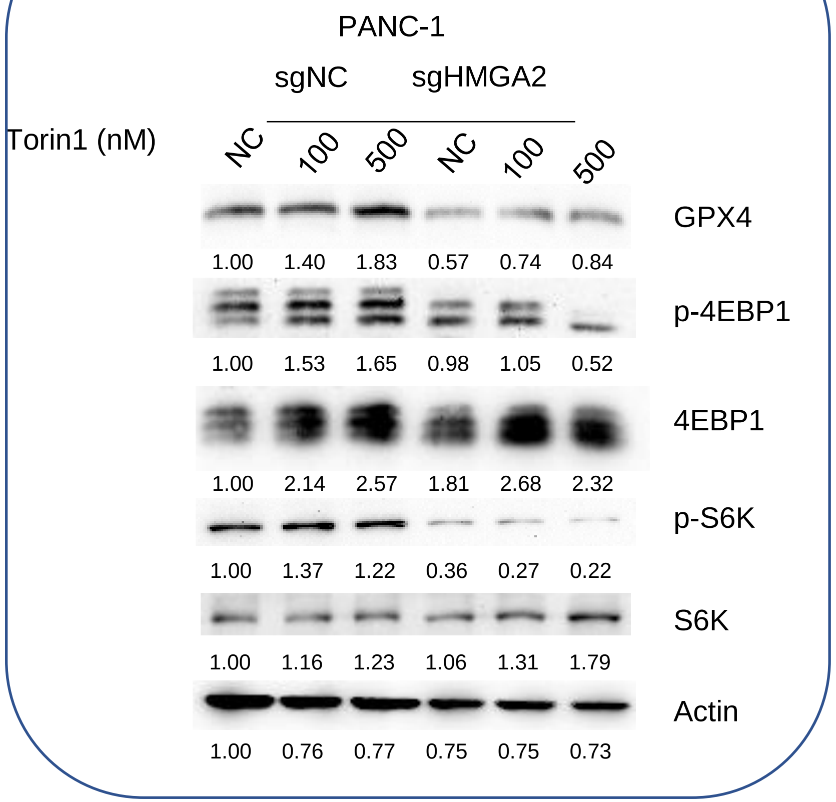

PANC-1
sgNC
sgHMGA2
Torin1 (nM)
NC
100
500
NC
100
500
GPX4
1.00 1.40 1.83 0.57 0.74 0.84
p-4EBP1
1.00 1.53 1.65 0.98 1.05 0.52
4EBP1
1.00 2.14 2.57 1.81 2.68 2.32
p-S6K
1.00 1.37 1.22 0.36 0.27 0.22
S6K
1.00 1.16 1.23 1.06 1.31 1.79
Actin
1.00 0.76 0.77 0.75 0.75 0.73

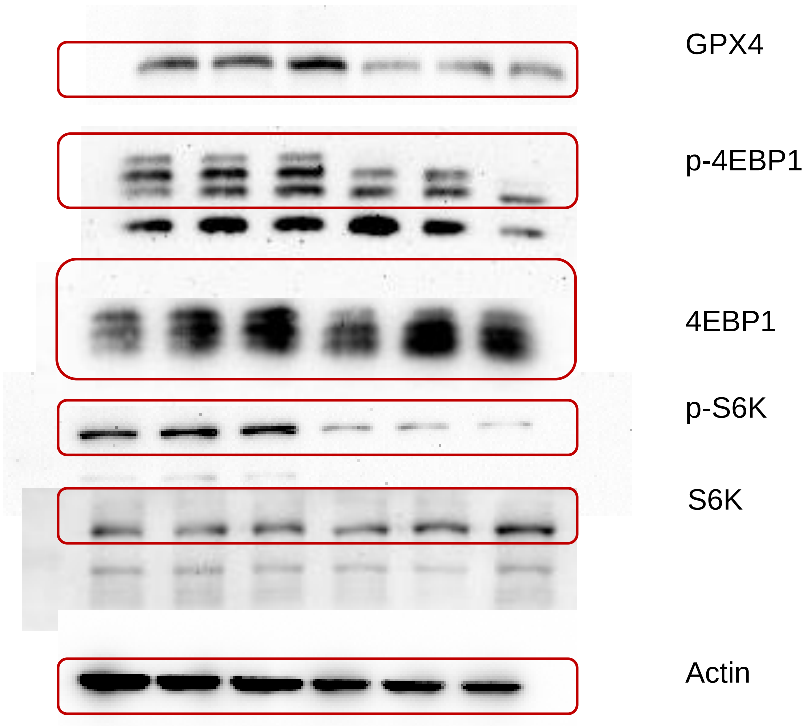

GPX4
p-4EBP1
4EBP1
p-S6K
S6K
Actin

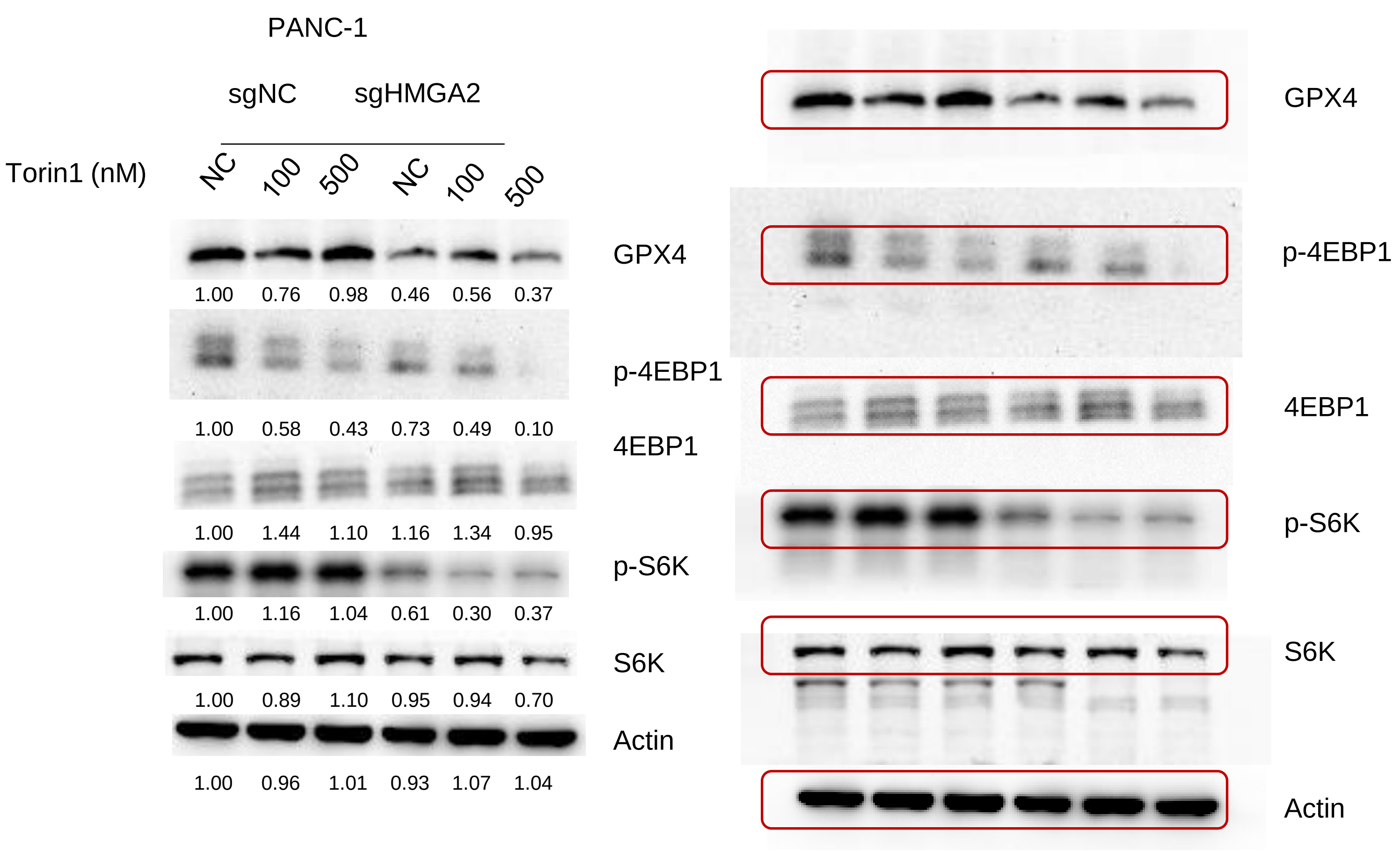
PANC-1
sgNC
sgHMGA2
Torin1 (nM)
NC
100
500
NC
100
500
GPX4
1.00 0.76 0.98 0.46 0.56 0.37
p-4EBP1
1.00 0.58 0.43 0.73 0.49 0.10
4EBP1
1.00 1.44 1.10 1.16 1.34 0.95
p-S6K
1.00 1.16 1.04 0.61 0.30 0.37
S6K
1.00 0.89 1.10 0.95 0.94 0.70
Actin
1.00 0.96 1.01 0.93 1.07 1.04
GPX4
p-4EBP1
4EBP1
p-S6K
S6K
Actin

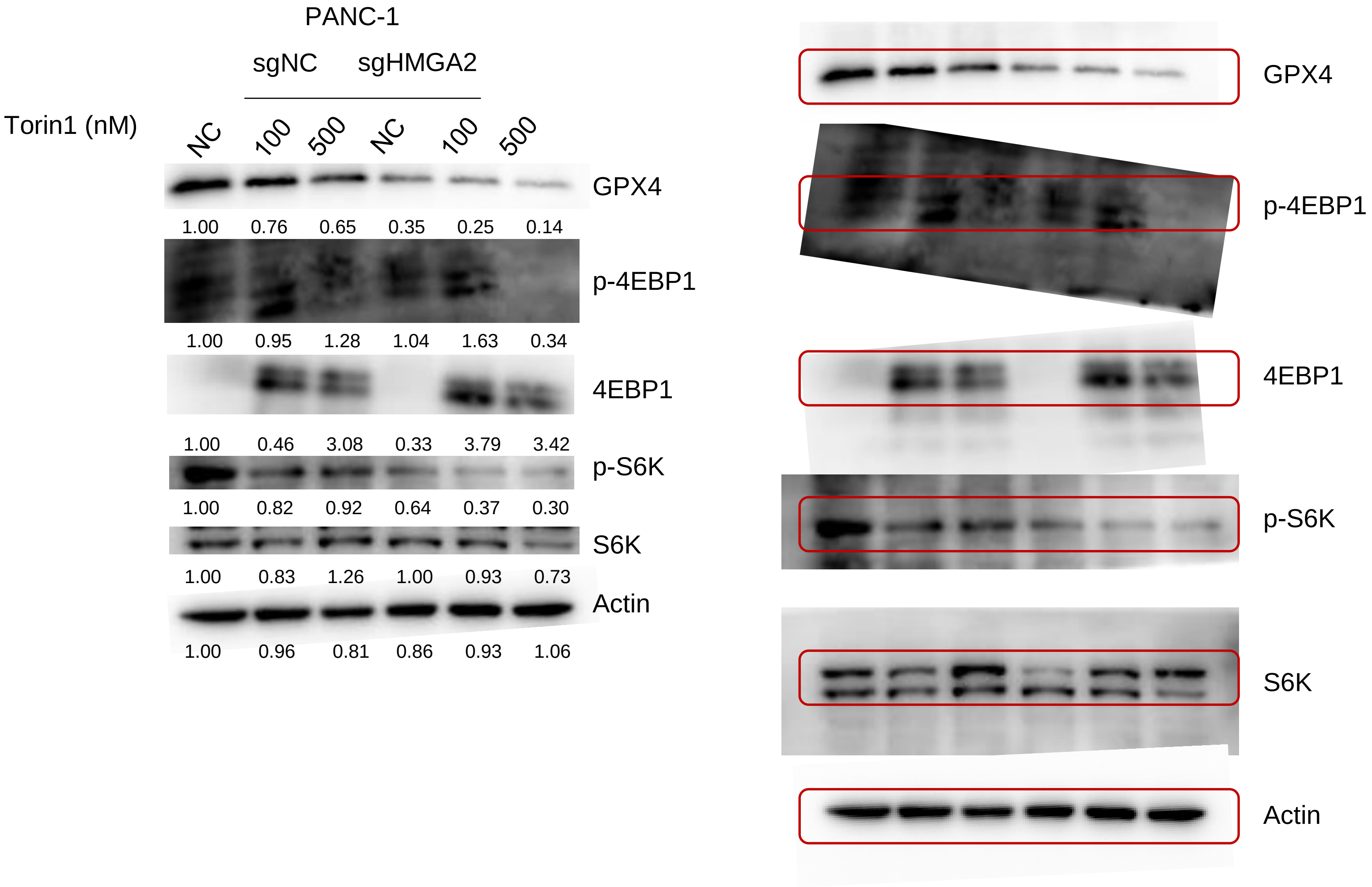
PANC-1
sgNC
sgHMGA2
Torin1 (nM)
NC
100
500
NC
100
500
GPX4
1.00 0.76 0.65 0.35 0.25 0.14
p-4EBP1
1.00 0.95 1.28 1.04 1.63 0.34
4EBP1
1.00 0.46 3.08 0.33 3.79 3.42
p-S6K
1.00 0.82 0.92 0.64 0.37 0.30
S6K
1.00 0.83 1.26 1.00 0.93 0.73
Actin
1.00 0.96 0.81 0.86 0.93 1.06
GPX4
p-4EBP1
4EBP1
p-S6K
S6K
Actin

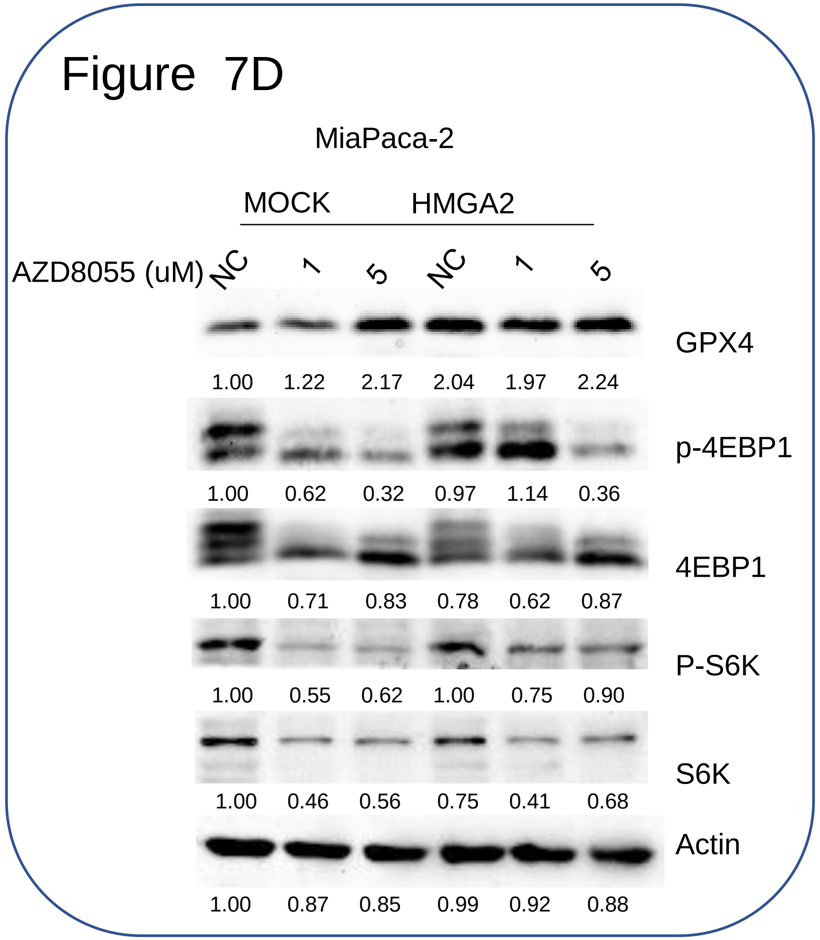
Figure 7D
MiaPaca-2
MOCK
HMGA2
AZD8055 (uM)
NC
1
5
NC
1
5
GPX4
1.00 1.22 2.17 2.04 1.97 2.24
p-4EBP1
1.00 0.62 0.32 0.97 1.14 0.36
4EBP1
1.00 0.71 0.83 0.78 0.62 0.87
P-S6K
1.00 0.55 0.62 1.00 0.75 0.90
S6K
1.00 0.46 0.56 0.75 0.41 0.68
Actin
1.00 0.87 0.85 0.99 0.92 0.88

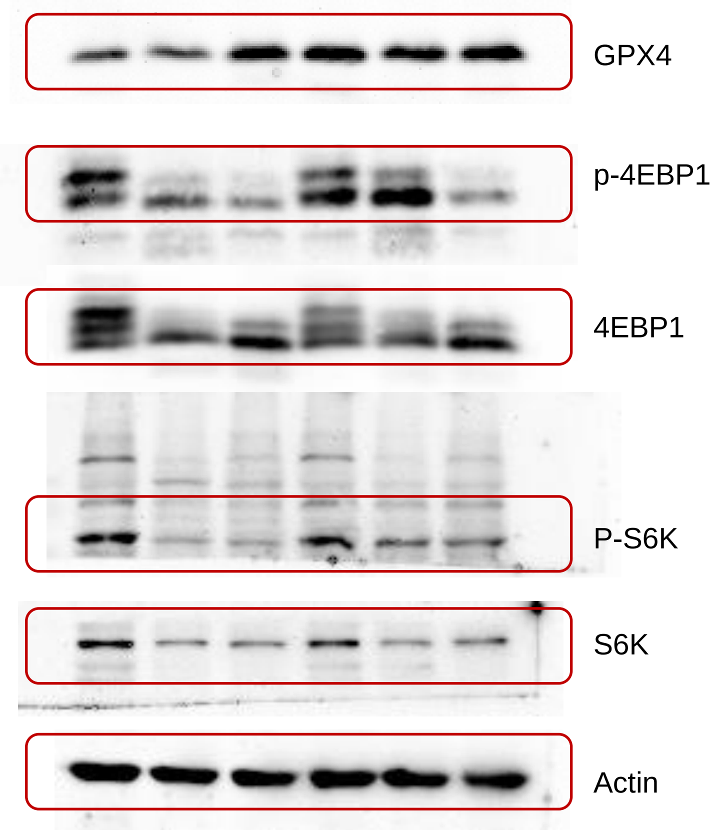
GPX4
p-4EBP1
4EBP1
P-S6K
S6K
Actin

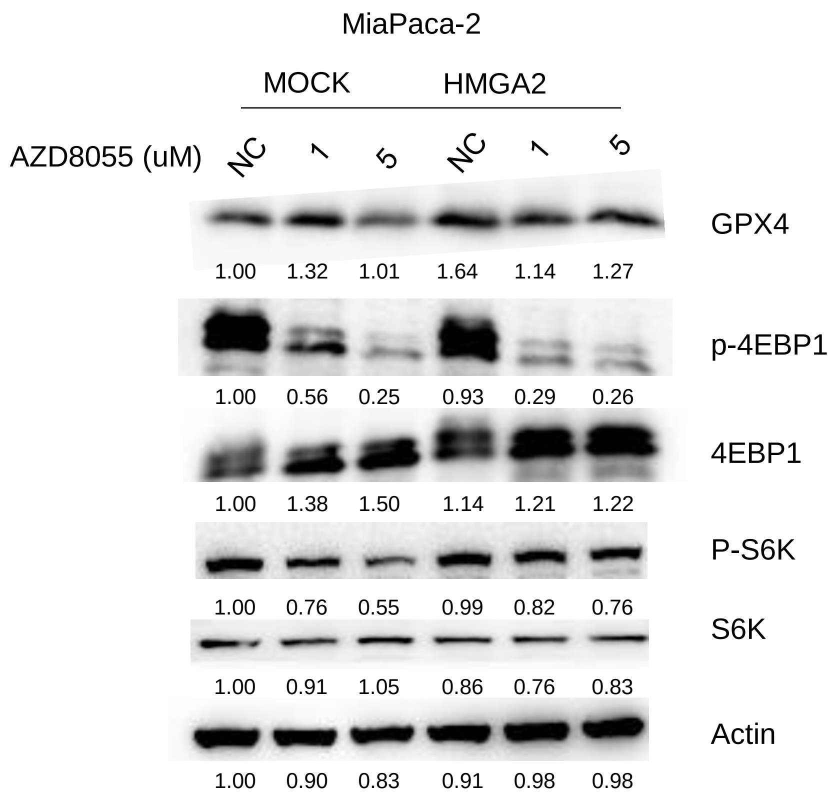
MiaPaca-2
MOCK
HMGA2
AZD8055 (uM)
NC
1
5
NC
1
5
GPX4
1.00 1.32 1.01 1.64 1.14 1.27
p-4EBP1
1.00 0.56 0.25 0.93 0.29 0.26
4EBP1
1.00 1.38 1.50 1.14 1.21 1.22
P-S6K
1.00 0.76 0.55 0.99 0.82 0.76
S6K
1.00 0.91 1.05 0.86 0.76 0.83
Actin
1.00 0.90 0.83 0.91 0.98 0.98

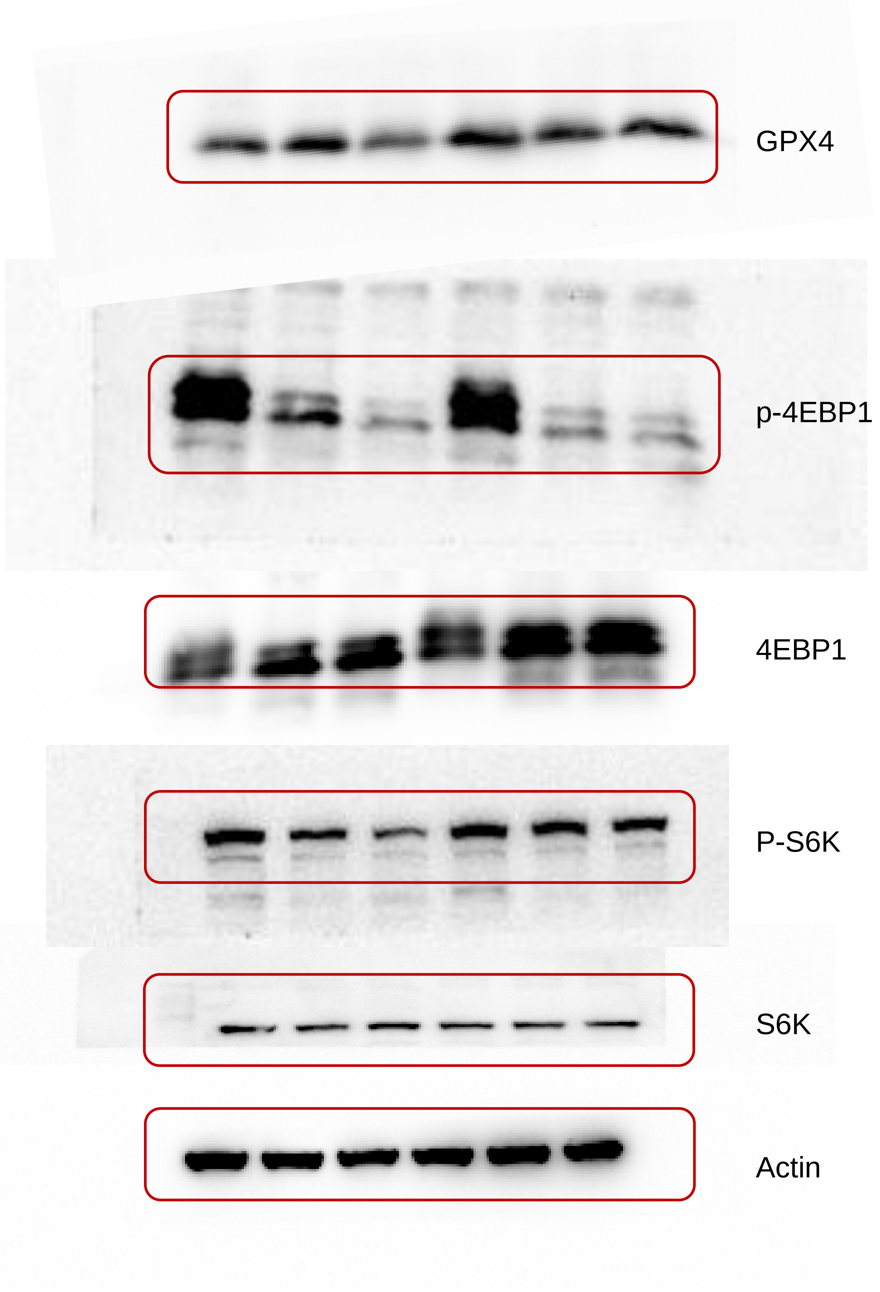
GPX4
p-4EBP1
4EBP1
P-S6K
S6K
Actin

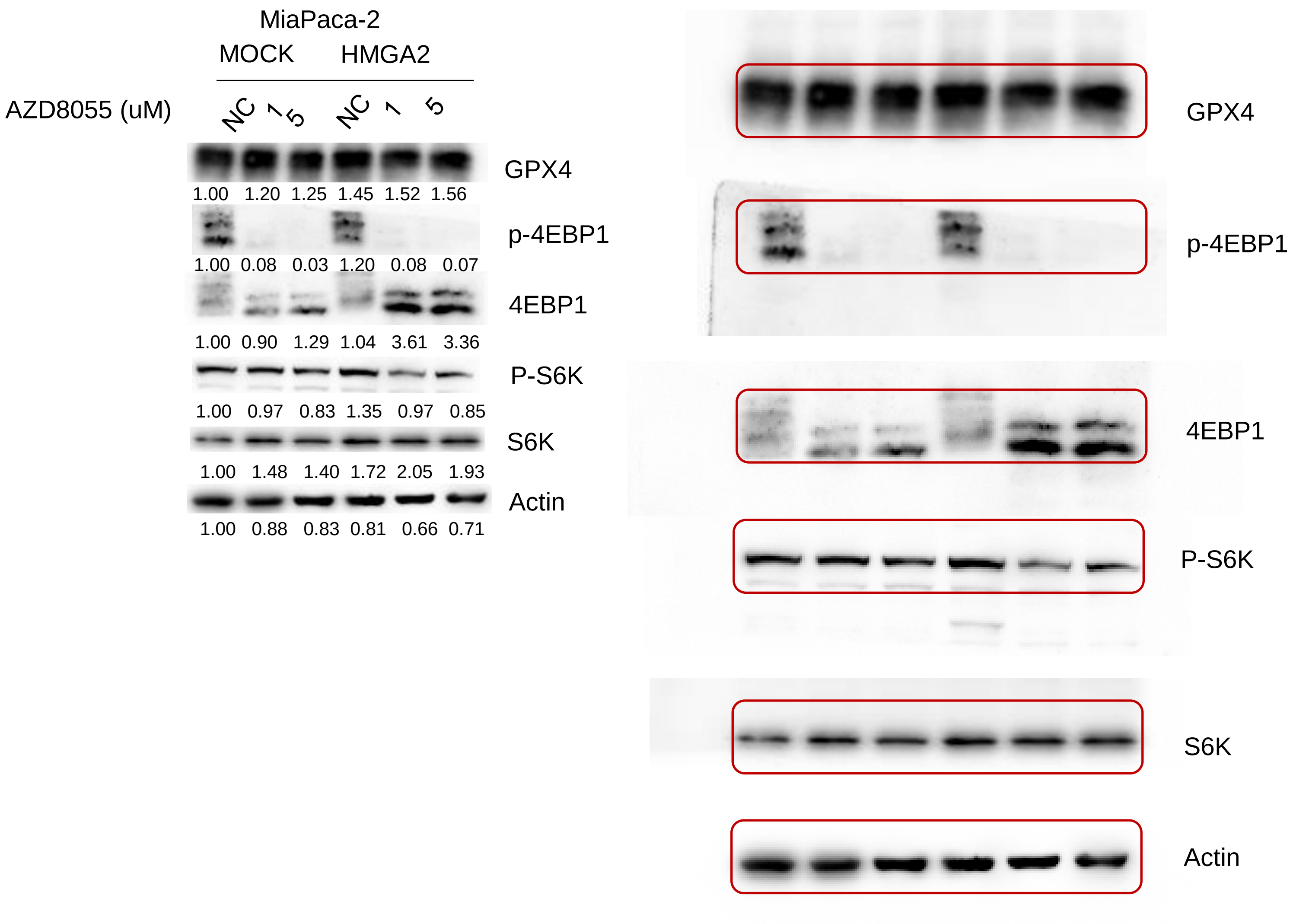
MiaPaca-2
MOCK
HMGA2
AZD8055 (uM)
NC 1 5 NC 1 5
GPX4
1.00 1.20 1.25 1.45 1.52 1.56
p-4EBP1
1.00 0.08 0.03 1.20 0.08 0.07
4EBP1
1.00 0.90 1.29 1.04 3.61 3.36
P-S6K
1.00 0.97 0.83 1.35 0.97 0.85
S6K
1.00 1.48 1.40 1.72 2.05 1.93
Actin
1.00 0.88 0.83 0.81 0.66 0.71
GPX4
p-4EBP1
4EBP1
P-S6K
S6K
Actin

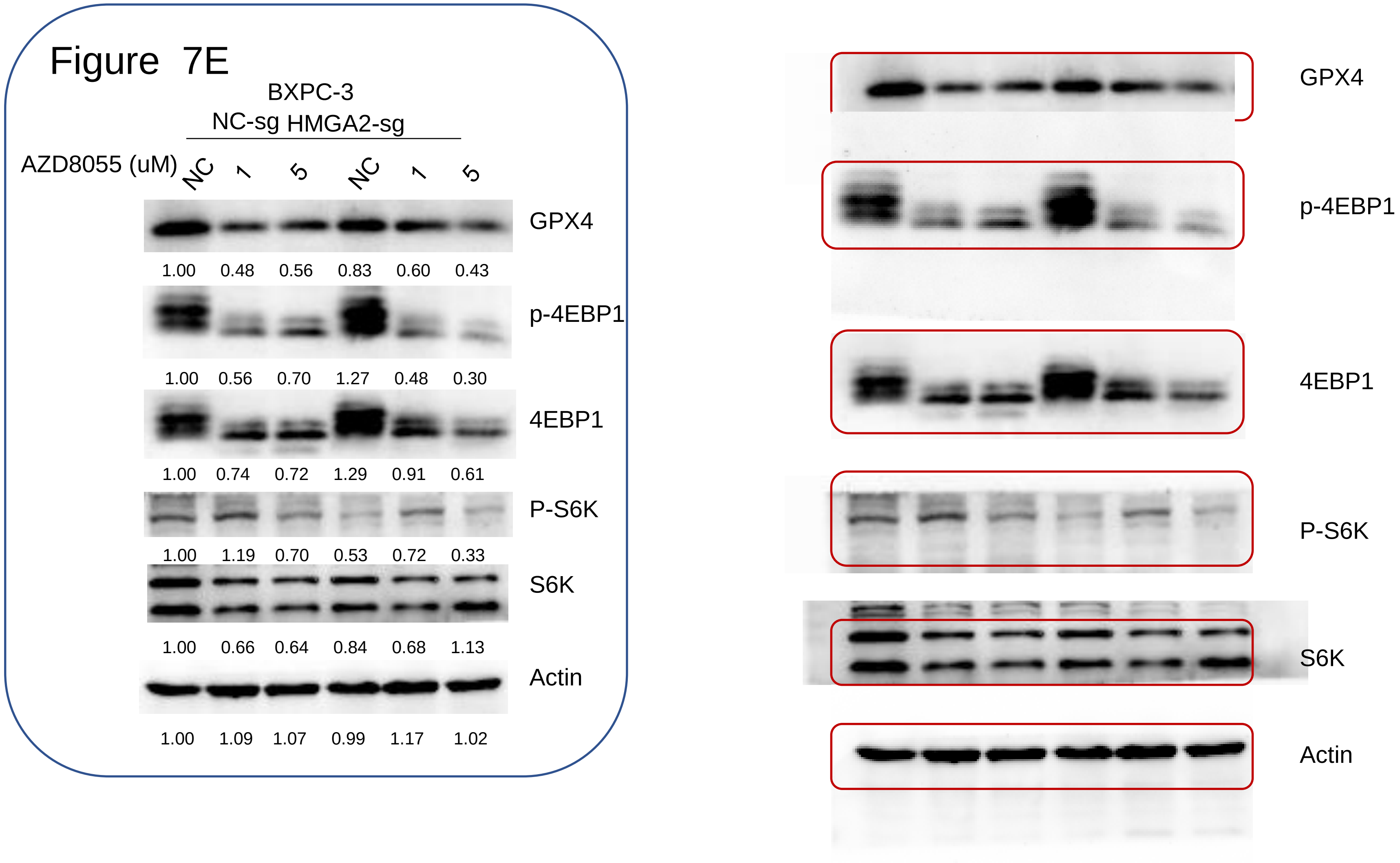

Figure 7E
BXPC-3
NC-sg HMGA2-sg
AZD8055 (uM)
NC
1
5
NC
1
5
GPX4
1.00 0.48 0.56 0.83 0.60 0.43
p-4EBP1
1.00 0.56 0.70 1.27 0.48 0.30
4EBP1
1.00 0.74 0.72 1.29 0.91 0.61
P-S6K
1.00 1.19 0.70 0.53 0.72 0.33
S6K
1.00 0.66 0.64 0.84 0.68 1.13
Actin
1.00 1.09 1.07 0.99 1.17 1.02
GPX4
p-4EBP1
4EBP1
P-S6K
S6K
Actin

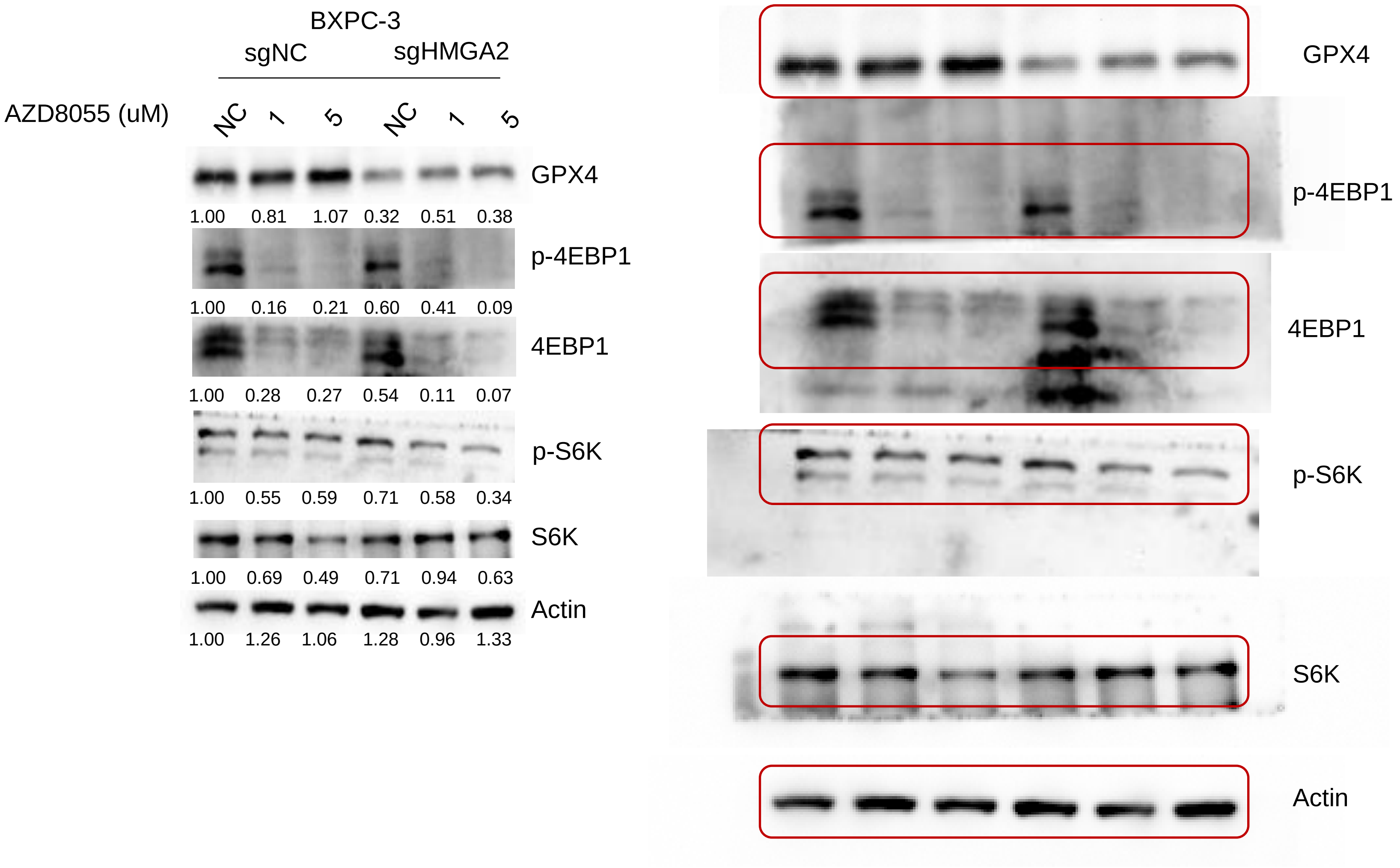

BXPC-3
sgNC
sgHMGA2
AZD8055 (uM)
NC
1
5
NC
1
5
GPX4
1.00 0.81 1.07 0.32 0.51 0.38
p-4EBP1
1.00 0.16 0.21 0.60 0.41 0.09
4EBP1
1.00 0.28 0.27 0.54 0.11 0.07
p-S6K
1.00 0.55 0.59 0.71 0.58 0.34
S6K
1.00 0.69 0.49 0.71 0.94 0.63
Actin
1.00 1.26 1.06 1.28 0.96 1.33
GPX4
p-4EBP1
4EBP1
p-S6K
S6K
Actin

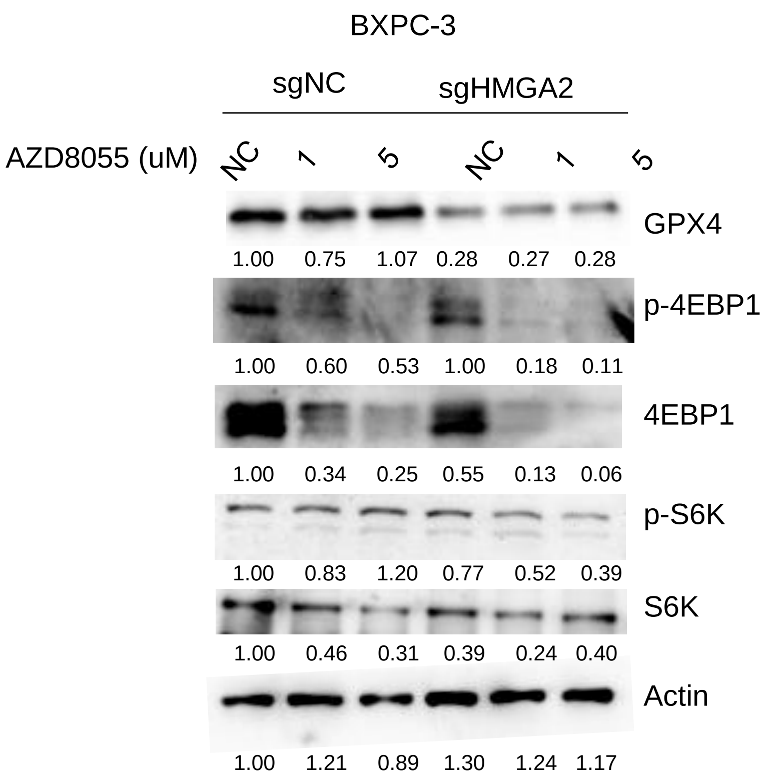
BXPC-3
sgNC
sgHMGA2
AZD8055 (uM)
NC
1
5
NC
1
5
GPX4
1.00 0.75 1.07 0.28 0.27 0.28
p-4EBP1
1.00 0.60 0.53 1.00 0.18 0.11
4EBP1
1.00 0.34 0.25 0.55 0.13 0.06
p-S6K
1.00 0.83 1.20 0.77 0.52 0.39
S6K
1.00 0.46 0.31 0.39 0.24 0.40
Actin
1.00 1.21 0.89 1.30 1.24 1.17

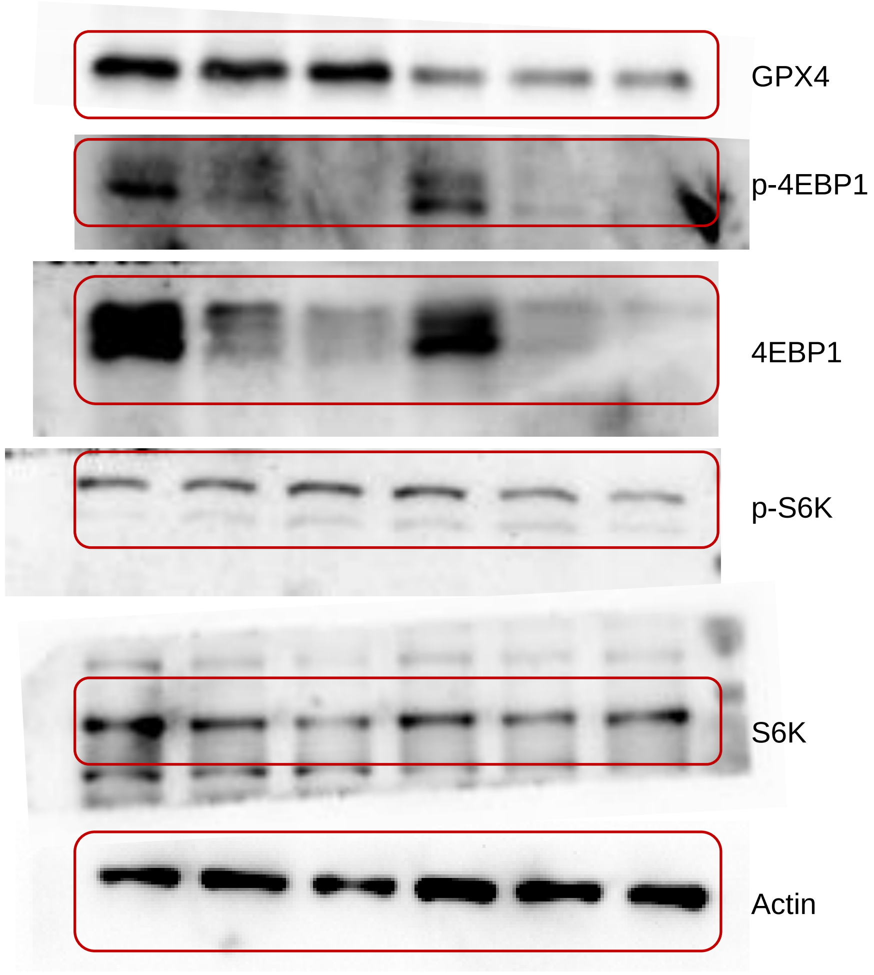
GPX4
p-4EBP1
4EBP1
p-S6K
S6K
Actin

Figure S2B

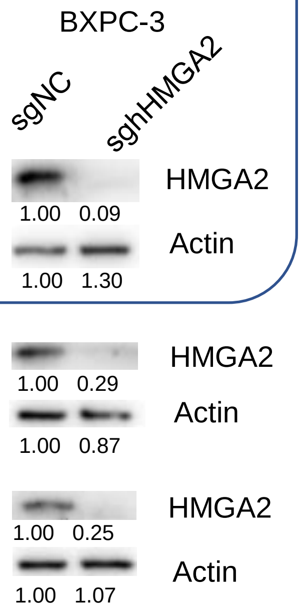

BXPC-3
sgNC
sghHMGA2
HMGA2
1.00 0.09
Actin
1.00 1.30
HMGA2
1.00 0.29
Actin
1.00 0.87
HMGA2
1.00 0.25
Actin
1.00 1.07

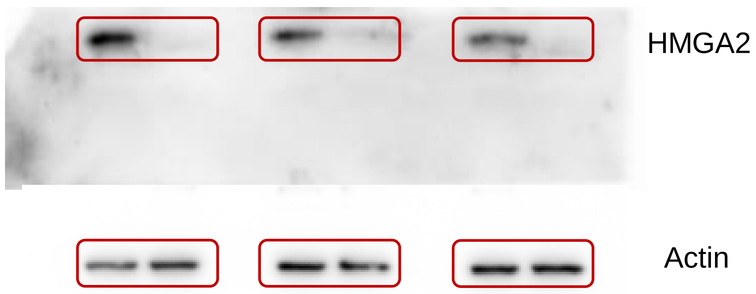

HMGA2
Actin

Figure S4D

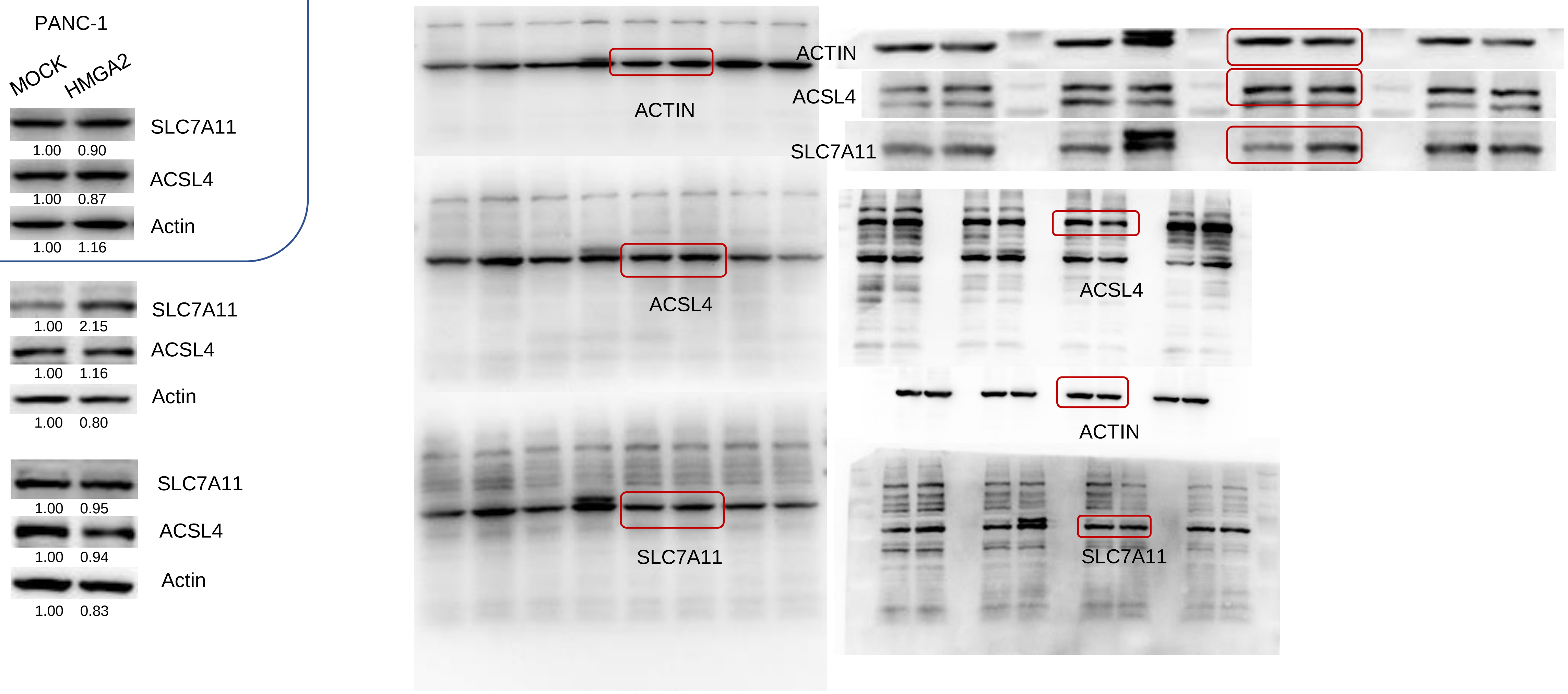
PANC-1
MOCK
HMGA2
SLC7A11
1.00 0.90
ACSL4
1.00 0.87
Actin
1.00 1.16
SLC7A11
1.00 2.15
ACSL4
1.00 1.16
Actin
1.00 0.80
SLC7A11
1.00 0.95
ACSL4
1.00 0.94
Actin
1.00 0.83
ACTIN
ACSL4
SLC7A11
ACTIN
ACSL4
SLC7A11
ACSL4
ACTIN
SLC7A11

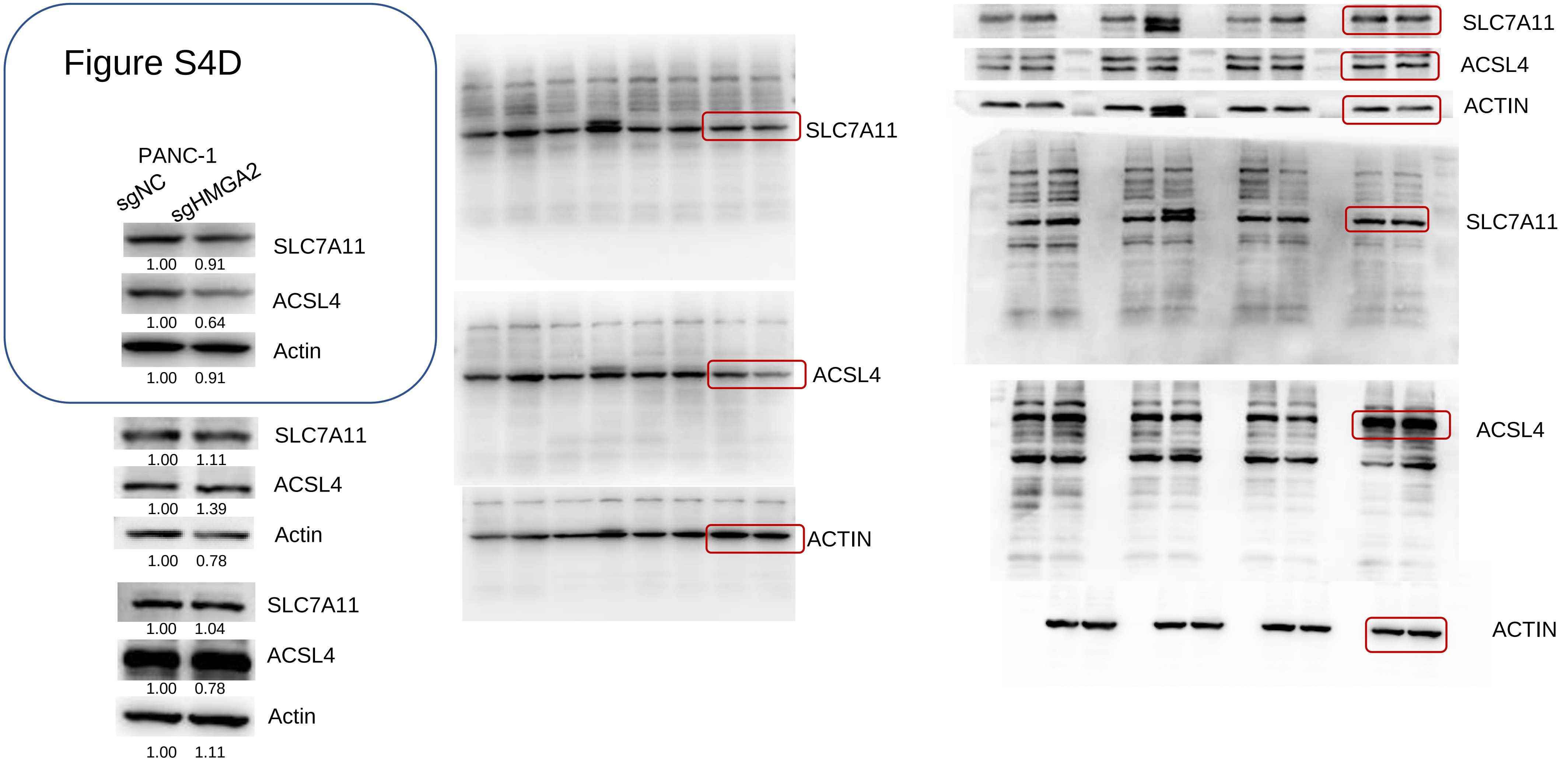
Figure S4D
PANC-1
sgNC
sgHMGA2
SLC7A11
1.00 0.91
ACSL4
1.00 0.64
Actin
1.00 0.91
SLC7A11
1.00 1.11
ACSL4
1.00 1.39
Actin
1.00 0.78
SLC7A11
1.00 1.04
ACSL4
1.00 0.78
Actin
1.00 1.11
SLC7A11
ACSL4
ACTIN
SLC7A11
ACSL4
ACTIN
SLC7A11
ACSL4
ACTIN

Figure S4F

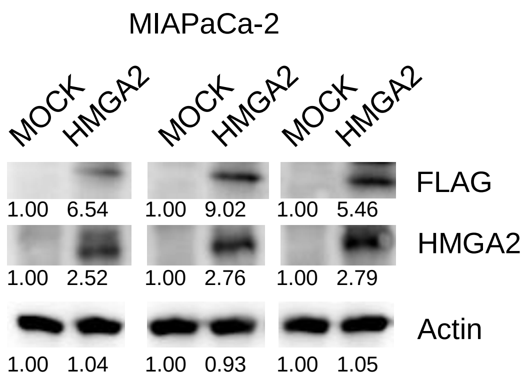
MIAPaCa-2
MOCK
HMGA2
MOCK
HMGA2
MOCK
HMGA2
FLAG
1.00 6.54
1.00 9.02
1.00 5.46
HMGA2
1.00 2.52
1.00 2.76
1.00 2.79
Actin
1.00 1.04
1.00 0.93
1.00 1.05

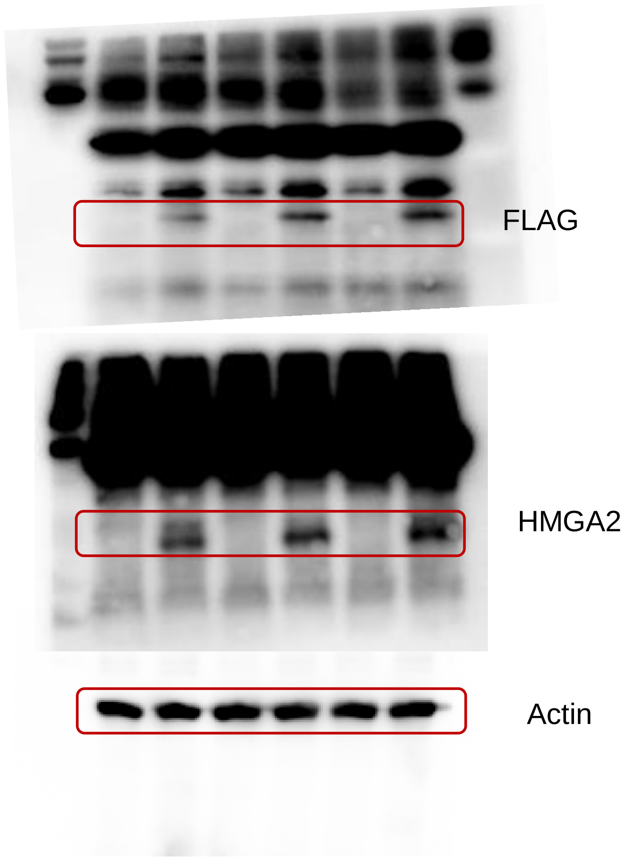
FLAG
HMGA2
Actin

Figure S5A

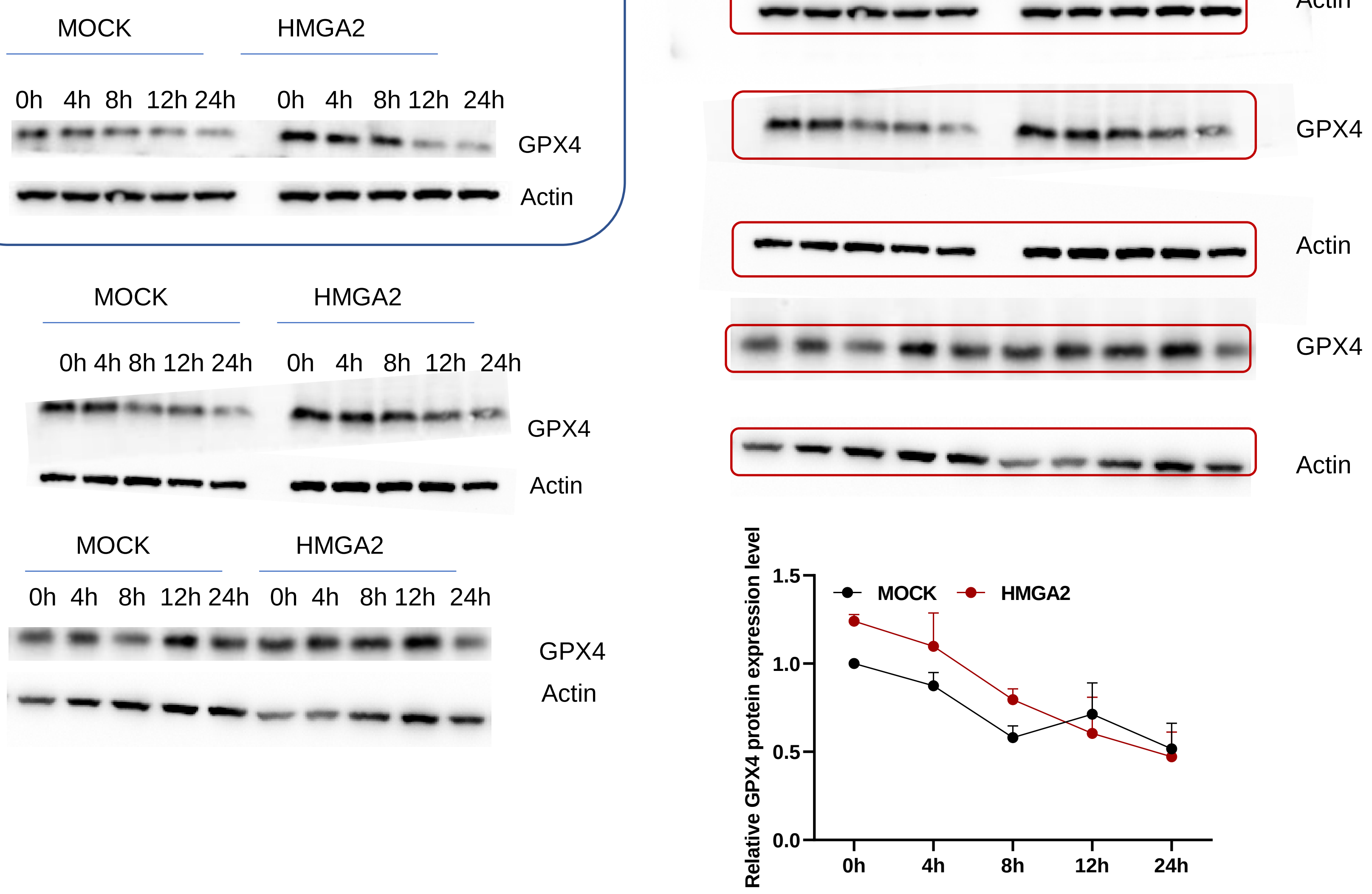
MOCK
HMGA2
0h 4h 8h 12h 24h 0h 4h 8h 12h 24h
GPX4
Actin
MOCK
HMGA2
0h 4h 8h 12h 24h 0h 4h 8h 12h 24h
GPX4
Actin
MOCK
HMGA2
0h 4h 8h 12h 24h 0h 4h 8h 12h 24h
GPX4
Actin
GPX4
Actin
GPX4
Actin
GPX4
Actin
Relative GPX4 protein expression level
1.5
1.0
0.5
0.0
MOCK
HMGA2
0h
4h
8h
12h
24h

Figure S5B

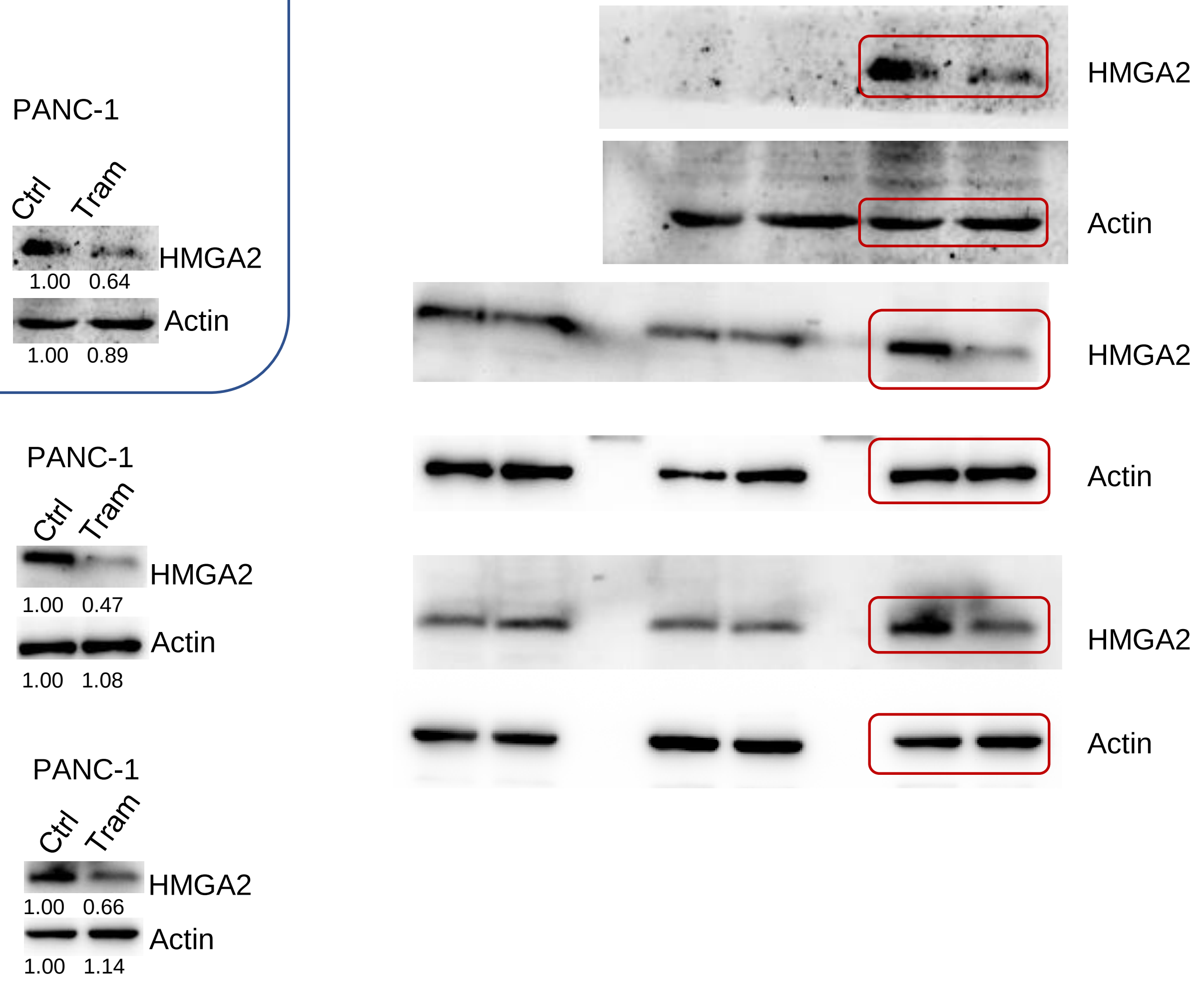
PANC-1
Ctrl
Tram
HMGA2
1.00 0.64
Actin
1.00 0.89
PANC-1
Ctrl
Tram
HMGA2
1.00 0.47
Actin
1.00 1.08
PANC-1
Ctrl
Tram
HMGA2
1.00 0.66
Actin
1.00 1.14
HMGA2
Actin
HMGA2
Actin
HMGA2
Actin

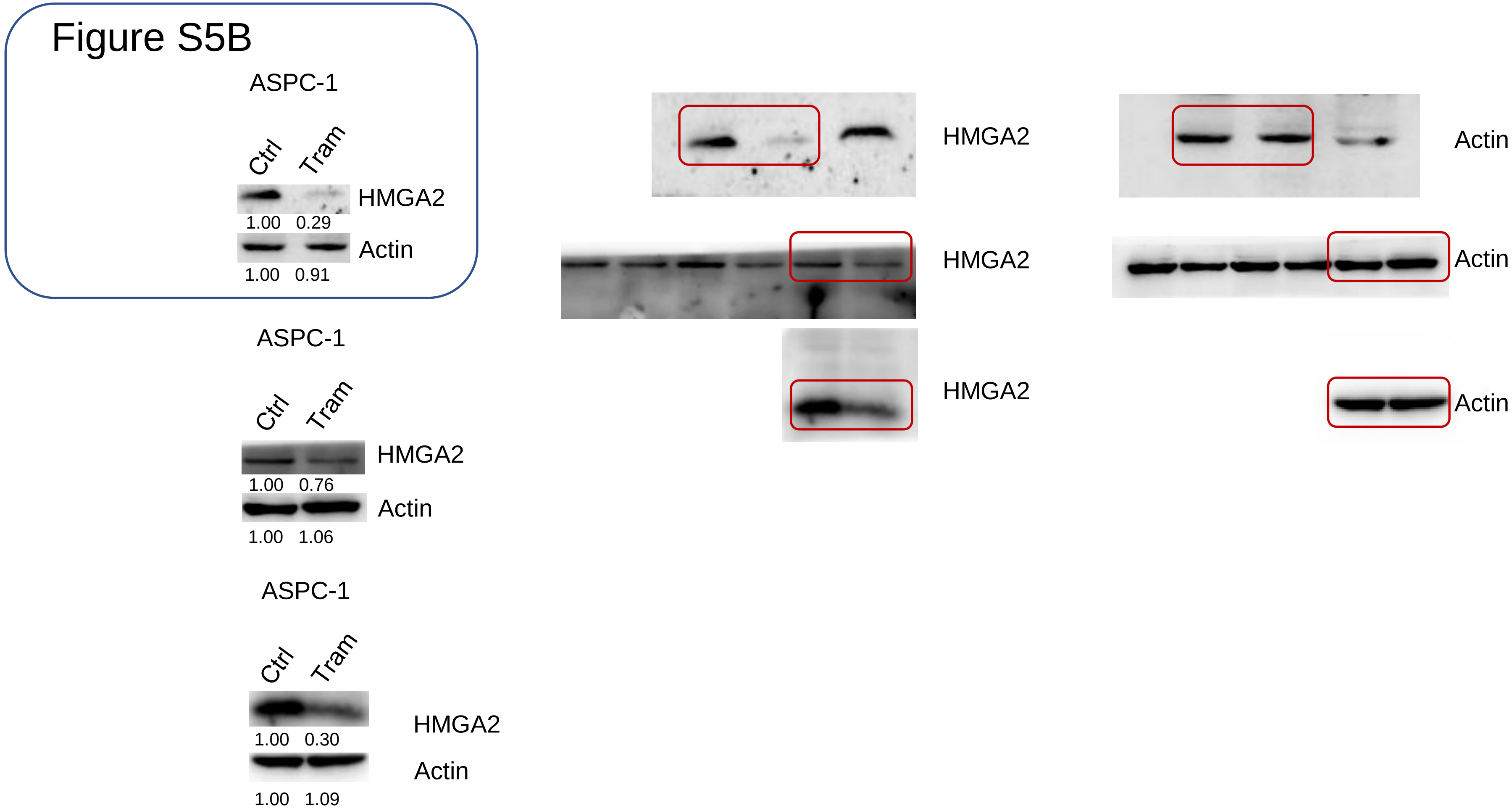
Figure S5B
ASPC-1
Ctrl
Tram
HMGA2
1.00 0.29
Actin
1.00 0.91
ASPC-1
Ctrl
Tram
HMGA2
1.00 0.76
Actin
1.00 1.06
ASPC-1
Ctrl
Tram
HMGA2
1.00 0.30
Actin
1.00 1.09
HMGA2
HMGA2
HMGA2
Actin
Actin
Actin

Figure S5B

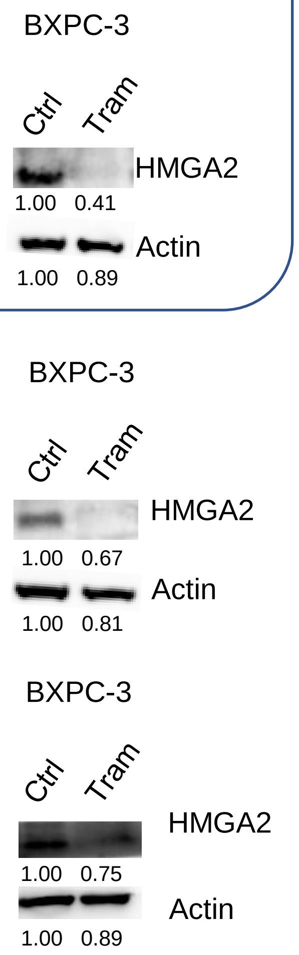
BXPC-3
Ctrl
Tram
HMGA2
1.00 0.41
Actin
1.00 0.89
BXPC-3
Ctrl
Tram
HMGA2
1.00 0.67
Actin
1.00 0.81
BXPC-3
Ctrl
Tram
HMGA2
1.00 0.75
Actin
1.00 0.89

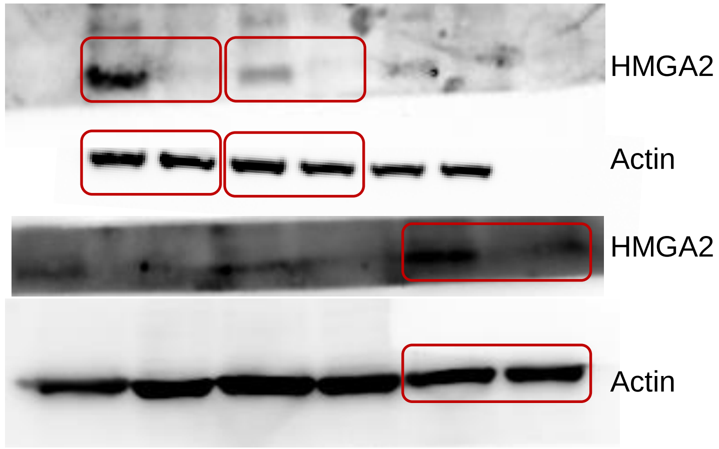
HMGA2
Actin
HMGA2
Actin
